# Metal–Organic Frameworks as Photocatalysts for Solar-Driven Overall Water Splitting

**DOI:** 10.1021/acs.chemrev.2c00460

**Published:** 2022-12-12

**Authors:** Sergio Navalón, Amarajothi Dhakshinamoorthy, Mercedes Álvaro, Belén Ferrer, Hermenegildo García

**Affiliations:** † Departamento de Química, Universitat Politècnica de València, Camino de Vera s/n, Valencia46022, Spain; ‡ School of Chemistry, Madurai Kamaraj University, Palkalai Nagar, Madurai625021, Tamil NaduIndia; § Instituto Universitario de Tecnología Química, CSIC-UPV, Universitat Politècnica de València, Avenida de los Naranjos, Valencia46022, Spain

## Abstract

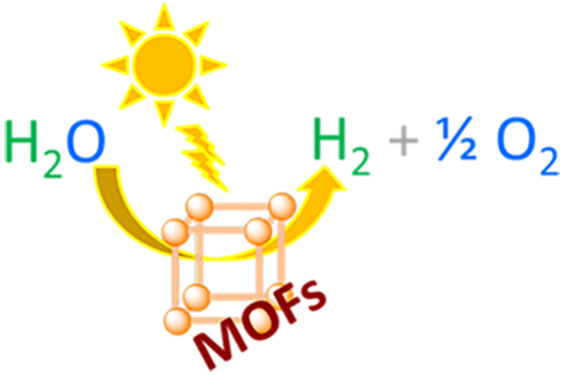

Metal–organic frameworks (MOFs) have been frequently used as photocatalysts for the hydrogen evolution reaction (HER) using sacrificial agents with UV–vis or visible light irradiation. The aim of the present review is to summarize the use of MOFs as solar-driven photocatalysts targeting to overcome the current efficiency limitations in overall water splitting (OWS). Initially, the fundamentals of the photocatalytic OWS under solar irradiation are presented. Then, the different strategies that can be implemented on MOFs to adapt them for solar photocatalysis for OWS are discussed in detail. Later, the most active MOFs reported until now for the solar-driven HER and/or oxygen evolution reaction (OER) are critically commented. These studies are taken as precedents for the discussion of the existing studies on the use of MOFs as photocatalysts for the OWS under visible or sunlight irradiation. The requirements to be met to use MOFs at large scale for the solar-driven OWS are also discussed. The last section of this review provides a summary of the current state of the field and comments on future prospects that could bring MOFs closer to commercial application.

## 1 Introduction

The strong international commitment for achieving a decarbonized society in just a few decades is causing an abrupt shift from the use of fossil fuels to renewable energies, the Sun being possibly the cleanest primary energy source.^1−6^ Estimation of the Sun energy reaching the Earth surface in the order of 1.7 × 10^5^ TW indicates that just a small fraction of this free energy can easily provide the estimated 20 TW needed to the power the planet. However, the low power density of sunlight, the day-night cycles and the influence of seasonal and weather conditions make it necessary to store and accumulate solar energy into so-called solar fuels.^7,8^ Among them, photocatalytic hydrogen production from water is a medium-/long-term viable approach because hydrogen production does not require costly equipment, such as in the case of electrolysis and hydrogen production, can be applied in remote places not connected to high-power electrical grid.^9−11^

Photocatalysis, particularly hydrogen generation from water, has developed since the seminal findings of Fujishima and Honda in 1972, reporting that UV irradiation of a TiO_2_ photoelectrode was able to generate hydrogen.^12^ Subsequently, a large number of studies have focused on the use of inorganic semiconductors for the photocatalytic OWS without electrochemical bias.^10,13−40^ However, in spite of the 50 years that have elapsed since the initial photoelectrochemical hydrogen generation report and the intensive research in the area, the current situation is that still no suitable photocatalyst or photocatalytic process to convert efficiently sunlight into hydrogen has yet been reported.^41−43^ The failure to develop economically viable photocatalytic processes for hydrogen generation is due to a combination of factors, including failure to develop a photocatalyst with strong absorption of all wavelengths in the visible range with efficient charge separation and appropriate alignment of conduction and valence band energies with the redox potentials required for water reduction and water oxidation, adequate lifetimes of charge carriers, etc.^44−46^ While most of the efforts in photocatalysis have been made in studying the activity of various inorganic materials, most of them semiconductors, so far a suitable material has not been found.

This situation is mainly due to the difficulty of engineering the band alignment and light absorption in inorganic materials. For this reason, the use of metal–organic frameworks (MOFs) offering a large flexibility in the selection of constituent metals and linkers with a broad range of electronic properties is so appealing.^47−49^ The purpose of this review is to describe the current state of the art in the use of MOFs as photocatalysts for overall water splitting (OWS), leading to useful hydrogen generation. In comparison with the 50 years of research with inorganic photocatalysts, the first report on hydrogen generation by MOFs was published in 2010,^50^ most of the studies in the area so far paying attention to the ability of MOFs to generate hydrogen in the presence of sacrificial electron donors, neglecting the more difficult process of water oxidation to evolve oxygen that occurs in natural photosynthesis. Probably, the first report on OWS by MOFs was the incorporation of Ni^2+^ in MIL-53(Al)-NH_2_ of Ni^2+^ as cocatalyst being able to activate OWS appeared in 2017.^51^ In only a few years, the number of examples and efficiency regarding OWS promoted by MOFs has increased significantly.

In the first section of the review, the fundamental and general aspects of OWS are briefly commented on, highlighting the accepted targets for solar energy-to-hydrogen generation efficiency borrowed from photovoltaics, the acceptable material durability, and cost required for developing competitive photocatalytic OWS process. Afterwards, the major features and properties of MOFs regarding their use as solar photocatalysts will be commented on. Special attention will be paid to the existing data on the frontier orbitals energy of the most widely researched MOFs as photocatalysts and how these values have been determined. Comparison with inorganic semiconductors, particularly with TiO_2_, will put into the context the role of cocatalysts in MOFs that in general is less important than for other nonporous photocatalysts. The last general introductory section describes the general strategies to improve MOFs photoactivity under sunlight irradiation by preparing multimetallic MOFs, selection of dyes as linkers, or attachment of light harvesting units to satellite lattice positions. Later, selected examples of photocatalytic hydrogen generation will be commended, followed by much less abundant examples of photocatalytic oxygen generation. This will lead to the key section reporting photocatalytic OWS by MOFs, with special emphasis in the most recent achievements and current benchmark photocatalysts. The last section summarizes the review and provides our views on the current targets in the area and promising strategies to overcome present bottlenecks.

## 2 Fundamentals of the Photocatalytic OWS

**Figure 1:**
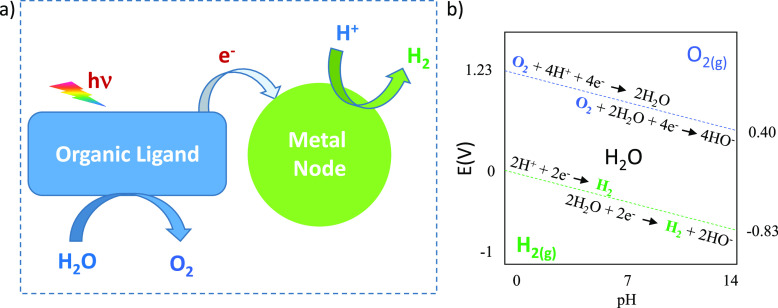
(a) A general mechanism of photoinduced charge separation in MOFs triggered by light absorption and photoinduced charge transfer to the near metal node. (b) Oxygen and proton reduction potentials as a function of the water pH.

OWS is a thermodynamic unfavorable process (Δ*G* > 0) that requires external energy to promote H_2_O decomposition into H_2_ and ^1^/_2_O_2_.^12,26,52−56^ In heterogeneous photocatalysis, the external energy source is light that provides the energy input required for OWS to occur.^21,57−59^ The photocatalytic process is initiated by absorption of photons with a wavelength of equal or higher energy than the band gap. To perform the OWS, the energy of the transient state of the photocatalyst should meet the thermodynamics of H_2_O decomposition to H_2_ and O_2_, reactions whose redox potential is dependent on the water pH (Figure 1). For example, under standard conditions, the minimum band bap of the semiconductor based on thermodynamic requirements should be 1.23 V, with the valence and conduction band potentials at 1.23 and 0 V, respectively (Figure 1). Beyond thermodynamic considerations, an overpotential is required to overcome the activation barrier for the hydrogen evolution reaction (HER) and oxygen evolution reaction (OER) to evolve H_2_ and O_2_ at a measurable rate, respectively.^60^

The efficiency of the photocatalytic process in terms of light energy conversion into chemical energy of the products depends of several factors.^14,21,34,36,58,59,61,62^ The initial photoinduced charge separation occurs in the fs time scale and it results in charge separation. Then the occurrence of fast undesirable electron–hole recombination, either in the site in which charge separation has occurred (“geminate recombination”), after charge carrier migration and random encounter of electrons and holes takes place in the ps–ms timescale. Charge recombination in any of the possible ways is one of the major factors that determine efficiency of the process.^63−65^

Other factor that limits the efficiency of a photocatalyst is the occurrence of the thermodynamically much favored OWS back reaction resulting in H_2_O formation from evolved H_2_ and O_2_ (Δ*G* = −237 kJ mol^–1^).^14,21^ This back reaction is especially important in the presence of cocatalysts such as Pt nanoparticles (NPs) that are well-known promoters of conventional catalytic or even photocatalytic hydrogen reaction with oxygen.^66^ Photocatalytically, electrons and holes can unwantedly react with O_2_ or H_2_, respectively, leading to the formation of O_2_^·–^ and H^+^, respectively.

**Figure 2:**
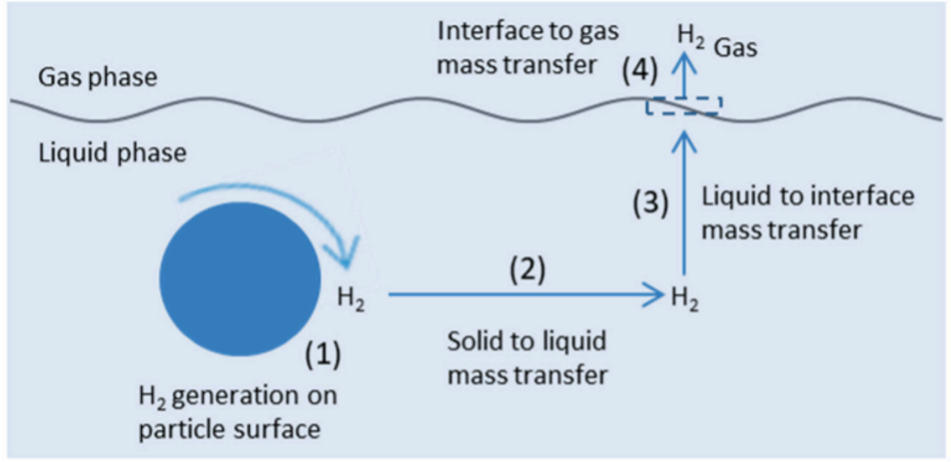
Evolved gases diffusion from the catalyst surface to the gas phase. Reproduced with permission from ref (67). Copyright 2021 MDPI, Switzerland, under Creative Commons CC BY 4.0 license [https://www.mdpi.com/2073-4344/11/1/60].

Although much less studied, mass transfer limitations could also determine the overall photocatalytic efficiency.^67^ Diffusion is particularly important in the case of porous solids in which gas evolution can be influenced by pore size, adsorption capacity, and hydrophilicity as well as stirring speed or reactor design (Figure 2).

## 3 Targets for the Photocatalytic OWS

For many years since Fujishima’s and Honda’s seminal discovery,^12^ photocatalytic H_2_ generation from H_2_O in the presence of sacrificial electron donors has been the subject of study.^16,58,68−70^ However, because the rush for a decarbonized energy has become a top priority, there has been a surge to establish the real possibilities of direct use of solar light to generate H_2_ from H_2_O through photocatalytic OWS.^58,69^ To reach this goal, clear performance indicators have to be reached. The estimated target of a photocatalytic OWS system is to reach an operation life of five years, with a solar energy-to-H_2_ (STH) efficiency between 5% and 10% that would result in an approximate H_2_ cost of ranging from 3.2 to 1.6 USD.^58,71^ The STH indicator^18^ is defined as:

STH = output energy as H_2_ gas/energy of incident solar light = (*r*_H2_ × Δ*G* /*P*_sun_ × *S*) × 100, where *r*_H2_ is the rate of H_2_ production (mmol/s), Δ*G* is the gain in Gibbs free energy (237 000 J/mol), *P*_sun_ is the energy flux of the sunlight (mW/cm^2^), and *S* is the illuminated area of the reactor. Considering the global standard solar spectrum (AM 1.5), the integrated power is 100 mW/cm^2^.

Besides STH, another complementary quantitative indicator of photocatalyst efficiency is the quantum efficiency.

The quantum efficiency (QE) of the photocatalytic OWS is defined as
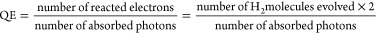


In heterogeneous photocatalysis, due to the difficulty to estimate the number of absorbed photons by a particulate solid photocatalyst in an aqueous suspension in which light reflection and scattering phenomena occurs,^25^ the apparent quantum yield (AQY) at a specific monochromatic wavelength is frequently reported and defined as
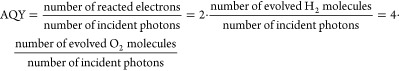


AQY is a performance indicator borrowed from solar cells that can also be very useful in photocatalysis.^25,58^ Real quantum yields could be much higher than AQY and that the AQY values depend on the specific photoreactor and setup.^25^ While this criticism is true, and the dependence of AQY with the experimental setup can complicate direct comparison between photocatalytic activities from different laboratories, it is important to remind that optimization of photocatalytic setups in terms of efficient use of emitted photons is always a mandatory condition of any photocatalytic study. On the other hand, it is becoming more necessary in the field that certified official laboratories that can validate and reproduce data from photocatalysts submitted from research laboratories in a similar manner as it happens in the field of photovoltaic devices.^59^

**Figure 3:**
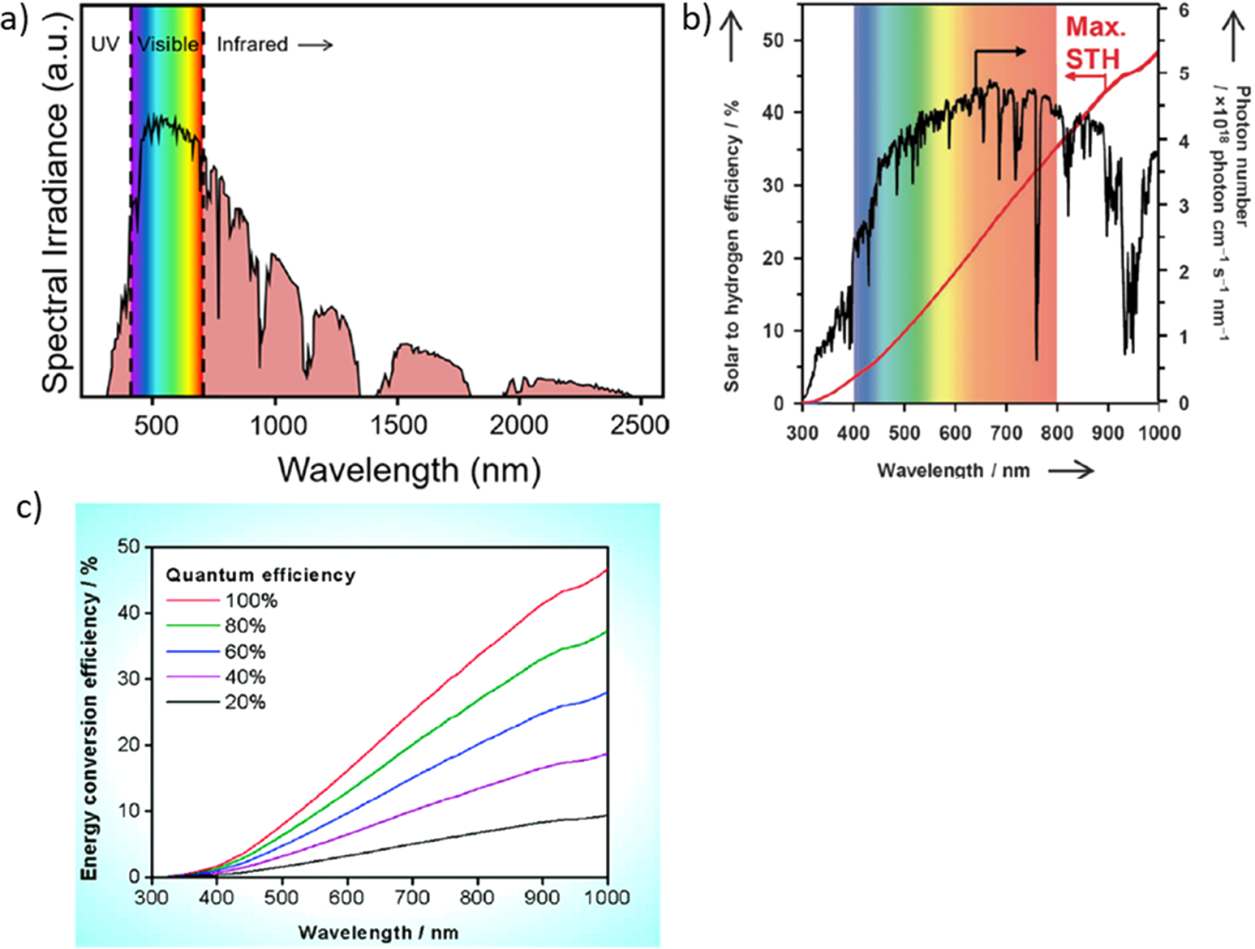
(a) Standard solar radiation spectrum at the Earth surface at sea level. Reproduced with permission from ref (58). Copyright 2020 American Chemical Society. (b) The photons of AM 1.5G filtered standard solar emission as a function of the wavelength and the theoretical maximum STH efficiency integrated from the lowest wavelength to absorption edge assuming a QE of 100%. Reproduced with permission from ref (73). Copyright 2012 Wiley. (c) Calculated STH values as a function of photon wavelength of photocatalyst absorption edge depending on the average QEs. The calculations assume AM 1.5G solar irradiance. Reproduced with permission from ref (10). Copyright 2010 American Chemical Society.

Solar radiation reaching the Earth has a characteristic spectrum constituted mainly by visible and IR radiations as shown in Figure 3a.^58,72^ As commented before, the minimum band gap of a semiconductor to perform the OWS should be 1.23 V that corresponds to a wavelength threshold of about 1000 nm.^65^ Thus, the theoretical maximum STH efficiency considering photons of this energy or higher is approximately 48% (Figure 3b,c).^65^ However, to this minimum thermodynamic value, an overpotential is needed for kinetic reasons that can be reasonably estimated in 0.5 V. Thus, one basic requisite of a photocatalyst to perform the OWS is to have absorption in the visible light range. To reach the target of STH of 10%, a photocatalyst should have a minimum onset absorption of about 526 nm corresponding to a band gap energy of 2.36 eV by assuming a quantum efficiency of 100% for these solar photons that is very unlikely (Figure 3c). As an indication, a photocatalyst with an absorption edge at 700 nm and quantum efficiency of 40% could reach the target STH of 10%. Because the apparent quantum efficiency (AQE) is much lower than 10% and taking into account the needed overpotential, it follows that the MOF should absorb the whole visible region with a molar absorption coefficientto maximaize the efficiency of the OWS process.^71^

## 4 MOFs as Photocatalysts for OWS under Visible Light Irradiation

### 4.1 MOFs Generalities

MOFs are a class of crystalline porous materials constituted by rigid and even flexible organic ligands coordinated through Coulombic and/or coordinative bonds to metal ions or metal clusters.^74−79^ Initially, MOFs were tested for gas adsorption and separation,^80−86^ but immediately the potential of these materials in other areas and particularly for catalysis and photocatalysis was recognized.^87−95^ Particularly, the use of MOFs as photocatalysts has attracted a large interest due to the combination of unique properties that MOFs meet.^96−100^ Among them, the most important ones are light harvesting by tunable organic linkers and efficient charge separation due to the intimate contact between the linkers and nodal metals.^101,102^ The reader is referred to some existing reviews dealing with the MOFs as photocatalysts for different applications other than OWS, including water treatment,^103−106^ pollutant degradation,^88,107−111^ and solar fuels^57,90,112−118^ production among others.^119−123^

### 4.2 MOFs as Photocatalysts

MOFs have been frequently reported as semiconductors by many research groups.^90,101,124−128^ A semiconductor is characterized by a drastic increase in the electrical conductivity upon electronic excitation from the insulating nature of the ground state. This increase in electrical conductivity reflects the mobility of the charge carriers. Experimentally, this increase in electrical conductivity can be measured in different ways, but one of the most useful techniques is transient microwave spectroscopy.^129^ Microwaves do not penetrate in conducting materials, like metals or graphene, but are mostly reflected. By measuring the behavior to the microwaves under light excitation, it has been possible to establish that some of the most common MOF structures remain as insulating materials upon light absorption and, therefore, they do not fall under the classification as semiconductors.^130−132^

However, photocurrent measurements, in which the electrical current in an external circuit is measured under illumination in comparison to the dark, clearly shows that MOFs undergo charge separation in electrons and holes.^99,102,133−135^ Thus, apparently, it is charge mobility in most of the MOFs that is slower than that of semiconductors.^136,137^ Although further understanding on this important issue is needed, it is apparently the mechanism of charge migration that is slower in MOFs as compared to semiconductors.^138,139^

High charge mobility is, in principle, not needed in photocatalysis of porous materials, and it can be even detrimental because it could favor random e^–^/h^+^ recombination.^140^ Fast charge migration can be beneficial in dense, nonporous materials, so the charges can undergo separation and migration to the surface where they can meet cocatalysts or adsorbates. However, in the case of MOFs, the porosity of these materials ensures that every event of charge separation could be effective for reaction because the substrates can be adsorbed in the interior of the pores. In this way, charge migration to the surface that is an elementary step in semiconductors is not strictly needed in the case of MOFs. In a certain sense, the situation in MOFs is identical to molecular metal complexes that may undergo charge separation and promote photochemical reactions without the need to claim charge migration from one complex to others.

Probably, a more accurate and general description of MOFs as photocatalysts is, therefore, to consider them as an ensemble of transition metal complexes held in fixed lattice positions of a porous crystalline framework undergoing in most of the cases photoinduced ligand-to-metal electron transfer, with negligible charge migration. In this regard, many soluble molecular d^0^ and d^10^ transition metal complexes, such as for instance ruthenium(II) trispolypyridyl or zinc porphyrins, are well-known soluble photosensitizers able to trigger hydrogen generation, particularly in the presence of electron donors.^141−145^

Several issues remain poorly investigated, but we think that they are of crucial interest in the area. The first one is the efficiency of charge separation, meaning the quantum yield of the charge separation event.^125^ For soluble transition metal complexes, these quantum yields can be accurately measured and can be as high as 20–30%.^146^ Although for solids, only AQYs can be measured, meaning the ratio between twice the number of hydrogen molecules with respect to the number of photons entering the photoreactor, these numbers are frequently not provided. It is in principal reasonable to consider that the efficiency of photoinduced charge separation should be also high in MOFs because the metal nodes have an excess of positive charges and the common ligands are negatively charged carboxylates. Thus, the electron donation already present in the ground state as a coordination bond would render easily an electron transfer under excitation.

A second issue is whether the photocatalytic reaction should occur necessarily on the external surface, like in dense semiconductors, or photocatalytic reactions may also or preferentially occur inside the solid particles. The higher rate enhancement of cocatalysts incorporated inside the MOF pores as compared to the effect of similar NPs located on the external surface indicates that photocatalytic reactions can occur also inside the particles.^147−150^ In this respect, the photocatalytic activity of the external cocatalysts is probably much higher than the fraction of the external vs the total surface area, indicating that the photocatalytic reactions also occur on the external surface, probably in a preferential manner.

### 4.3 Unique Features of MOFs

One of the unique features of MOFs respect to inorganic semiconductors is the presence of organic linkers connecting to metal ions/clusters.^151−159^ The presence of metal ions and linkers within the polymeric network of MOFs as highly crystalline solids greatly facilitates the ligand to metal charge transfer upon exposure to light.^160−163^ Furthermore, MOFs do exhibit catalytic activity due to the presence of unsaturated metal atoms or active organic ligands^164−168^ In addition, the functional groups in the ligand can either be modified or can be used as sites for the loading of additional active sites.^155,169−173^ Also, the porous structure of MOFs can accommodate photoactive metal complexes/dyes that possess high activity, making MOFs ideal platforms to load homogeneous catalytic centers and employing them as a typical heterogeneous catalyst.^141−143,145,174−178^ Importantly, MOFs offer a considerable flexibility for tuning their chemical, physical, and optoelectronic properties by modification of the organic ligands, metal centers, active functional groups, and particle morphology.^179−185^ Furthermore, due to the existence of a large variety of organic ligands, the flexibility in the design of organic linkers and the rich coordination chemistry of metal centers has made possible the synthesis of over 100 000 MOFs, and many other MOF structures can still be developed for achieving a superior photocatalytic activity.^155,186−191^

### 4.4 Energy Level Positions in MOFs

As commented before, originally, most of the heterogeneous photocatalysts were based on transition metal inorganic semiconductors.^192^ Continuous efforts have been being made to develop accurate and precise techniques to estimate the energy level diagram of inorganic semiconductors.^193−195^ The band gap of a common inorganic semiconductor represents the minimum energy required to excite one electron from the valence band to the conduction band. The energy of the maximal valence and minimal conduction band edges will determine its ability to promote oxidation and reduction reactions after addition of overpotentials to ensure sufficient reaction rate.

**Figure 4:**
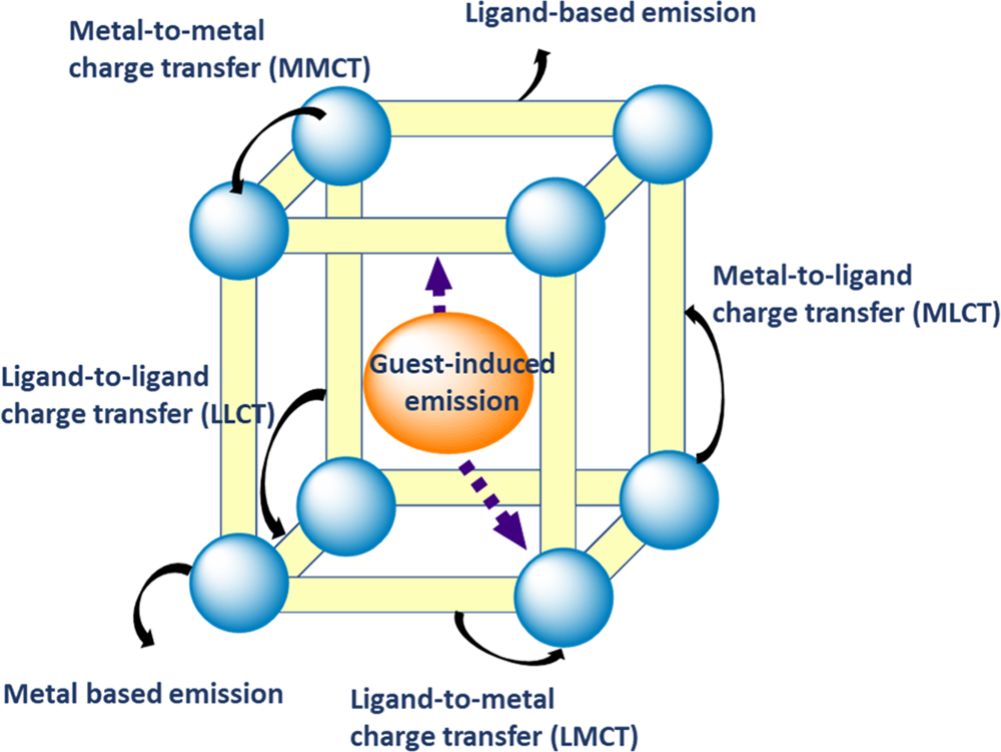
Schematic illustration of some possible photoinduced reaction pathways in MOFs.

As commented in previous sections, MOFs have been considered in many reports as semiconductors, and estimation of their energy level diagrams has been frequently done using methodologies typically applicable for inorganic semiconductors but that may not be the most appropriate for solids with a large internal porosity.^194,196^ In this regard, although MOFs share with semiconductors some elementary steps and, in particular, the generation of a photoinduced charge separation state and, in addition, a certain charge migration can occur in the particle, most MOFs should be better considered as an ensemble of transition metal complexes held in rigid lattice positions in which photoinduced ligand-to-metal electron transfer prevails and the electron mobility is limited (Figure 4).^197,198^ Other alternative excitation mechanism, such as metal-to-ligand, metal-to-metal, or metal-to-metal cluster, can also occur (Figure 4), depending probably on the structure of specific MOFs and the electronic configuration of metal ions and linkers, but these mechanisms are poorly addressed at the moment.

As mentioned previously, one of the most remarkable differences of MOFs respect to inorganic semiconductors is the limited electron mobility of the former.^199^ Thus, several authors have proposed to refer to highest occupied crystal orbital (HOCO) and lowest unoccupied crystal orbital (LUCO), respectively, to the maximum and minimum occupied and unoccupied electronic levels in MOFs. This notation resembles that employed in inorganic semiconductors with valence and conduction bands, and it is closely related to the highest occupied molecular orbital (HOMO) and lowest unoccupied molecular orbital (LUMO) employed in molecular chemistry.^200^ Regardless these considerations, the literature of MOFs is plenty of traditional notation borrowed from inorganic semiconductors.

Considering these precedents, the present paragraph is devoted to discussing the methodologies commonly used in MOFs to determine their energy level diagram, casting some doubts about its correct application in all cases. As for inorganic semiconductors, the band gap in a MOF has been used to denote the minimum required energy to promote an electron excitation from the maximum HOCO and the minimum LUCO resulting in the formation of an electron/hole pair.^201,202^ The minimum thermodynamic value of the band gap in a MOF that allows to perform the photocatalytic OWS is 1.23 V vs NHE. However, assuming certain similarity with electrocatalysis, this thermodynamic value has to be increased ca. −0.13 V for HER and +0.35 V for OER overpotentials for neutral pH values, making in total a reasonable electronic transition energy of 1.71 V vs NHE with the HOCO and LUCO energy well aligned for OER and HER, respectively.^203^

**Figure 5:**
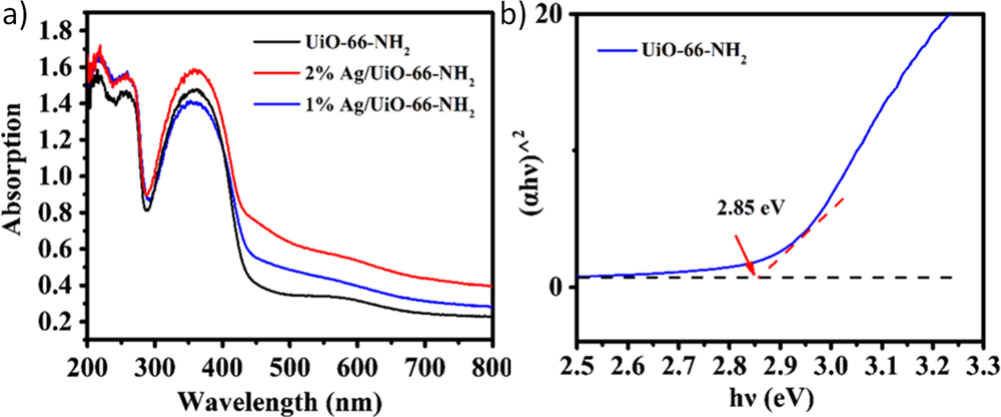
Diffuse reflectance UV–vis spectra (a) and Tauc plot (b) of UiO-66(Zr)-NH_2_ contributing or not Ag nanoparticles as guests. Reproduced with permission from ref (204). Copyright 2019 Springer Nature.

Most of the reports in MOFs have estimated the band gap of a MOF, considering that it is equal to the optical band gap. The optical band gap refers to the minimum energy required of photon to be absorbed by the photocatalyst that would result in the formation of an exciton, in which the electron/hole pairs are electrically attracted, a situation that is different from the formation of independent electrons and holes, because Coulombic interaction energy is more relaxed after charge separation. The optical band bap is lower than the band gap of the material. Nevertheless, in most of inorganic semiconductors, commonly these two values do not differ significantly. In the case of MOFs, the optical band gap is frequently estimated by using the so-called Tauc plot, assuming in most cases that the MOFs are direct semiconductors. To derive the Tauc plot, the diffuse reflectance UV–vis spectrum of the MOF under study is recorded (Figure 5a). Then, the optical band gap of the MOF can be estimated from the following equation,
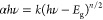
where α is the absorption coefficient, *h* is the Planck constant, ν is the light frequency, *k* is a constant, and *E*_g_ is the band gap.

The value of *n* depends on whether the optical transition of the semiconductor is direct (*n* = 1) or indirect (*n* = 4). For example, in the case of UiO-66(Zr)-NH_2_, the *n* value was considered to be 1.^204^Figure 5b illustrates the estimation of the optical band gap of UiO-66(Zr)-NH_2_ from the Tauc plot, in which the *Y* and *X* axes represent the (α*h*ν)^2^ and (*h*ν) values, respectively. The optical band gap value is determined from the interception between the background line and the tangent of the linear region of the absorption spectrum of the material.

In general, the estimated band gaps for the same MOF in different reports are quite similar. As a representative example, Table 1 shows that estimated optical band gaps for UiO-66(Zr)-NH_2_ as one of the benchmark MOF photocatalysts for water splitting. The bandgap values are quite similar to the mean value about 2.74 ± 0.07 eV.

**Table 1:** Estimated Optical Band Gaps, HOCO, and LUCO Energy Values for UiO-66(Zr)-NH_2_^a^,^b^

band gap (eV)	HOCO (V vs NHE)	LUCO (V vs NHE)	ref
2.85	+2.45	–0.40	(204)
2.76	+2.36	–0.4	(205)
2.65	+2.15	–0.6	(206)
2.82	+2.17	–0.65	(203)
2.92	+1.83	–1.1	(207)

a Optical band gaps estimated from the Tauc plots.

b LUCO values estimated from Mott–Schottky plots.

**Figure 6:**
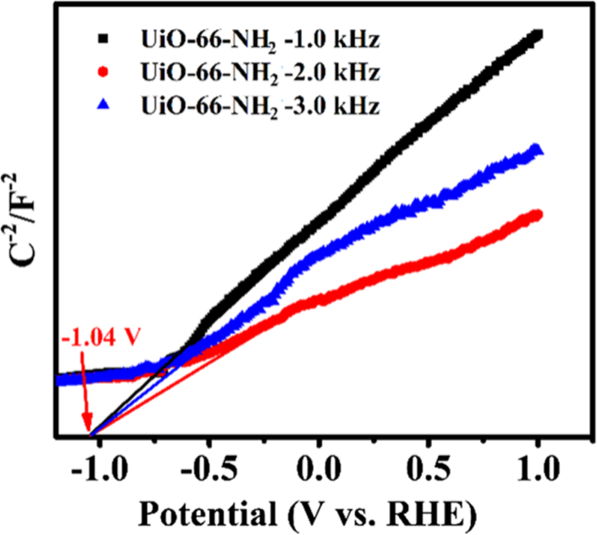
Mott–Schottky plots for UiO-66(Zr)-NH_2_ in Na_2_SO_4_ (0.1 M). Reproduced with permission from ref (204). Copyright 2019 Springer Nature.

For inorganic semiconductors, the estimation of the band edge positions is commonly addressed by using electrochemical methods among other possible experimental techniques.^208^ The Mott–Schottky plot is one of the preferred electrochemical measurements to estimate the flat band potential of inorganic semiconductors.^209^ For this purpose, a conventional three-electrode electrochemical measurement is employed. In several examples, Ag/AgCl electrode and a Pt foil are used as reference and counter electrodes, respectively. The working electrode consists on the MOF deposited onto a fluorine-doped or indium-doped tin oxide conducting glass. Mott–Schottky plot represents the reciprocal of the capacitance square versus the potential (Figure 6) according to the following formula.^209^
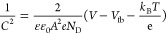
In which *C* is the capacitance, and ε and ε_o_ are the dielectric constant of the medium and the vacuum, respectively, *A* is the electrode area, *e* the electron charge, *N*_D_ is charge carrier density, *V* and *V*_fb_ is the bias potential at which *C* is measured and flat band potential of the semiconductor, respectively, *k*_B_ is the Boltzmann constant, and *T* the absolute temperature.

Frequently, the LUCO energy of many MOFs has been determined using the Mott–Schottky method.^203−207,210,211^Figure 6 illustrates a representative example in which the LUCO band energy of UiO-66(Zr)-NH_2_ was estimated using this theory.^204^ On one hand, the positive slopes observed in Figure 6 were interpreted as the UiO-66(Zr)-NH_2_ behaving as an n-type semiconductor.^203,212,213^ On the other hand, as in typical semiconductors, it was assumed that their conduction band is about 0.1–0.2 V below the flat band potential determined by the Mott–Schottky plot for n-type semiconductors.^212,213^ This assumption has also been employed to determine the LUCO band edge of several other MOFs. Then, considering that the *E*g = *E*_HOCO_ – *E*_LUCO_, the *E*_HOCO_ position of the MOF vs NHE can be determined. Table 1 shows the estimated LUCO values for UiO-66(Zr)-NH_2_ published on different reports. There is a large discrepancy in different studies in which the LUCO energy can differ significantly from −0.4 to −1.1 eV, with a mean value of the reported data and standard deviation of (−0.69 ± 0.30) eV. Again, Mott–Schottky plots assume that MOFs are semiconductors with an electrical field depletion layer at the solid–liquid interface as consequence of the built-in electric field in the semiconductor. A deeper theoretical calculation taking into account the electronic MOF structure will be important to clarify the validity of the experimental data.

In this regard, a recent study has reported the difficulties involved in the determination of the flat band potential of any semiconductor/liquid phase interface by using some of the common electrochemical methodologies reported for this purpose.^214^ In this study, the flat band potential of hematite (Fe_2_O_3_) has been estimated by using Mott–Schottky, Gärtner–Butler analysis, chopped illumination method, and open circuit electrode potential methodologies. The general conclusion of this analysis is that regardless the reproducibility and accuracy of each method, it is highly recommendable the use of multiple methods to determine the flat band potential of a semiconductor. The study also highlights that this situation is of complexity when using nanostructured materials for which some assumptions made to apply specific methodologies deviate from the properties of the real sample due to the structure and particle size of the materials. For example, in the particular case of the Mott–Schottky method when using nanostructured materials, the real surface area of the nano-object can be substantially much larger than the geometric surface area employed in the Mott–Schottky plot.^214,215^ This situation compromises the calculation of capacitance per unit area, resulting in a more negative flat band potential for n-type semiconductors than the true value.^214^ Considering the somehow different methods employed for the preparation of MOFs such as solvothermal, hydrothermal, microwave assisted, mechanochemical, and electrochemical, resulting in the formation of isostructural MOFs based on PXRD, but with significant different surface area values and varied particle morphology, it is reasonable to propose that these discrepancies in surface area will affect to the LUCO estimation. Therefore, it would be a good practice in the case of MOFs to systematically apply different methodologies to determine the most accurate and precise method for estimation of the energy levels in MOFs. In addition, the influence of the preparation procedure resulting in isostructural MOFs according to PXRD, but with differences in terms of particle size, presence of defects, and surface area among other parameters should be studied to shed light on the accuracy of the MOF LUCO values and their variability depending on structural parameters.

An analogous situation as that of the UiO-66 series also occurs when revising the reported energy values of MIL-125(Ti)-NH_2_, other of the most employed MOFs as photocatalyst.^212,216−218^ Similar optical band gap estimation, but large differences in the LUCO values have been reported as shown in Table 2.

**Table 2:** Estimated Optical Band Gaps, HOCO, and LUCO Energy Values Reported for MIL-125(Ti)-NH_2_.^a^,^b^

band gap (eV)	HOCO (V vs NHE)	LUCO (V vs NHE)	ref
2.68	+2.09	–0.49	(216)
2.69	+2.07	–0.62	(217)
2.74	+2.02	–0.72	(212)
2.8	+2.06	–0.74	(218)

a Optical band gaps estimated from the Tauc plots.

b LUCO values determined from Mott–Schottky plot.

**Figure 7:**
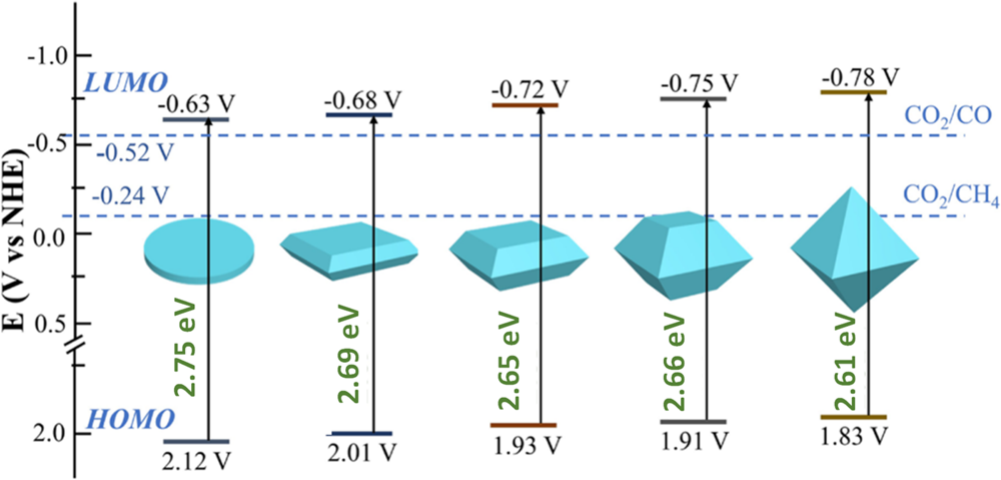
Variation of the HOCO/LUCO energy as a function of facet exposure in MIL-125(Ti)-NH_2_. The standard water reduction and oxidation semi reactions at pH 0 have also been indicated for comparison. Reproduced with permission from ref (219). Copyright 2021 American Chemical Society.

A recent study using MIL-125(Ti)-NH_2_ as a photocatalyst has observed that exposure of different crystal facets of the MOF may determine the resulting energy level of the sample and, therefore, this can influence the resulting photocatalytic activity.^219^Figure 7 presents the remarkable HOCO/LUCO energy value changes that can occur in the same material. The control of the exposed crystal facets was achieved by using specific amounts of acetic acid as modulator and different ratios of MeOH:DMF as solvent. Thus, the resulting MOF morphologies and facet exposure may be also one of the reasons of the different LUCO values reported for this MOF (Table 2). It should be commented, however, that most of the studies using MIL-125(Ti)-NH_2_ as photocatalyst for water splitting do not use organic modulators during the synthesis. Therefore, there is still some uncertainty about if this preferential facet exposure has influenced or not the reported photocatalytic data for this MOF in OWS.

**Figure 8:**
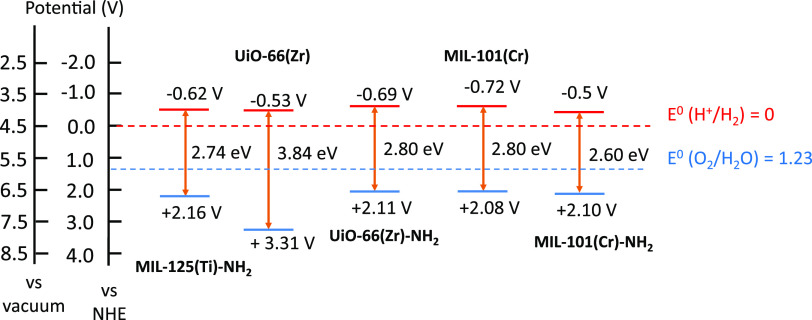
Reported energy levels of some common MOFs employed as photocatalysts for OWS.

When considering Figure 8, one should bear in mind that assuming 100% efficiency in the other mechanistic steps, the 10% STH target requires a MOF bandgap of 2.36 eV corresponding to an absorption wavelength onset in the visible spectrum of 526 nm. These initial constrains on bandgap and band energy alignment, and the available literature data in MOFs suggest that UiO-66 and MIL-125 with appropriate ligand functionalization as well as porphyrin-based MOFs such as PCN-222 are good candidates for the efficient OWS. These MOF families have been the preferred ones in a majority of studies dealing with the photocatalytic HER in water containing a considerable proportion of sacrificial agent under visible light irradiation. To much less an extent, some of these MOFs have been also employed as photocatalysts or components for the preparation of more elaborated hybrid photocatalysts for OWS under visible light irradiation. In the OWS, however, the achieved STH efficiencies are still very low (<0.1%), there being a need to increase the STH value by 2 orders of magnitude.

## 5 Strategies for Improving MOF Photoactivity under Solar or Visible Light Irradiation for the OWS

This section summarizes general strategies that can be employed for the enhancement of the photocatalytic activity of MOFs and that have been used particularly for the OWS.

### 5.1 Functional Group Substitution

**Figure 9:**
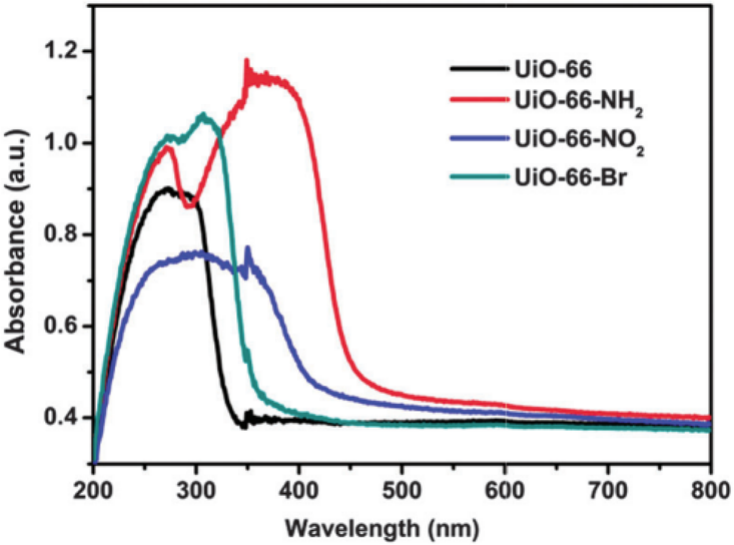
UV–vis absorption spectra of UiO-66-X (X = −H, −NH_2_, −NO_2_, and −Br). Reproduced with permission from ref (224). Copyright 2015 Royal Society of Chemistry.

Light absorption in MOFs is dominated by photon absorption localized at the organic linker, d–d transitions of open shell transition metal ions, and possible charge transfer electron transition either ligand-to-metal or metal-to-ligand.^220−223^ Absorption coefficients in organic ligands can be very high, and they can be predicted based on the rules of organic chemistry for electron conjugation, depending on the number of aromatic rings and multiple bonds. In addition, in the field of MOFs, substitution of organic aromatic ligand with functional groups (−NH_2_, −CH_3_, −NO_2_, −SO_3_H, −SH) shifts the position of the band absorption maxima toward the visible region as consequence of the bathochromic shift of the linker absorption.^90^ As one representative example, Figure 9 shows how the functional groups on the terephthalate aromatic rings modulate the optical absorption properties of UiO-66(Zr)-X (X = −H, −NH_2_, −NO_2_, and −Br) taken as one of the preferred reference solid for photocatalytic OWS.^224^

In the area of photocatalytic hydrogen generation, Garcia and co-workers were among the first showing the higher activity of UiO-66(Zr)-NH_2_ with respect to the parent UiO-66(Zr) material under UV–vis irradiation.^50^ Later, a large number of studies have reported the use of MOFs as photocatalysts for HER,^89,90,119,167,225−248^ confirming the general validity of amino substitution for the development of visible light responsive MOF photocatalysts. This observation can be rationalized considering that the MOF functionalization with -NH_2_ groups is one of the most effective strategies to narrow the band gap by introducing n →π* electronic transitions that require lower energy than the π → π* electron excitation.

Similarly to the case of UiO-66(Zr)-NH_2_, other terephthalate based-MOFs such as MIL-125(Ti) functionalized with −NH_2_ groups exhibit also an absorption band in the visible range, making MIL-125(Ti)-NH_2_ the preferred photocatalyst for many applications.^249−252^ As it will be shown later, UiO-66(Zr) and MIL-125(Ti) functionalized with methylthio groups can be an interesting alternative to −NH_2_ groups for the development of active photocatalysts, with a remarkable change in the properties from basic (−NH_2_) to acid (−SH). When using MOFs bearing −NH_2_ functional groups as photocatalysts for OWS, −NH_2_ groups can be protonated to a various extent depending on the pH values of the aqueous phase. In comparison with −NH_2_, the protonated −NH_3_^+^ group will act now as an electron withdrawing functional group with acid character in such a way that −NH_2_ protonation can modify the photocatalytic activity of the material.^253^

**Figure 10:**
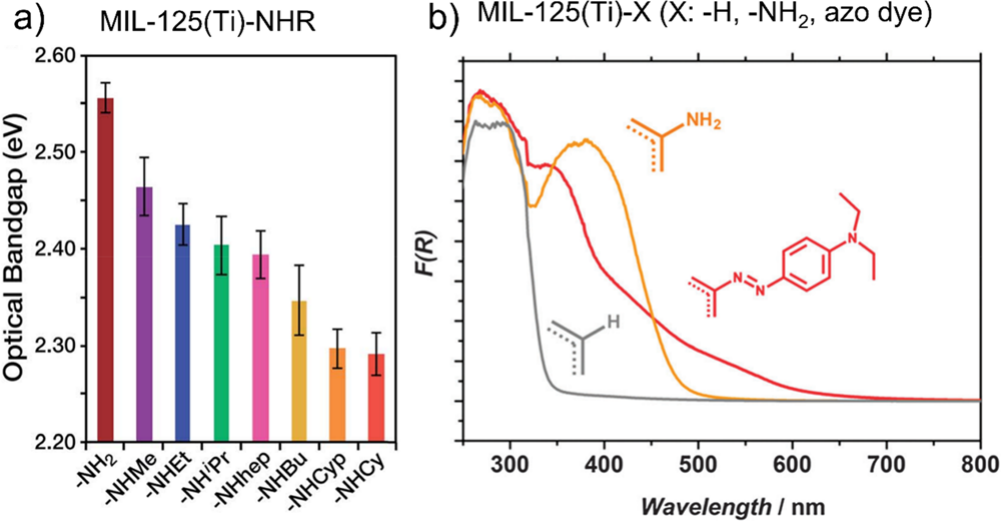
MIL-125(Ti) functionalized with amino derivatives (a). Reproduced with permission ref (254). Copyright 2017 Royal Society of Chemistry. MIL-125(Ti) functionalized with azo dye moiety (b). Cyp and Cy refer to cyclopentyl and cyclohexyl, respectively. Reproduced with permission from ref (255). Copyright 2013 under CC BY 3.0 Royal Society of Chemistry.

Interestingly, MOFs containing −NH_2_ functional groups can be further modified to enhance even more their visible light response (Figure 10). For example, the optical band gap of MIL-125(Ti)-NH_2_ can be still red-shifted by *N*-alkyl^254^ or by molecular dye fragments.^255^ Similarly other studies have also reported the benefits of MIL-125(Ti)-NH_2_ covalent functionalization with organic molecules such as tyrosine to expand further the visible light response of the solid.^202^ Regardless, the considerable progress on the visible light absorption by MOFs more experimental and theoretical studies are required to determine their applicability to bring the efficiency of the OWS higher to the required target.

**Figure 11:**
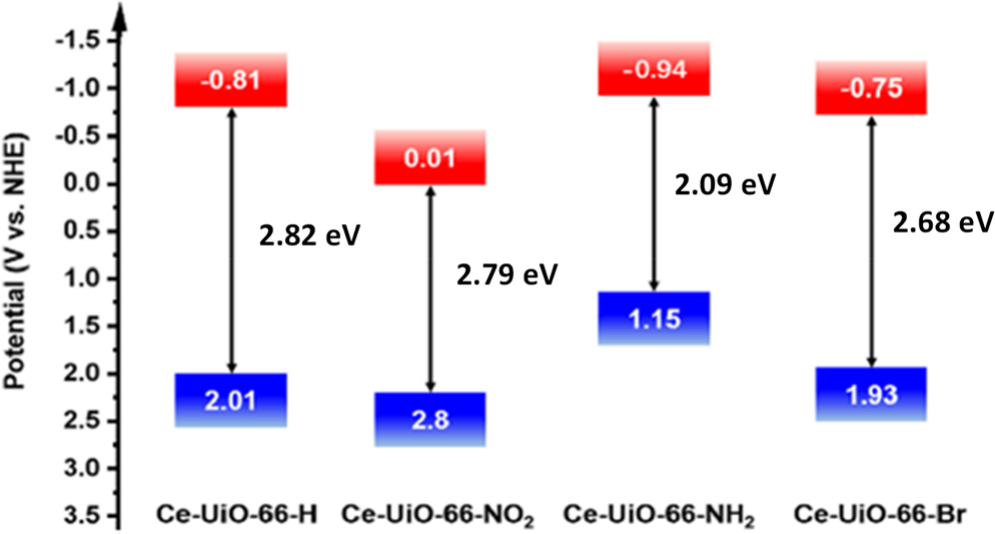
Band energy diagram of UiO-66(Ce)-X. Reproduced with permission from ref (256). Copyright 2019 Wiley.

In a related study focused on UiO-66(Ce)-X derivatives (X being −NO_2_, −NH_2_, or −Br substituents on the aromatic ring), the band gap, HOCO, and LUCO energy values and spatial distribution were estimated.^256^ In this case, the energy level diagram of the Ce MOF solids under study was determined by using the optical band gap measured using the Tauc plot of the diffuse reflectance UV–vis spectra and the Mott–Schottky analysis. Thus, Figure 11 shows the changes of visible light absorption for the case of the UiO-66(Ce)-X series and, consequently, the resulting band gap energy. Importantly, the presence of electron donor functional groups such as −NH_2_ not only diminishes the band gap but also increases the reduction potential of the MOF by shifting the LUCO band toward more negative values vs NHE respect to the nonfunctionalized MOFs. Analogously, UiO-66(Zr) functionalization with −NO_2_ groups diminishes the band bap but, in this case, shifts the HOCO position toward more positive values vs NHE. Regarding substitution on the aromatic ring by functional groups, a matter that has been poorly addressed so far is the photochemical stability of these functional groups upon continuous irradiation because it is well-known in organic photochemistry that some functional groups, such as −NO_2_, are highly reactive upon irradiation, resulting in the formation of nitroso (NO) group.

To achieve high efficiency in photocatalytic OWS, it is important to tune both the HOCO and LUCO values to reach a perfect alignment with the thermodynamic potential for HER and OER. For example, in the case of UiO-66(Ce) MOFs, considering their band gaps and band alignments, it seems that the UiO-66(Ce)-Br should be the most appropriate MOF of the UiO-66(Ce) series as a photocatalyst for the OWS under visible light irradiation. In this case, the use of UiO-66(Ce)-NH_2_ has very small overpotential to perform the thermodynamically and kinetically demanding OER. To develop a UiO-66(Ce) photocatalyst that may reach a possible STH of 10% under visible light irradiation, a maximum band gap of 2.36 eV and appropriate band alignment for OWS is required. Thus, the use of terephthalate mixed ligands with −NH_2_ and/or −Br substituent can be a valid strategy to obtain the MOF with appropriate band gap energy and band alignment. The previous discussion about the flexibility in design and relatively easy fine-tuning by functional group substitution is what makes MOFs as excellent versatile candidates for the design of efficient OWS photocatalysts.

### 5.2 Metal Node Composition

As commented earlier, MOFs combine in a certain way the characteristics of organic and inorganic photocatalysts. The previous section has remarked the possibility of increasing visible light absorption in MOFs through modification of the organic component. This section illustrates some examples with the importance of the metal node composition as a promising strategy to further increase the photocatalytic activity of MOFs for the OWS. As it is shown below, this enhancement is a consequence of the occurrence of a more efficient photoinduced charge separation due to a better orbital overlap between metal nodes and organic linkers, together with an increase of visible light absorption.

**Figure 12:**
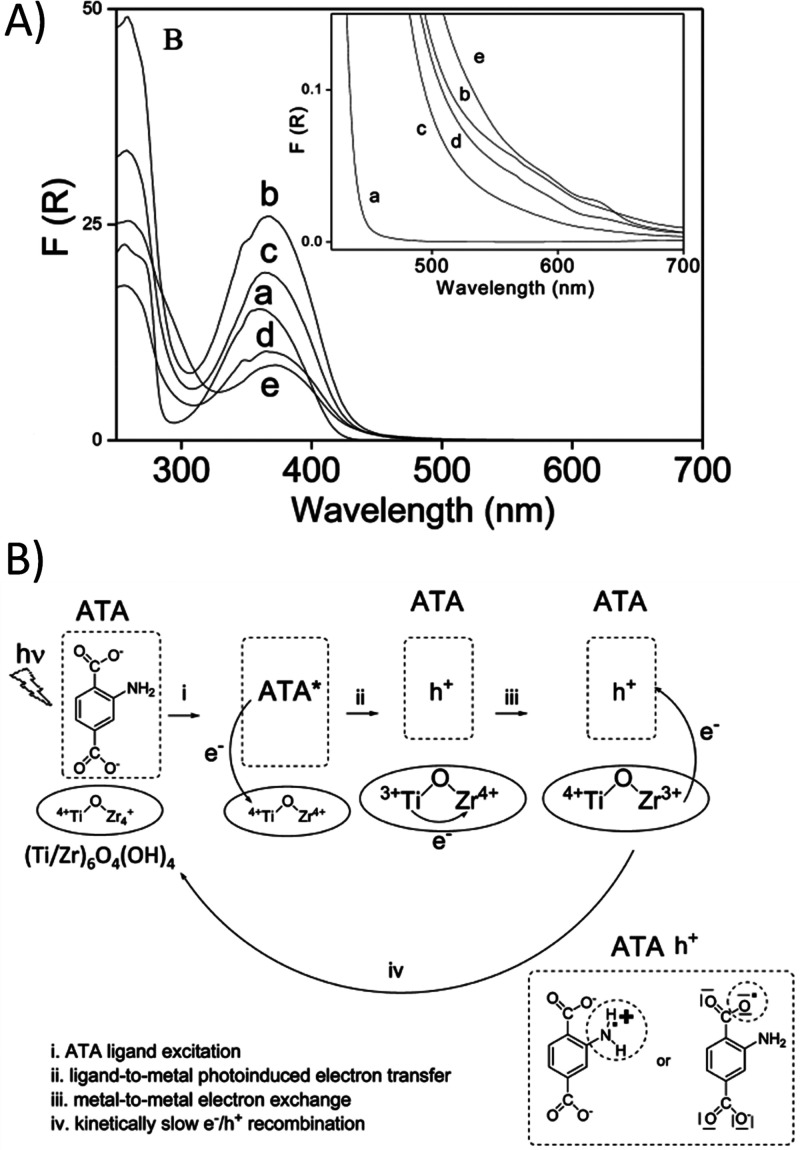
(A) UV–vis absorption spectra of UiO-66(Zr)-NH_2_ (a) and mixed-metal UiO-66(Zr/Ti)-NH_2_ with percentages of Ti 17.6 (b), 25 (c), 28.5 (d), and 35 (e), respectively. The inset shows a magnification of the absorption band onset. (B) Proposed rationalization of the influence of the presence of Ti^4+^ on the photocatalyst UiO-66(Zr/Ti)-NH_2_. Reproduced with permission from ref (260). Copyright 2017 American Chemical Society.

One of the most popular MOFs employed due to its robustness and convenient synthesis has been the UiO-66(Zr)-NH_2_.^232^ The relatively limited photoactivity of UiO-66(Zr)-NH_2_ has been attributed to the inefficient photoinduced electron transfer from the excited organic ligand to the Zr–O cluster.^257,258^ The poor overlap of HOCO and LUCO, both orbitals mostly localized on the organic linker due to the low-lying energy of Zr^4+^ or Hf^4+^ atomic orbitals, make photoinduced ligand to metal electron transfer inefficient. In a series of studies^252,258^ using transient absorption spectroscopy and electron paramagnetic resonance spectroscopy, it was observed that the lifetime of the excited states for UiO-66(Zr)-NH_2_ and UiO-66(Hf)-NH_2_ was short in comparison with MIL-125(Ti)-NH_2_. This shorter lifetime attributed to exciton localization on the ligand was correlated with a much lower photocatalytic efficiency for Zr^4+^ and Hf^4+^ as electron acceptors from the ligand in its excited state, even though these metal ions have the same d^0^ electronic configuration as Ti^4+^. In a seminal work, Li and co-workers showed that the use of a bimetallic UiO-66(Zr/Ti)-NH_2_ material prepared by post-synthetic partial exchange of Zr^4+^ by Ti^4+^ exhibited superior photocatalytic activity than the parent UiO-66(Zr)-NH_2_ for HER in the presence of triethanolamine (TEOA) as sacrificial electron donor under visible light irradiation (λ > 420 nm).^259^ Theoretical calculations have shown that the empty d orbitals of the Ti^4+^ ions significantly contribute to the LUCO of the mixed-metal UiO-66(Zr/Ti)-NH_2_, therefore, promoting the photoinduced electron transfer from the linker to the metal node when Ti^4+^ is present. Later, Li and García have conducted experiments based on transient absorption spectroscopy studies to establish that the Ti^4+^ ions present in the mixed-metal MOF act as mediators during the photoinduced electron transfer, as evidenced by observation of a growth in the transient signal of the charge separated state after the laser pulse that was attributed to the Ti^3+^ to Zr^4+^ electron migration in the nodes.^260^Figure 12 shows that the UiO-66(Zr/Ti)-NH_2_ solids with increasing amount of Ti atoms exhibit in UV–vis absorption spectroscopy an extra shoulder in the red side of absorption band that was attributed to the electronic interaction of the organic ligand and the Ti^4+^ atoms. The experimental evidence was interpreted by considering that the initial electron transfer would occur from the organic ligand excited state to the Ti^4+^ ions of the metal node, forming an intermediate Ti^3+^-O-Zr^4+^ that further transforms into Ti^4+^-O-Zr^3+^ (Figure 12b). Later, similar enhancement of photocatalytic activity was also reported for UiO-67(Hf/Ti) solid for the HER under UV–vis irradiation, showing that the control of the metal node composition is a general strategy to enhance the photocatalytic activity that is still currently underexploited.^261^

**Figure 13:**
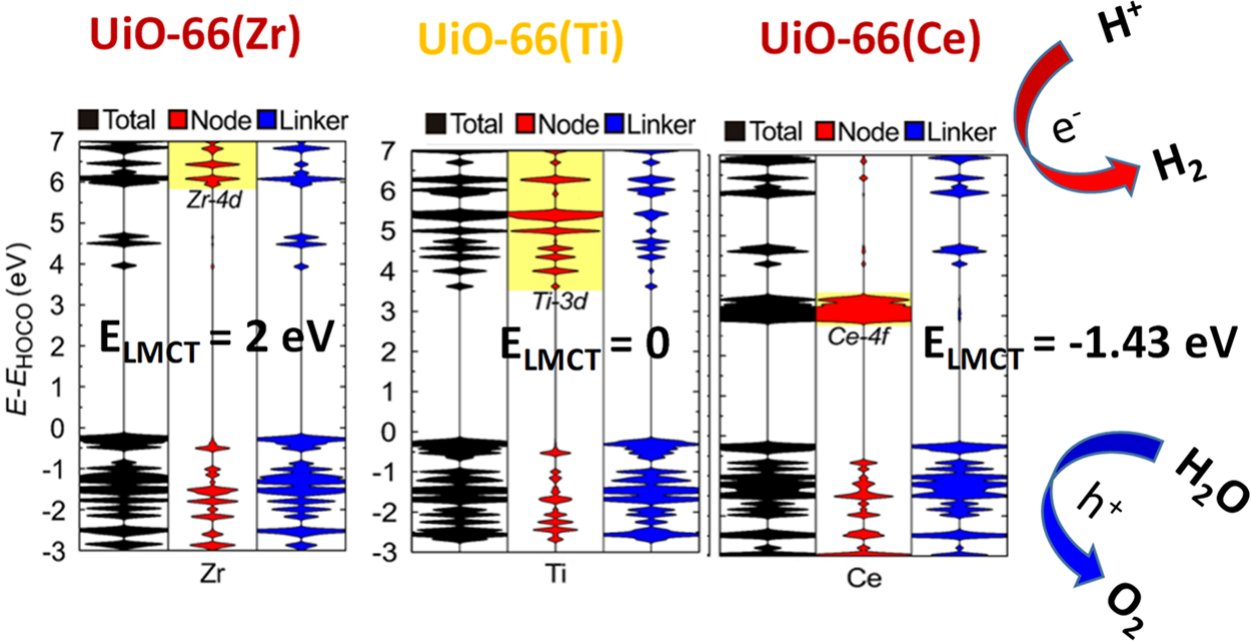
Total (black) and projected (red and blue) density of states of the pristine UiO-66(M) with M = Zr, Ti, or Ce. The unoccupied Zr 4d orbitals, Ti 3d orbitals, and Ce 4f orbitals are highlighted with a yellow background. For the UiO-66(Ce), the occurrence of photocatalytic HER and OER taking place in the LUCO and HOCO has been illustrated. Reproduced with permission from ref (257). Copyright 2018 American Chemical Society.

**Figure 14:**
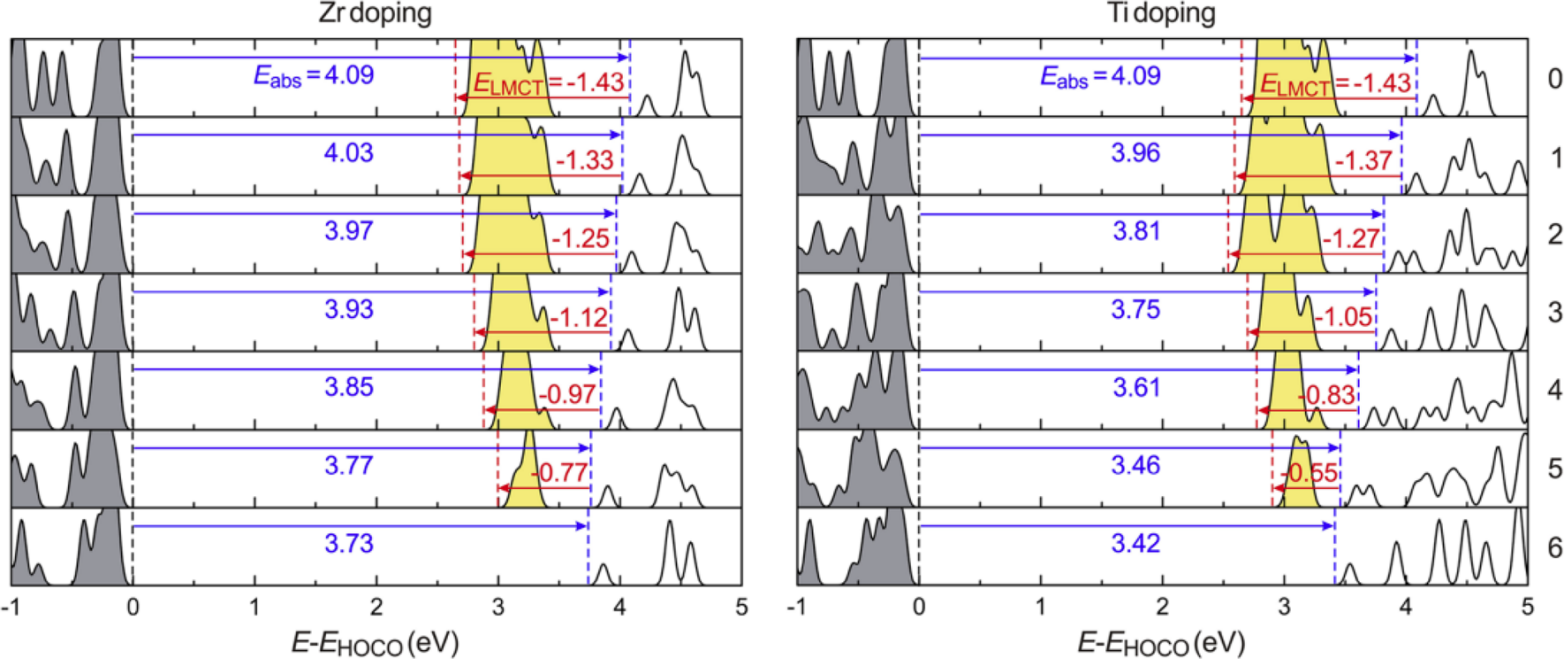
Evolution of the density of states from UiO-66(Ce) to UiO-66(Zr) and from UiO-66(Ce) to UiO-66(Ti) upon metal doping. The numbers of doping ions (Zr or Ti ions) per node are given on the right. The occupied and the unoccupied 4f orbitals are filled with gray and yellow, respectively. The HOCO is on the linker in all cases. The LUCO is on Ce for the Ce-containing MOFs, on the linker for the UiO-66(Zr), and on both linker and Ti for UiO-66(Ti). The energy levels of the HOCO, the lowest unoccupied 4f orbital, and the lowest unoccupied linker orbital are indicated by black, red, and blue dashed lines, respectively. The blue and red arrows indicate *E*_abs_ and *E*_LMCT_, respectively; the values of *E*_abs_ and *E*_LMCT_ are given as well. Reproduced with permission from ref (262). Copyright 2019 American Institute of Physics.

More recently, Wu, Gagliardi, and Truhlar studied by theoretical calculations the influence of the nature of the metal and partial substitution on the UiO-66 nodes for different transition metal ions (M = Zr^4+^, Hf^4+^, Th^4+^, Ti^4+^, U^4+^, or Ce^4+^) on the resulting electronic properties of the UiO-66 structure in terms of band gap, energy level, and spatial electron distribution of HOCO and LUCO.^257^ In comparison to the UiO-66(Zr) solid (Figure 13), this study proposed that the UiO-66(Ce) should be the most efficient photocatalyst for the OWS under visible light irradiation due to the favorable photoinduced ligand-to-metal charge transfer from the organic ligand to the metal cluster. Later, the same authors also proposed by theoretical calculations that Zr^4+^ or Ti^4+^ doping in UiO-66(Ce) solid facilitates the absorption of visible light (Figure 14).^262^ While experimental photocatalytic data that support this prediction is still missing, an unforeseen problem to be solved for an implementation of UiO-66(Ce) is the limited photostability of the material that should hamper its use for OWS.

**Figure 15:**
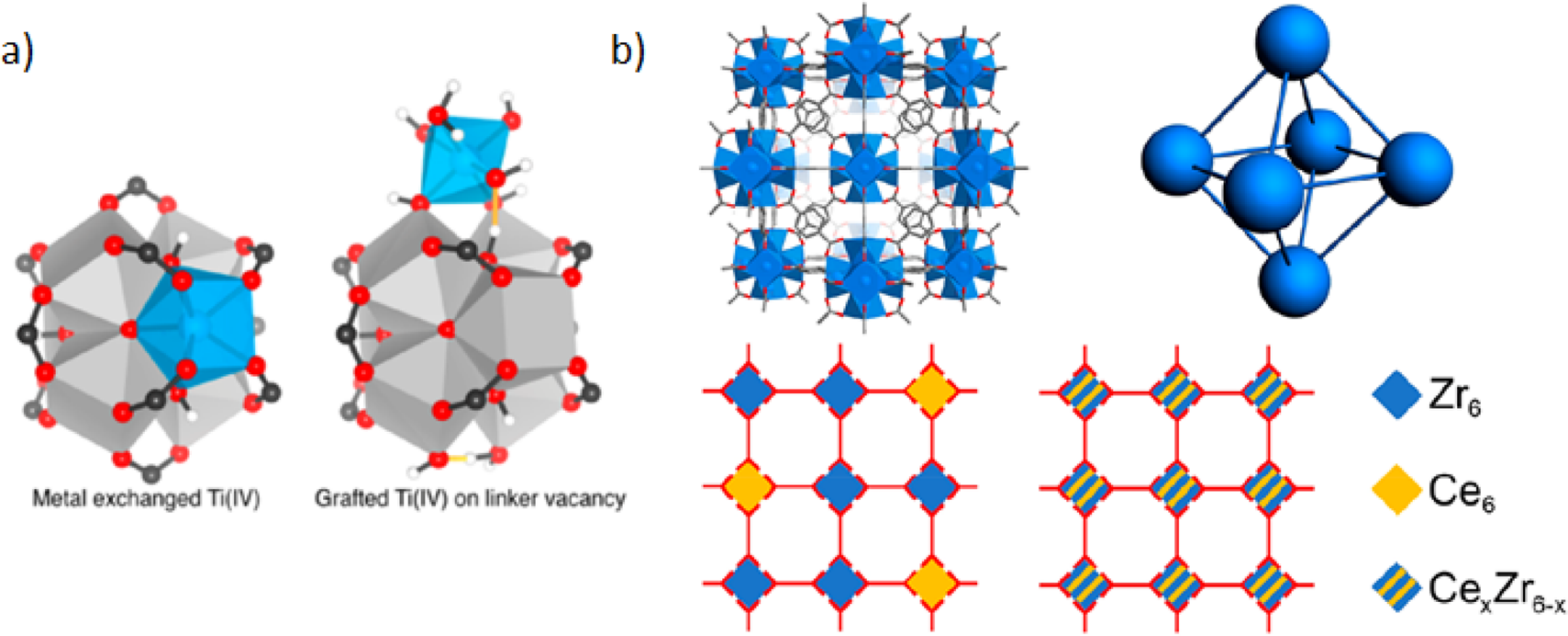
(a) Proposed coordination sites of Ti in UiO-type materials by inclusion of Ti^4+^ in the cluster through metal exchange or attachment of Ti^4+^ to the surface of the cluster at a linker vacancy defect site. Reproduced with permission from ref (267). Copyright 2017 American Chemical Society. (b) Illustration of a fragment of UiO-66 MOF structure, close up on UiO-66 nodes (M atoms only), and possible representation of mixed-metal Ce_*x*_Zr_6–*x*_-UiO-66 structures with single and bimetallic nodes. Reproduced with permission from ref (268). Copyright 2018 American Chemical Society.

One important issue in the preparation of the mixed-metal MOFs is to provide advanced characterization data to firmly discern between real exchange of native metals in the node or attachment of the dopant metal ion at satellite nodal positions. Even if the two metals occupy equivalent nodal positions, several possibilities can still occur, the two metals being either in the same or in different metal nodes. One of the most studied cases of mixed-metal MOF with application in OWS is the preparation of bimetallic mixed-metal UiO-66, including UiO-66(Zr,Ti)^259,263^ or UiO-66(Zr,Ce).^264^ In general, the presence of metal ions with similar ionic radii and affinity to coordinate oxygen atoms^265,266^ should allow their scrambling in the same metal node, although convincing characterization data are still to be provided. In the particular case of UiO-66(Zr/Ti) prepared by postsynthetic exchange, it was initially proposed that Zr^4+^ ions are replaced by Ti^4+^ based on analytical data of the liquid phase and solid material.^259,263^ Latter, it has been proposed that Ti^4+^ ions are more likely accommodated at linker vacancy sites of defective UiO-66(Zr) solid.^267^ In the case of the preparation of bimetallic UiO-66(Zr/Ce) by one-pot synthesis, it is proposed that the most favorable structure is the location of Zr^4+^ and Ce^4+^ ions in independent metal nodes of single metal ions although the formation of a mixed-metal Zr_5_Ce may be possible at Ce contents lower than 17 wt %.^268^Figure 15 illustrates the possible situations that can occur in the mixed-metal UiO-66.

Very recently, the preparation of a trimetallic UiO-66(Zr/Ce/Ti) with enhanced photocatalytic activity for the OWS under visible light irradiation has been reported.^265^ The UiO-66(Zr), UiO-66(Zr,Ce), and UiO-66(Ce) were prepared by one-pot solvothermal methods as previously reported.^265^ The UiO-66(Zr/Ti) and UiO-66(Zr/Ce/Ti) were prepared by starting from UiO-66(Zr) and UiO-66(Zr/Ce), respectively, by postsynthetic modification with a TiCl_4_(THF)_2_ complex. In accordance with previous reports, XRD confirmed that the solids are isostructural to the parent UiO-66(Zr), and the occurrence of partial replacement of Zr^4+^ and Ce^4+^ ions by Ti^4+^ ones were proposed based on ICP analyses of the solid and supernatant solutions after the Ti^4+^ exchange. In particular, XRD showed some shifts of the lowest angle diffraction peak from 7.2° for UiO-66(Ce) to 7.4° for UiO-66(Zr) and UiO-66(Zr,Ce), up to 7.5° for UiO-66/Zr/Ti) and UiO-66(Zr/Ce/Ti). These shifts in the position of the diffraction peak were attributed to the replacement of Zr^4+^ or Ce^4+^ ions by smaller Ti^4+^ ones, causing some contraction of the unit cell. Interestingly, XPS analysis of the multimetallic UiO-66 series supports the presence of mixed-metal nodes of Zr^4+^/Ti^4+^ or Ce^4+^/Ti^4+^ based on the differences in the binding energy values of the Zr^4+^ 3d and Ce^4+^ 3d in comparison with those measured for monometallic UiO-66(Zr) and UiO-66(Ce), in which only Zr_6_ or Ce_6_ nodes are present. These XPS data are in agreement with the computational calculations that indicate that mixed-metal SBU in MOFs are more stable when using metals of similar radii and oxygen affinity.^268,269^

Similarly to the case of UiO-66(Zr), the presence of mixed-metal nodes in MIL-125(Ti/X) (X: V or Nb)^270^ also results in a red-shift of the onset of absorption band that would probably introduce visible-light photoresponse in multimetallic MIL-125, but a detailed photocatalytic study on the photocatalytic activity of mixed-metal MIL-125 is still missing.

### 5.3 Metal Complexes As Guests

**Figure 16:**
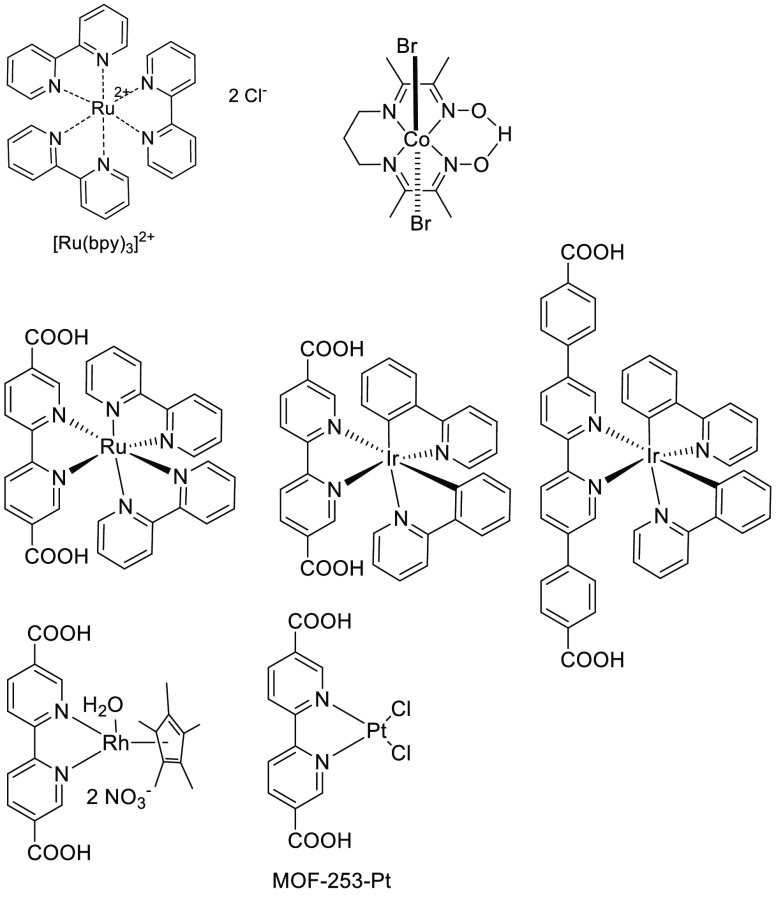
Metal complexes used as visible light harvesting units for the development of visible-light photoactive MOFs.

A widely used approach to harvest visible light and enhance charge separation and, therefore, the photocatalytic activity in MOFs has been incorporation within the MOF pores of transition metal complexes (Figure 16).^142,145,271^ One of the favorite metal complexes as visible light harvester unit in photocatalysis and HER using MOFs have been ruthenium polypyridyl.^141,142,145^ In homogeneous photocatalysis, soluble [Ru(bpy)_3_]^2+^ complex in combination with sacrificial electron donors has been well-studied system for visible light photocatalytic HER.^141,142,145^ This complex has also been incorporated in MOFs, and the resulting Ru-complex-MOFs have been tested in photocatalytic HER. In one example, [Ru_2_(*p*-benzenedicarboxylate)_2_]*_n_* (Figure 16) was employed as photocatalyst together with homogeneous [Ru(bpy)_3_]^2+^ as photosensitizer and methylviologen as electron relay in the presence of EDTA as sacrificial agent for the photocatalytic HER, achieving an AQY of 4.82 at 450 nm.^272^ As an alternative to the use of noble or seminoble metal complexes, a cobalt-oxime (Figure 16a) complex incorporated within the pores of MIL-125(Ti)-NH_2_ following a “ship-in-a-bottle” synthesis exhibits a 20-fold higher activity respect to the parent MIL-125(Ti)-NH_2_ in the photocatalytic HER under visible light irradiation using triethylamine (TEA) as sacrificial agent, CH_3_CN as solvent, and H_2_O as reagent.^273^

In another approach, the metal complex has been anchored to satellite positions of MOF lattice when 2,2′-bipyridine-5,5′-dicarboxylate units can be introduced, at least in some percentage, as linker (Figure 16).^149,272,274−276^ The resulting transition metal complexes act as visible light harvesters promoting efficiently photoinduced electron transfer under visible light. These anchored colored transition metal polypyridyl complexes have been used amply for HER in the presence of sacrificial agents.^277^ For example, a UiO-67(Zr) with a Rh polypyridyl complex is an active with a turnover number (TON) of ∼470 and stable (for 174 h) solid for the visible-light driven photocatalytic HER using H_2_O as solvent and DMF as sacrificial agent.^277^ Similarly to the case of [Ru(bpy)_3_]^2+^, other noble metal complexes have been anchored to the MOF framework and used as visible light photocatalysts for the HER. As an example, iridium complexes incorporated in the framework of a Zr-MOF having within their cavities Pt NPs as cocatalyst resulted in efficient materials (TON up to 1620) for the photocatalytic HER under visible light irradiation (λ > 420 nm) using THF as solvent, H_2_O as proton source, and TEA as sacrificial agent.^149^ In a related study, Pt ions coordinated to the bipyridyl struts of the MOF-253 have been employed as photosensitizer and photocatalyst for the HER in a mixture of CH_3_CN and H_2_O using TEOA as sacrificial electron donor under visible light irradiation, with H_2_ production five times higher than the respective metal complex in solution.^278^

The main limitation of this strategy is the insufficient TONs that can be achieved. Although higher than the parent homogeneous metal complexes in solution, these TONs have still to be increased several orders of magnitude to be competitive with other alternative photocatalytic systems. In addition, for the sake of scalability and affordability and considering industrial applications, it is necessary to develop less synthetically demanding protocols as well as cost-effective photocatalysts based on earth abundant elements.

### 5.4 Porphyrins

**Figure 17:**
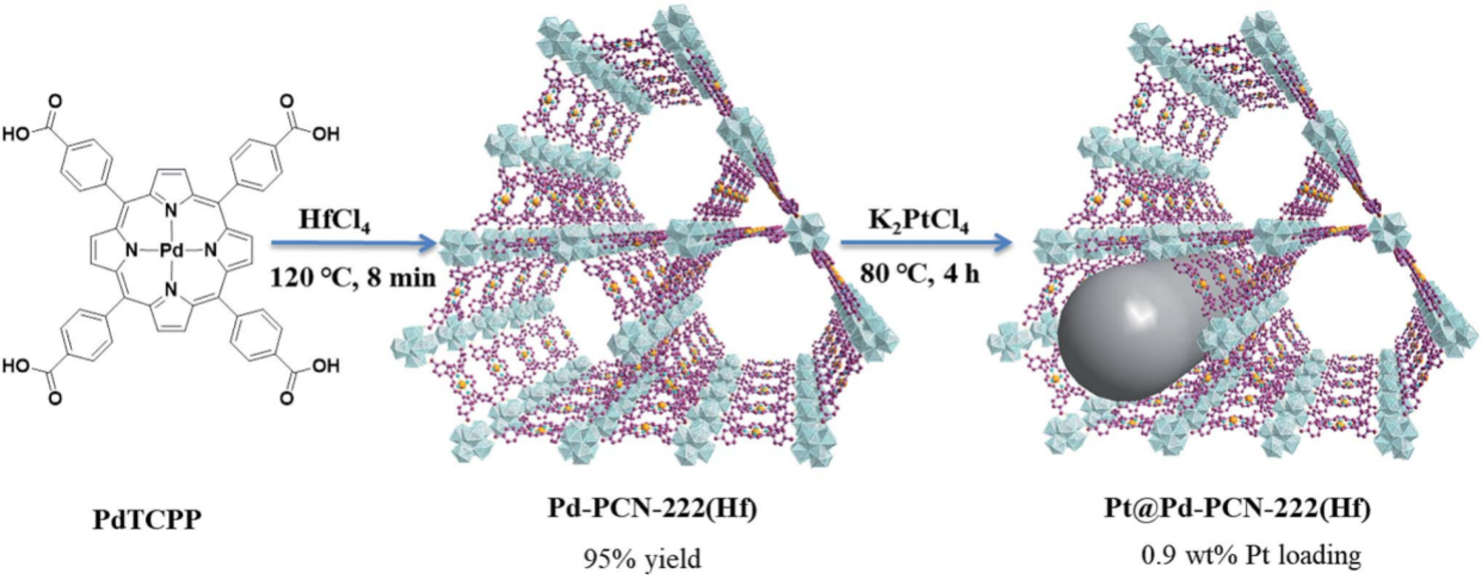
Synthesis of Pd-PCN-222(Hf) and Pt@Pd-PCN-222(Hf). Reproduced with permission from ref (281). Copyright 2019 under noncommercial CC BY-NC 3.0 Royal Society of Chemistry.

MOF-based porphyrins represent a very appealing class of materials to achieve efficient visible light photocatalysts for a variety of applications, particularly for HER in the presence of sacrificial agents.^144,279,280^ As one example, Figure 17 shows the preparation of Pt@Pd-PCN-222(Hf) based on tetrakis(4-carboxyphenyl)porphyrin (TCPP as linker).^281^ Pt@Pd-PCN-222(Hf) is currently one of the most active photocatalysts for the HER (22.67 mmol g^–1^ h^–1^) in CH_3_CN as solvent, H_2_O as proton source, and TEOA as sacrificial agent.

Regarding solar or visible light photocatalytic OWS, the use of porphyrin-based MOF is highly promising due to their tunability and resemblance with natural photosynthetic centers.^144,279^

### 5.5 Other Dye-Sensitized MOFs

**Figure 18:**
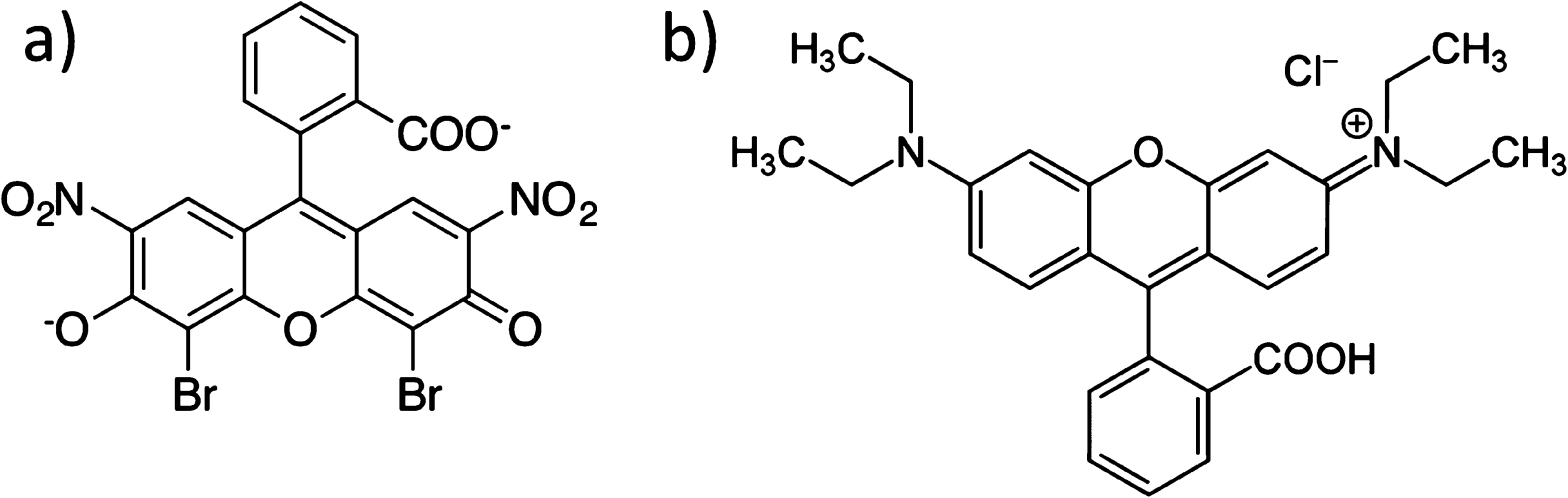
Structures of eosin B (a) and rhodamine B (b).

Analogously to other numerous photocatalysts, dye-sensitization in MOFs is a general strategy to enhance the visible light photocatalytic activity of MOFs.^240,282−284^ More specifically, noncovalent dye-sensitization of MOFs, using dissolved rhodamine B^285^ or eosin B^286,287^ (Figure 18), is a simple and straightforward system to achieve high efficiency for the visible light photocatalytic HER in the presence of sacrificial agents.^284^ MOFs based on aromatic rings can interact with aromatic organic dyes through van der Waals π–π interactions, thus facilitating photoinduced electron transfer from electronic excited state of the dye to the MOF lattice. Coordination bonds can also serve for the purpose of dye immobilization within the MOF pores. One of the common problems, however, when using dye-sensitized photocatalysts, is their low stability at medium-/long-term due to dye degradation.^285,288^ Thus, regardless the efforts made using dye-sensitized MOFs as visible light photocatalysts for H_2_ generation, more detailed studies on the long-term (longer than months) durability should be done as well as suitable strategies to recover the photocatalytic activity of deactivated systems to make this strategy useful.

### 5.6 Cocatalysts to Enhance Efficiency of OWS

Nowadays, cocatalyst deposition on a photocatalyst is a well-established methodology to increase the photocatalytic activity and efficiency.^56,60,289−293,290,292,294^ Cocatalysts favor the photocatalytic reaction by, at least, two general mechanisms, namely, enhancing the efficiency of charge separation, diminishing electron/hole recombination, and by favoring electron transfer to the substrate and gas evolution.^295−299^ Using TiO_2_ as an exemplary case, it has been estimated that charge recombination can be as high as 90% of the initial photogenerated charge carriers.^300^ If a proportion of the photogenerated charges cross a boundary, then, recombination is much disfavored. Transfer of the electrons or holes to the substrate requires a prior substrate adsorption on certain surface sites where the charges are also driven.^293,301,302^ Charge transfer is not an unimportant step and is also based on the large variety of electrocatalysts that are being developed for electrolysis. Electrocatalysis provides inspiration for the development of cocatalysts because there are common elementary steps in both types of catalysts. As in electrocatalysis, in photocatalysis there are cocatalysts for H_2_ generation that are different from those for O_2_ evolution.^303−311^ The final process is gas evolution that may also require promoting the mechanism of H_2_ or O_2_ formation and having a low adsorption energy for these gases.

On the other hand, the cocatalysts decrease the activation energy of OWS semireactions by decreasing the *E*_a_ of the proton reduction and H_2_O oxidation half reactions. It should be mentioned that H_2_O oxidation to O_2_ is thermodynamically and kinetically a more demanding reaction than proton reduction because it requires removal of four electrons and four protons at higher potential to occur.^26,52,56,312^ For these reasons, the development of efficient photocatalysts for the solar-driven OWS requires of simultaneous loading of oxidation and reduction cocatalysts on MOFs. Cocatalysts based on noble metals are among the most active for HER, while OER cocatalysts are typically transition metal oxides. Nevertheless, it is evident that from the industrial point of view, however, the use of catalysts based on nontoxic, cost-effective, and earth-abundant elements is a prerequisite to perform an economically affordable photocatalytic OWS process.^141,291,292,313−317^

These general two roles of cocatalysts have also been assumed to occur in MOFs. On one hand, cocatalysts can promote charge separation due to the low charge carrier mobility of most MOFs. Even for MOFs in which SBUs are 1D metal-oxo chains or 2D sheets in which electron mobility can be faster in one or two dimensions, the OWS process can still be limited by the poor hole mobility. This low charge carrier mobility in MOFs is detrimental for the efficiency of the photocatalytic OWS because a geminate e^–^/h^+^ recombination can be more efficient.

One possible strategy to overcome the poor charge mobility in MOFs is the presence of cocatalysts such as metal NPs, metal complexes, metal oxides, or other derivatives for both the HER and OER. Another role of cocatalysts related with electron transfer to substrates is to facilitate gas formation and evolution by decreasing the activation barrier of the process. In this regard, considering the catalytic activity of open metal sites in MOFs, it can be expected that the importance of this role in MOFs must be much lower than for TiO_2_ and other semiconductors for which their catalytic activity at room temperature is negligible.

From the previous considerations of easier and more efficient charge separation in transition metal complexes and catalytic activity for dark reactions, it is not surprising that cocatalysts in MOFs are apparently less efficient than they are in common metal oxides. One aspect to be considered in the case of MOFs is the appropriate deposition of cocatalysts. Indeed, it is well established that cocatalyst amount, size, and location are crucial factors to reach the highest possible efficiency. In principle, the interface between the semiconductor and the cocatalyst plays a key role in decreasing the resistance for charge migration by alignment of the atomic planes of the two materials.^31,318^ In this context, also in the case of MOFs, depending on the location (internal vs external) of the cocatalyst, the efficiency in boosting the photocatalytic OWS varies, being the optimal when the cocatalyst is internally located.^319^

Although further experimentation is needed to address specifically this issue, it seems from the current state of the art that some of the roles corresponding to the cocatalyst (enhanced charge separation, substrate adsorption, charge transfer to substrate, and gas desorption) can be already played in some extent by the metal nodes. In fact, it is becoming increasingly recognized that MOFs can be efficient electrocatalysts that is a good sign for their suitability as cocatalysts in photocatalysis. The open coordination sites around the metal can bind to water and oxygenated intermediates, leading to O_2_. Addressing the role of cocatalysts in MOFs should be done by a combination of theoretical calculations on models analyzing adsorption values and electron transfer process, photocatalytic evaluation under various conditions, electrochemical measurements, transient absorption spectroscopy studies, and *operando* characterization.

In addition, cocatalysts can diminish the overpotentials for O_2_ and H_2_ formation due to more efficient mechanistic pathway when there are metal nodes. Frequently, H_2_O oxidation to O_2_ requires higher overpotentials than proton reduction to H_2_ at neutral pH values, making the effect of cocatalyst managing photogenerated holes more relevant and important for OWS than those promoting HER. In summary, efficient OWS using MOFs will be achieved when avoiding electron–hole recombination and providing active sites with low overpotential for the total OWS process, and these positive effects can be obtained by coincorporation of cocatalysts.

#### 5.6.1 Cocatalysts for the Photocatalytic HER

**Figure 19:**
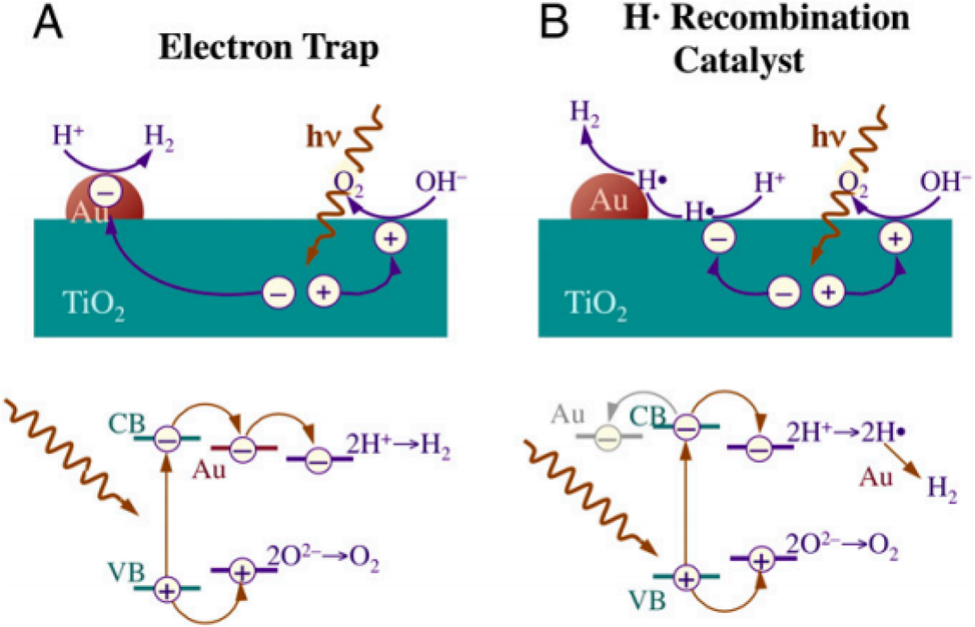
Possible mechanisms (upper) and energetics of the electronic transitions (bottom) proposed to explain the role of Au NPs in the photocatalytic HER for TiO_2_. (A) In the conventional model, the metal acts as an electron trap that physically accumulates conduction band electrons required for proton reduction generating H_2_ on the metal NP, while the oxidation semireaction occurs on the surface of TiO_2_. (B) Alternative proposal for the role of Au NPs in which H^+^ reduction occurs on the semiconductor surface, but the resulting hydrogen atoms undergo recombination to produce H_2_ on the Au surface. Reproduced with permission from ref (321). Copyright 2014 National Academy of Sciences.

Typically, HER is promoted by the presence of cocatalysts based on Pt, Pd, Rh, Au, or Ni NPs. Pt in the form of NPs or clusters is the most widely studied HER cocatalyst to promote the H_2_ evolution due to its high work function and higher effciency.^289,313^ In the photocatalytic HER mechanism, the role of the cocatalyst is to act as an electron trap as well as a catalyst for proton reduction.^320^ More specifically, theoretical calculations have revealed that small Pt clusters supported on TiO_2_ are efficient electron reservoirs with a high work function that favors the catalysis of proton reduction. Besides the nature of the noble metal, particle size and location are important parameters to be considered. Pt NPs of about 1 nm are envisioned as having the optimal size for the HER. This observation is important in the case of MOFs, in which microporous cavities can favor the formation and stabilization of such small NPs. Alternatively to this reaction mechanism widely accepted for Pt NPs, it has been proposed for Au NPs supported on TiO_2_ that photogenerated electrons are trapped directly by TiO_2_ surface adsorbed protons and, then the role of Au NPs is to act as catalysts to promote the atom recombination to molecular H_2_ as illustrated in Figure 19.^321^

Alternatively to the use of noble metals such as Pt or Pd NPs, several studies have reported the potential use of non-noble, cost-effective, and earth-abundant elements as cocatalysts.^141,291,313−317^ For this reason, nickel-based cocatalysts and metallic complexes are expected to gain importance.^299,314,315^ As one example of the use of small-size Ni NPs as cocatalysts, Ni NPs exposing 111 facets supported on MOF-5 was reported as a cost-effective alternative to the use of Pt NPs for the visible-light driven photocatalytic HER using eosin as photosensitizer and TEOA as sacrificial agent. Specifically, the overpotential required for proton reduction to H_2_ decreases from −0.44 to −0.35 V as the Ni particle size decreases from 20 to 3 nm, respectively. Besides particle size, preferential crystal orientation is also a parameter to be considered regarding the activity as cocatalyst. Thus, oriented Ni(111) NP/MOF-5 exhibits an enhanced electron transfer from the MOF to the Ni NPs in comparison to the use of analogous Ni(200)/MOF-5, as revealed by electrochemical impedance spectroscopy.

**Figure 20:**
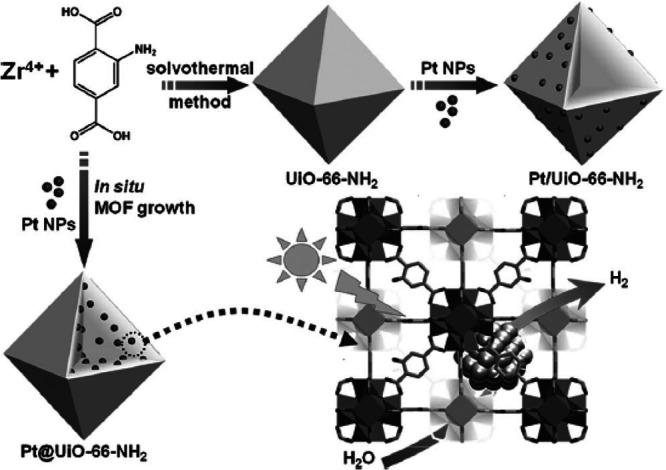
Illustration for the synthesis of Pt@UiO-66(Zr)-NH_2_ and Pt/UiO-66(Zr)-NH_2_, with the photocatalytic H_2_ production process over Pt@UiO-66(Zr)-NH_2_ being highlighted. Reproduced with permission from ref (319). Copyright 2016 Wiley.

Also, differences in the photocatalytic efficiency depending on the external or internal location of the cocatalysts have been reported.^319^ In one study, the use of Pt NPs (∼3 nm) encapsulated within the cavities of UiO-66(Zr)-NH_2_ resulted in much higher photocatalytic activity (∼275 μmol g^–1^ h^–1^) for H_2_ generation in CH_3_CN using TEOA as sacrificial agent under visible light irradiation compared to the situation in which the Pt NPs are supported on the external surface of UiO-66(Zr)-NH_2_ (∼25 μmol g h^–1^) (Figure 20).^319^ Based on photocurrent measurements, photoluminescence spectroscopy, and electrochemical impedance spectroscopy, a more efficient photoinduced electron-pair separation when the Pt NPs are located within the cavities of UiO-66(Zr)-NH_2_ respect to the situation in which the Pt NPs decorate the surface of the UiO-66(Zr)-NH_2_ was proposed. Importantly, ESR measurements revealed the generation of Zr^3+^ in the UiO-66(Zr)-NH_2_ nodal metal cluster due to the occurrence of photoinduced charge separation. The use of ultrafast transient spectroscopy and time-resolved photoluminescence further support the more efficient charge separation when UiO-66(Zr)-NH_2_ has the Pt NPs within the cavities. This study provides fundamental insights about the importance of cocatalyst location within the MOF cavities for the development of improved photocatalysts.

Besides metal NPs, certain metal sulfides have also attracted the interest as alternative non-noble metal cocatalysts for the HER.^322^ In particular, Cd-based sulfides are well-known photocatalysts for H_2_ generation under visible light irradiation. Currently, new research efforts are being done for the replacement of toxic Cd or Pb sulfides by other less-toxic metal elements. One major challenge to be overcome for the development of metal sulfides cocatalysts is corrosion. Regardless, the excellent performance reported in some studies, more efforts should still be done for the replacement of these toxic Cd-based cocatalysts for other more sustainable based on less-toxic and earth-abundant elements. In this regard, bimetallic metal sulfides based on Cu are very promising because they appear to be efficient HER electrocatalysts. In fact, HER electrocatalysis is a field with a high level of maturity that can serve for additional inspiration for the development of HER cocatalysts.

#### 5.6.2 Cocatalysts for the Photocatalytic OER

The most efficient OER cocatalysts are also based on noble or semimetals such as RuO_2_ or IrO_2_.^289,323^ However, base transition metal oxides, and particularly CoO_*x*_^324^ or Co_3_(PO_4_)_2_ (CoPi),^324−326^ can also be employed for this purpose.^289^ As an example, Co_3_O_4_ supported on MIL-101(Cr) has been used as cocatalyst for the photocatalytic OER under visible light irradiation using [Ru(bpy)_3_]^2+^ (bpy: 2,2′-bipyridyl) as photosensitizer and Na_2_S_2_O_8_ as sacrificial electron acceptor.^327^ In this case, the Co_3_O_4_/MIL-101(Cr) exhibited a similar overpotential (0.49 V) than Co_3_O_4_ (0.48 V),^328^ that is, however, still much higher compared with iridium oxide (0.25 V).^329^ This work proposes that MIL-101(Cr) not only acts as matrix to encapsulate Co_3_O_4_ NPs, but MIL-101(Cr) also favors the photoinduced charge transfer from [Ru(bpy)_3_]^2+^ in its excited state and the encapsulated cocatalyst.

**Figure 21:**
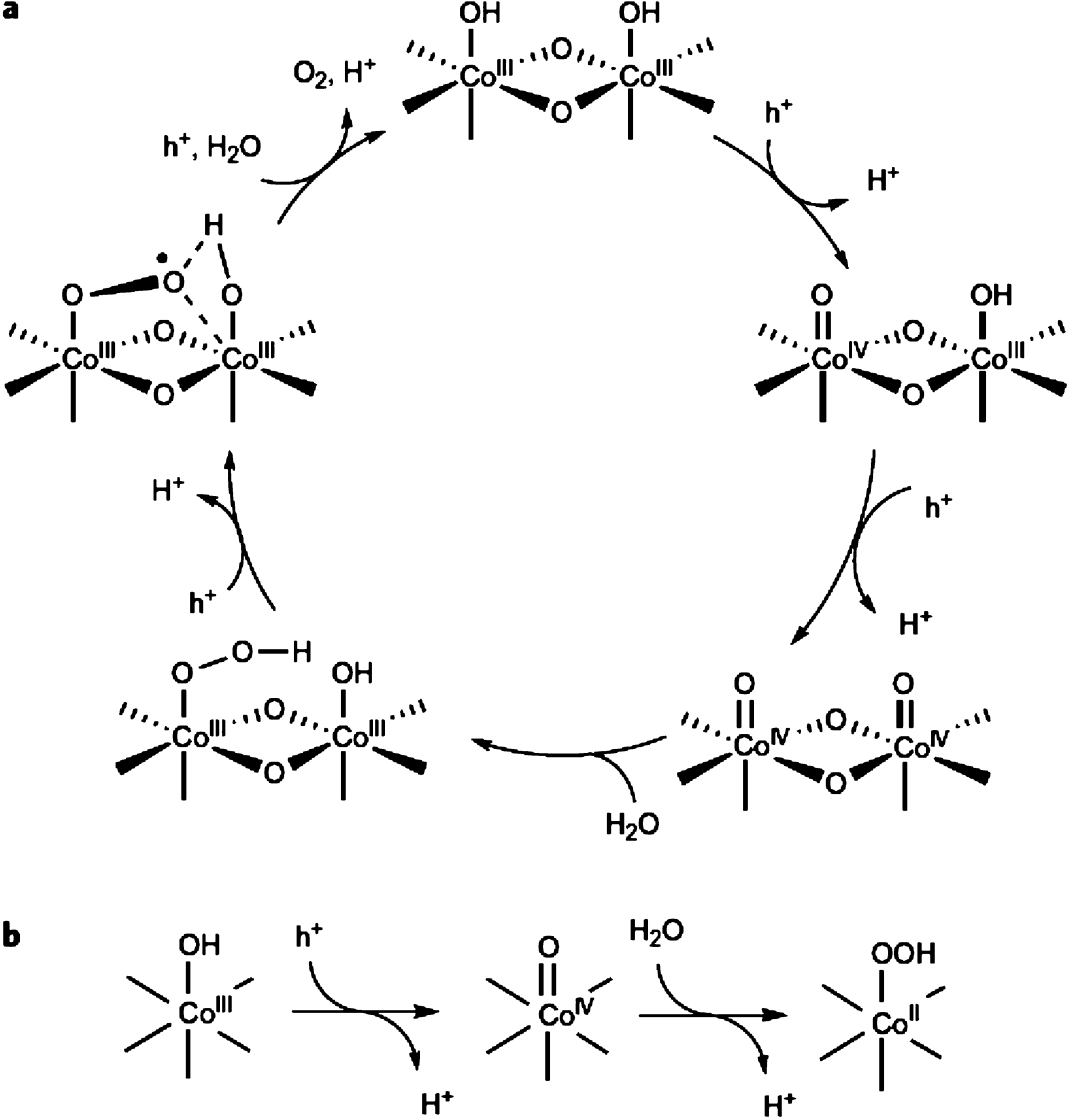
Proposed photocatalytic mechanism for water oxidation on the fast (a) or the slow (b) Co_3_O_4_ surface site. The O–O bond-forming step in the fast cycle features the cooperative effect of two adjacent electronically coupled Co(IV)=O sites, while this cooperative effect is absent in the H_2_O addition reaction at the slow isolated Co site. Reproduced with permission from ref (330). Copyright 2014 Springer Nature.

In a related study, time-resolved rapid scan FT-IR spectroscopy using labeled H_2_^18^O water was used to understand the reaction mechanism of water oxidation using unsupported Co_3_O_4_ NPs (4 nm) as cocatalyst in combination with [Ru(bpy)3]^2+^ as photosensitizer and Na_2_S_2_O_8_ as electron acceptor.^330^ The reaction mechanism was initiated by two sequential hole injections from [Ru(bpy)_3_]^2+^ to Co_3_O_4_ NPs and simultaneous deprotonation to oxidize two adjacent Co(III)-OH groups into Co(IV)=O. A plausible intermediate resonance structure between Co(IV)=O and Co(III)–O^•^ has been proposed (Figure 21). Subsequently, the reaction mechanism occurs in the dark, taking place a nucleophilic addition of H_2_O ending finally as O_2_. With these precedents, it is expected that similar studies would allow elucidation of any active role of MOFs besides as solid matrices defining a compartmentalized space and to find suitable OER cocatalysts that can be used in the photocatalytic OWS process.

In a related study, deposition of transition metal oxides such as MnO_2_, Fe_2_O_3_, Co_3_O_4_, NiO, and CuO within the MOF cavities enhanced the charge separation, increased the photocurrent density, and increased the visible light absorption, favoring the efficiency of independent HER and OER in comparison with pristine MIL-125(Ti).^331^

#### 5.6.3 Cocatalysts Considerations during the Photocatalytic OWS

**Figure 22:**
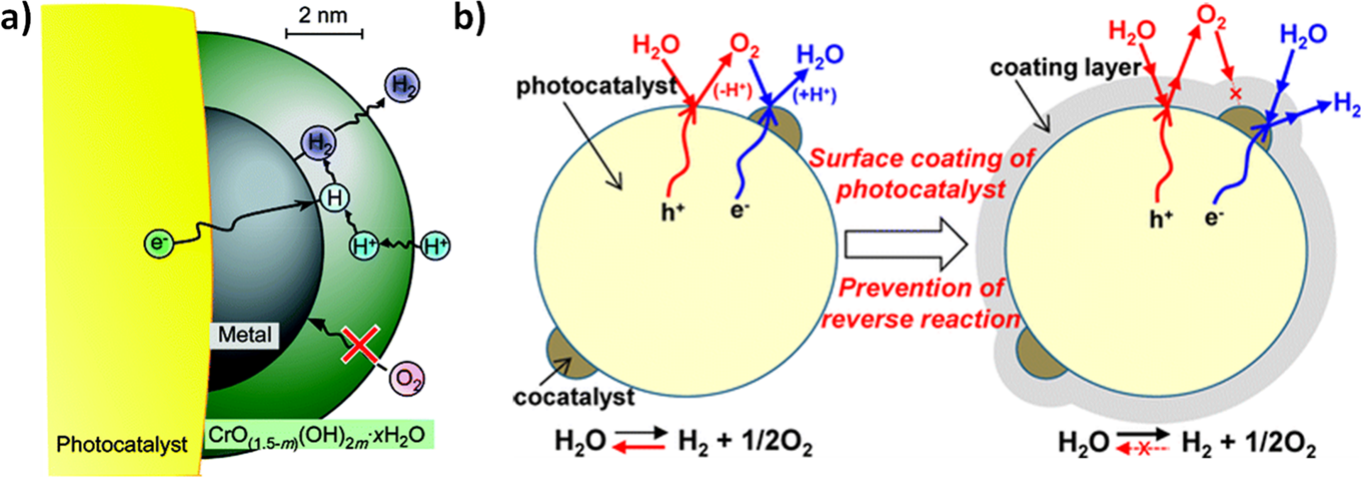
(a) Role of Cr_2_O_3_ layer on noble metal cocatalysts to inhibit OWS back reaction. Reproduced with permission from ref (332). Copyright 2009 American Chemical Society. (b) Coating of photo- and cocatalyst with an amorphous oxyhydroxide overlayer to impede OWS back reaction. Reproduced with permission from ref (333). Copyright 2015 American Chemical Society.

One important point that should be considered when using cocatalysts for the OWS is that they can also catalyze the undesirable water formation from H_2_ and O_2_. This situation can be especially important when using noble metals as HER cocatalysts, and it could be responsible for reaching stationary H_2_ and O_2_ concentrations at long irradiation times. In a series of pioneering studies,^332^ the use of cocatalysts with core–shell structure in which noble metals such as Pt or Rh are the core and a protective Cr_2_O_3_ layer, as shell was found to inhibit the OWS back reaction (Figure 22a). The activity of this core (Pt or Rh)/shell (Cr_2_O_3_) promoting only water splitting but no water formation is because Cr_2_O_3_ is permeable to protons and H_2_ molecules, but not to O_2_. Similarly, photodeposition of amorphous oxyhydroxides of the groups IV and V of transition metals (Ti, Zr, V, Nb) around the entire photocatalytic system (including the photocatalyst and cocatalyst) was found to prevent the OWS back reaction (Figure 22b).^333^

Other important aspect that determines the photocatalytic efficiency during the OWS is the location of the cocatalyst on the photocatalyst. Thus, reduction and oxidation cocatalysts should be in the proper places with respect to the reduction and oxidation sites, respectively. This selective location can be achieved in a simpler manner by using the so-called photodeposition method.^334^ It should be noted that the deposition of the cocatalysts by other common methods employed in catalysis including impregnation or double-solvent method, among others, would result in a random distribution or location of the cocatalysts at places different from those where electrons and holes will react with the semiconductor surface. This misplacement may be detrimental to properly manage reduction and oxidation processes and from the charge carrier recombination point of view.

In summary, all of the available information on cocatalyst efficiency enhancement^335−339^ point on the importance of the proper selection of the cocatalyst, particle size, location, and facet orientation for an optimal activity.

### 5.7 Size and Shape of the MOF Particles

As generally observed with inorganic semiconductors,^37,294,340^ MOF size, shape, and exposed crystal facets may determine their activity as photocatalysts in different reactions,^219,341,342^ including water splitting reaction.^340,343^ In this regard, one common parameter that determines the photocatalytic activity of inorganic photocatalysts is their particle size. One consequence of reducing the particle size in the micrometer or submicrometer scales of a photocatalyst is light penetration on thick particulate films. Due to differences in light scattering as a wavelength function, shorter UV radiations penetrate in a particulate bed less than longer IR wavelengths.

In the use of MOFs as photocatalysts, there are several examples showing the increase of MOF photoactivity as the particle size decreases, and this enhancement has been attributed to better light penetration within the nanometric powdered particles. For example, the photocatalytic activity of nanosized MIL-125(Ti)-NH_2_ solid is higher with respect to the analogous sample having micrometric size crystals.^344^ However, other parameters may also contribute to this enhancement because as-synthesized MIL-125(Ti)-NH_2_ nanocrystals have also abundant structural defects such as oxygen vacancies. These structural defects are more abundant in nanosized materials compared to micrometric crystals due among other things to the presence of additives to arrest particle growth. Other factors to be considered is the easier mass transfer that may also contribute to the higher photocatalytic efficiency of the nanomaterials, as for instance in the photocatalytic benzylamine oxidation reaction.^344^

In one example related to nanometric dimensionality, it has been shown that the photocatalytic activity of 2D UiO-67(Hf) is 84 times higher than its 3D analogue for the HER using MeOH as sacrificial agent under UV–vis irradiation in the presence of H_2_PtCl_6_.^261^ This considerable improvement of photocatalytic activity has been attributed to the occurrence of a more efficient photoinduced electron transfer in the presence of H_2_PtCl_6_ when using the 2D UiO-67(Hf) photocatalyst of nanometric thickness and micrometric lateral size similar to its 3D analogue, as evidenced by fluorescence quenching measurements. Therefore, the better photocatalytic performance of 2D vs 3D UiO-67(Hf) arises from dimensionality and nanometric thickness, even though the lateral size and footprint of the 2D and 3D particles are similar.

**Figure 23:**
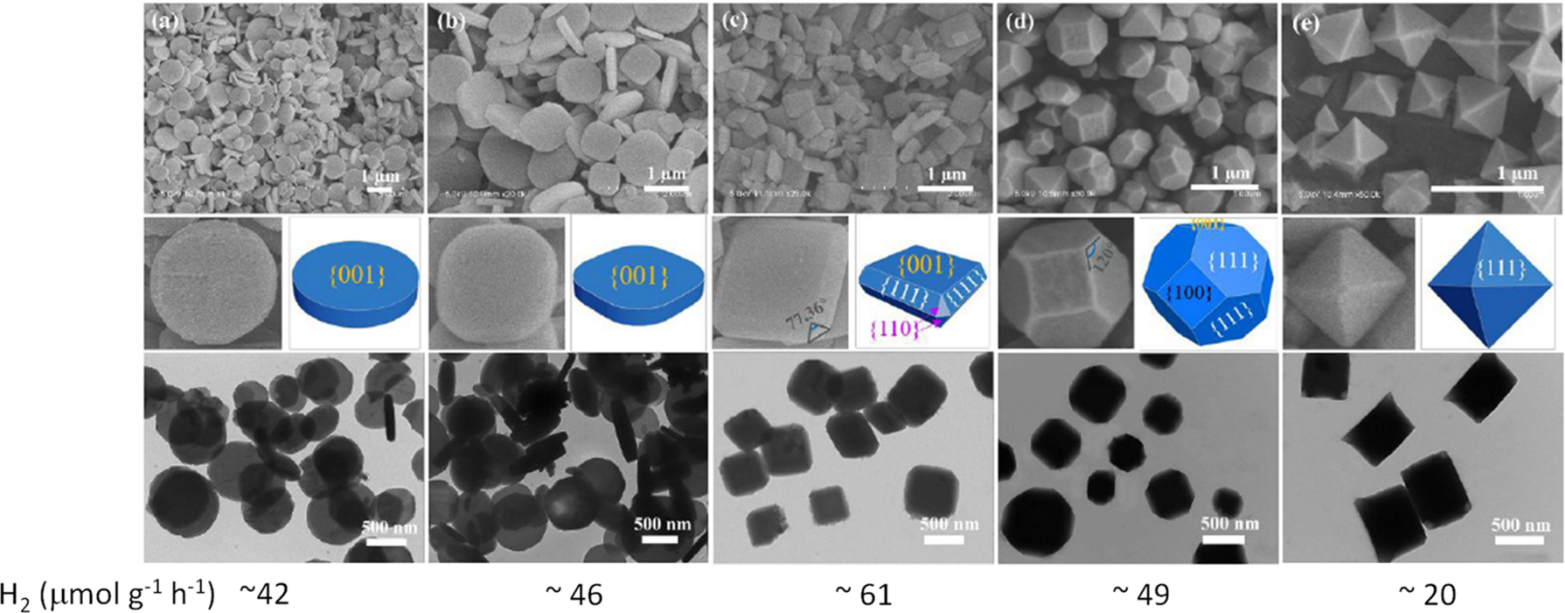
SEM, enlarged SEM, TEM images, and the corresponding 3D geometry models of as-synthesized MIL-125(Ti)-NH_2_, prepared in the presence of different concentrations of CTAB: (a) 0, (b) 1 mM, (c) 2 mM, (d) 3 mM, and (e) 4 mM. The hydrogen evolution values obtained using these solids as photocatalysts (20 mg) suspended in a mixture of CH_3_CN/H_2_O mixture in the presence of TEOA under visible light irradiation (>400 nm) are indicated. Reproduced with permission from ref (345). Copyright 2019 under noncommercial CC BY-NC 3.0, Royal Society of Chemistry.

In an interesting study, the facet-dependence of MIL-125(Ti)-NH_2_ on the photocatalytic activity for H_2_ generation under visible light irradiation using TEOA as sacrificial electron donor was demonstrated.^345^ The preparation of a series of MIL-125(Ti)-NH_2_ with different exposed facets was carried out by changing the concentration of cetyltrimethylammonium bromide (CTAB) during the synthesis. The various particle morphologies are shown in Figure 23. It was found that the sample with truncated tetragonal-like plates containing preferential (110) facets exhibited the highest photocatalytic activity. Based on DFT calculations, it was calculated that this sample should exhibit also the highest surface energy (1.18 J/m^2^). Photoluminescence and electrochemical impedance spectroscopy together with photocurrent measurements confirmed that the MIL-125(Ti)-NH_2_ samples having exposed (110) facets present the most efficient photoinduced electron transfer. This is one of the first examples on MOF facet engineering for the development of materials with enhanced photocatalytic activity, opening another tool to further enhancement of photocatalytic activity.

The textural properties of a photocatalyst also determine its photocatalytic activity. In one example, a Ru-coordinated MIL-125(Ti)-NH_2_ with a hierarchically meso- and microporous structure prepared by a supercritical fluid route exhibited superior photocatalytic activity (426 μmol h^–1^ g^–1^) for visible-light HER in the presence of TEOA compared with an analogous solid constituted by Ru NPs supported on pristine MIL-125(Ti)-NH_2_ (88 μmol h^–1^ g^–1^).^346^ On one hand, this activity enhancement was attributed based on EXAFs and XANES data to the coordination of Ru^3+^ atoms to uncoordinated N/O atoms of the preformed MIL-125(Ti)-NH_2_, resulting in a band gap decrease. On the other hand, the high mesoporosity of the resulting Ru^3+^-MIL-125(Ti)-NH_2_ caused by the etching at 200 °C by the acid derived from CO_2_ carbonation in water generates in the particles a hollow structure as revealed by the TEM images, favoring the exposition of the active sites during the photocatalytic reaction.

In the current state of the art, the influence of the morphology, preferential facet exposure, and particle size on the photocatalytic OWS is still underexplored, and it would be important to understand better the effects of defects, diffusion, and crystal size on the activity.

### 5.8 MOF Heterojunctions

One common strategy in heterogeneous photocatalysis to enhance activity in a material is the preparation of heterojunctions by combining two different photocatalysts. The reader is referred to existing reviews on the activity of heterojunctions of inorganic semiconductors, addressing several aspects including the charge carrier migration due to the various possible band alignments between the components and photocatalytic reaction mechanism.^347,348^ In the particular case of MOFs, a series of studies have combined metal oxide semiconductors such as TiO_2_ with MOFs to enhance the efficiency of the photocatalytic system toward HER under visible light irradiation.^349^ Composites of TiO_2_ and MOFs have been already reviewed, and the reader is referred to this review for deeper understanding.^350^

**Figure 24:**
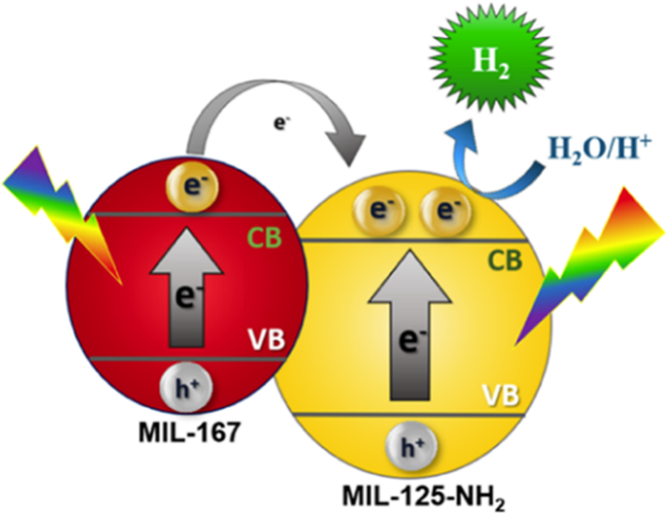
Schematic representation of a type II heterojunction between MIL-167 and MIL-125-NH_2_ operating as photocatalyst for the HER. Reproduced with permission from ref (354). Copyright 2021 American Chemical Society.

Besides combining with TiO_2_, several studies have also shown the possibility of preparing MOF heterostructures combining different MOFs such as MIL-101/UiO-66^351^ and ZIF-8/MIL-125(Ti)-NH_2_.^352^ Taking advantage of these possibilities, a still scarce number of studies have reported the preparation of MOF-on-MOF heterojunctions with application as photocatalysts for the HER in the presence of sacrificial agents.^353^ Recently, it has been reported that the combination of two Ti-based MOF results in a MOF-on-MOF heterojunction with superior photocatalytic activity (455 μmol g^–1^ h^–1^) respect to their individual components, namely MIL-167(Ti) (0.8 μmol g^–1^ h^–1^) and MIL-125(Ti)-NH_2_ (51.2 μmol g^–1^ h^–1^) for the HER in CH_3_CN/H_2_O mixture using TEA as sacrificial agent under visible light irradiation (λ > 420 nm).^354^ The enhancement of photocatalytic activity using the MOF-on-MOF was attributed to the operation of a type II heterojunction mechanism as illustrated in Figure 24. Specifically, the heterojunction exhibits enhanced visible light absorption and charge separation that favors the photocatalytic HER in comparison to their individual components. This study opens new research for the development of MOF heterostructures having, for example, electron donor and electron acceptor domains in separate crystals or even within the same crystal that will favor the photoinduced charge separation and, thus, it could result in enhanced MOF activity for the solar-driven photocatalytic OWS.

As in the case of inorganic semiconductors, it is expected that future studies will report the use of p–n junctions or heterojunctions, phase junctions, and special nanostructures to enhance charge separation in MOF crystals that can finally improve the efficiency of the photocatalytic OWS.

Following the lead of heterogeneous catalysis in which structural defects play an important role, resulting in the generation of Lewis acid sites, defect engineering has also become a common strategy to increase the photoactivity of MOFs.^355^ In particular, several studies have optimized the number of defects in MOFs to increase their activity for the visible-light driven HER^356^ and, importantly, also for the photocatalytic OWS.^357^ To understand the effect, it was reported by theoretical calculations that the presence of missing linkers as defects in the UiO-66(Zr) solid is an appropriate strategy to favor the occurrence of photoinduced electron transfer from the organic ligand to the metal node.^358^ It is worth commenting that in inorganic semiconductors, and particularly TiO_2_, it is well established that a large proportion of defects may play a negative role and can be highly detrimental for their photocatalytic activity.

## 6 Photocatalytic HER under Visible Light Irradiation

Without the aim of being exhaustive due to the extensive existing literature, this section summarizes some of the most active MOFs reported for the photocatalytic H_2_ generation using visible light irradiation in the presence of sacrificial electron donors.^48,119,160,227,229,238,244,247,359−364^ Some pioneering studies for the photocatalytic HER under UV–vis light irradiation that have been also been discussed are included in the section as important precedents related to OWS.

In this regard, it has to be commented that sacrificial electron donors are not innocent reagents, and they can participate in the photocatalytic reaction in several ways besides giving one electron to the photogenerated hole.^365−367^ These additional roles have been particularly studied for TEOA in the case of photocatalytic CO_2_ reduction in which this tertiary amine after giving one electron forms a aminyl radical cation that transfers one hydride to CO_2_ reducing it to formic acid, as it has been studied in detail experimentally and theoretically in the case of Zr porphyrin MOF PCN-222.^365^ TEOA can also react with the carbonyl ligands of Ru bipyridyl complexes in CO_2_ reduction.^366^ But by focusing on H_2_ evolution, it has to be reminded that both amines and alcohols react with holes evolving H_2_ (photoreforming).^368^ In this way, in the presence of these sacrificial electron donors, it is worth to recall that not all the hydrogen evolved derives from conduction band electrons, but only one-half, because equivalent amounts are formed by hole quenching.^368^

### 6.1 UiO-66(Zr) Based Materials

One of the preferred MOF families for photocatalytic HER and OWS is the UiO-66. The main reason for this preference is their large structural, thermal, and chemical stability, high surface area and pore volume, large pore dimensions, affordability of the organic linker, and metal precursor and their easy synthesis. Although reliable synthetic procedures for the preparation of UiO-66 have been reported, there are notable differences in crystallinity and defect density depending on the exact protocol. In 2010, García and co-workers showed for the first time the use of UiO-66(Zr) and UiO-66(Zr)-NH_2_ as photocatalysts for H_2_ evolution in a water/methanol mixture, reporting for the UiO-66(Zr)-NH_2_ containing Pt NPs an AQY of 3.5% at 370 nm.^50^ Under these conditions, it can be expected that H_2_ derives both from H_2_O reduction but also from methanol photoreforming (consumption of holes by methanol). The authors confirmed that the presence of the −NH_2_ group in the UiO-66(Zr)-NH_2_ expands the light absorption beyond 400 nm with respect to the parent isostructural UiO-66(Zr) solid with absorption up to about 310 nm. This shift in the onset of light absorption to the red for UiO-66(Zr)-NH_2_ results in an increased photocatalytic activity respect to the pristine UiO-66(Zr) solid. Furthermore, the presence of preformed colloidal Pt NPs in the system increases somewhat the photocatalytic H_2_ generation. Using UiO-66(Zr)-NH_2_ as photocatalyst, laser-flash photolysis measurements and quenching experiments revealed the presence of long-lived species (>300 μs) upon excitation at 355 nm and the photogeneration of electrons/hole pairs, two important prerequisites to achieve high photocatalytic activity. This study has been considered as a seminal work in the field of photocatalytic H_2_ productions by MOFs because it proved for the first time the ability of MOFs to act as photocatalysts for HER.

Since then, the number of studies reporting the use of UiO-66(Zr) based materials rapidly increased. In a series of studies, UiO-66-based materials incorporating Cd-based semiconductors,^369^ such as CdS^361,369^ or Cd_0.2_Zn_0.8_S,^210^ were prepared and used as photocatalysts for H_2_ generation in the presence of sacrificial electron donors. It was found that Cd_0.2_Zn_0.8_S@UiO-66-NH_2_ reaches a H_2_ generation of 5.85 mmol g^–1^ h^–1^ under visible light irradiation in water in the presence of Na_2_S (0.1 M) and Na_2_SO_3_ (0.1 M) as sacrificial electron donors.^210^ Regardless the relative good H_2_ productions achieved in some cases, the use of toxic Cd metal hampers the large scale applicability of these photocatalytic systems.^370^

As previously commented, defect engineering has been proposed as a useful tool to tune the physicochemical and electronic properties of the solids,^358,371^ resulting in the enhancement of the photocatalytic activity in different reactions.^357,358^ Defect engineering has been particularly studied for the UiO-66 MOF by preparing different samples by using synthetic protocols employing organic or inorganic modulators and also by postsynthetic defect generation.^372−375^ One of these studies has shown the influence of defect engineering on UiO-66(Zr)-NH_2_ activity by synthesizing this material in the presence of several equivalents of acetic acid as modulator with respect to the 2-aminoterephthalate ligand, measuring the photocatalytic activity for H_2_ generation in CH_3_CN/H_2_O mixture and TEOA as sacrificial agent under UV–vis irradiation in the presence of supported Pt NPs (1 wt %, 1.2–1.4 nm) as cocatalysts.^356^ A volcano plot of photocatalytic activity vs defects was found with the lowest activity corresponding to the UiO-66(Zr) sample prepared without modulator. The optimal sample corresponded to the one synthesized using 100 equiv of acetic acid. Beyond this acetic acid proportion, the photocatalytic activity again decreases. Time-resolved transient absorption spectroscopy revealed that the faster the average relaxation lifetime of the photogenerated transients, the higher the photocatalytic efficiency for H_2_ generation (190 μmol g^–1^ h^–1^). In good agreement, photocurrent measurements and electrochemical impedance spectroscopy led to the conclusion that a moderate number of defects may cause a decrease in the energy of the unoccupied d orbitals of Zr^4+^ ions, with a beneficial influence for separation and transfer of photogenerated charge. In contrast, an excess of defects UiO-66(Zr)-NH_2_ prepared with higher than 100 equiv of acetic acid favors charge recombination of photogenerated charge, leading to a decrease of the photocatalytic activity for H_2_ generation.

Heterometallic MOFs^376,377^ and related materials^378^ can exhibit a substantial improvement of their photocatalytic activity, including H_2_ generation compared to related monometallic analogues. In a seminal work, Li and co-workers reported the improvement of photocatalytic H_2_ generation using mixed-metal UiO-66(Zr/Ti)-NH_2_ containing Pt NPs (3.5 mmol/mol) with respect to the reference material Pt NPs supported on UiO-66(Zr)-NH_2_ (2.5 mmol/mol) under visible light irradiation (λ > 420 nm) in the presence of TEOA as sacrificial agent.^259^ The UiO-66(Zr/Ti)-NH_2_ was prepared from UiO-66(Zr) by postsynthetic exchange of Zr by Ti. Theoretical calculations revealed that the introduction of Ti atoms should contribute significantly to the bottom of the LUCO of the materials and, thus, this energy level facilitates the electron transfer from the excited 2-aminoterephthalate to the Ti moiety, leading to the formation of (Ti^3+^/Zr^4+^)_6_O_4_OH)_4_ that can subsequently lead to the generation of Zr^3+^ species. This proposal and the role of Ti as electron relay was supported by ESR measurements. Later, Li, Garcia, and co-workers further confirmed the role of Ti^4+^ as mediator by analyzing the kinetics of a series of UiO-66(Zr/Ti)-NH_2_ with different percentages of exchanged Ti by using transient absorption and photoluminescence spectroscopies.^260^ It should be noted that later some papers argued about the occurrence of a Zr^4+^ by Ti^4+^ ion exchange at the UiO-66 nodes and proposed the attachment of Ti^4+^ ions at satellite nodal positions or at linker defective sites rather than a true Ti^4+^ by Zr^4+^ metal exchange.^267^ However, the exact position of Ti^4+^ may not affect to its role as electron relay from the linker electronic excited state to Zr^4+^.

**Figure 25:**
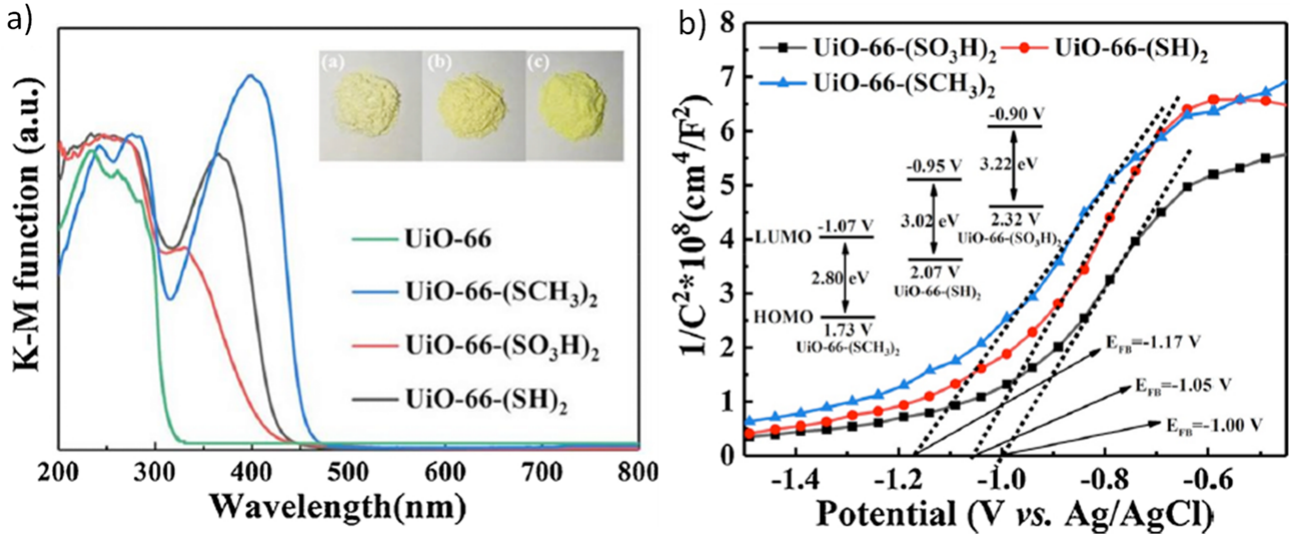
(a) UV–vis absorption spectra of UiO-66 based solids. (b) Mott–Schottky plots of disubstituted UiO-66 with S containing functional groups. Reproduced with permission from ref (379). Copyright 2019 Elsevier.

Besides the use of 2-aminoterephthalic acid for the modification of the parent UiO-66 to enhance visible light absorption, the use of 2,5-(dimethylthio)terephthalic acid also resulted in an efficient visible-light responsive photocatalyst (2.8 eV) for H_2_ generation (∼1.29 mmol g^–1^ h^–1^) using ascorbic acid as sacrificial agent under visible irradiation (λ > 400 nm).^379^ Importantly, the UiO-66(Zr)-(SCH_3_)_2_ exhibited narrower band gap and more negative conduction band than UiO-66(Zr)-(SH)_2_ or UiO-66(Zr)-(SO_3_H)_2_ analogues (Figure 25). These two alterations of the electronic properties are beneficial for the photocatalytic H_2_ generation under visible light irradiation. Importantly, this work has shown that the nature of the terephthalate functional group tunes both the band gap and the HOCO and LUCO energy positions. The stronger the electron donor character, the narrower the band gap and the more negative LUCO value. Both features play a positive role, resulting in higher H_2_ generation in the presence of electron donors.

Improvement of charge separation and minimization of charge recombination are two beneficial factors leading to the performance enhancement of active materials. In this context, a series of studies have reported the enhancement of the photocatalytic activity of UiO-66 for H_2_ generation by preparing composites of this type of MOF with C-dots,^380^ g-C_3_N_4_,^286,380^ or graphene-based materials.^369,381^ In one of these examples, a heterojunction of UiO-66(Zr) with g-C_3_N_4_ was first prepared, and then C-dots were introduced within the pore cavities.^380^ The optimized C-dot(2.77 wt %)@UiO-66(Zr)-NH_2_/g-C_3_N_4_ composite exhibited a photocatalytic H_2_ generation rate of 2.93 mmol g^–1^ h^–1^ under visible-light irradiation. This value is 32.4, 38.6, and 17.5 times higher that g-C_3_N_4_, UiO-66(Zr)-NH_2_ and the composite UiO-66(Zr)-NH_2_/g-C_3_N_4_, respectively. Based on spectroscopic, electrochemical and photocatalytic measurements, it was concluded that the good activity of the ternary composite arises in a large extent from the incorporation of C-dots within the UiO-66(Zr)-NH_2_/g-C_3_N_4_ heterojunction. C-Dots increase the visible light absorption of the composite and extend the lifetime of photoinduced charge separation. To further confirm these conclusions, it would have been convenient to test the photocatalytic activity of the composites constituted by C-dots and UiO-66(Zr)-NH_2_ or C-dots and g-C_3_N_4_ as reference samples. In any case, this work exemplifies the potential of C-dots in the preparation of advanced MOF-based catalysts with enhanced activity for H_2_ generation in the presence of sacrificial agents under visible light irradiation.

**Figure 26:**
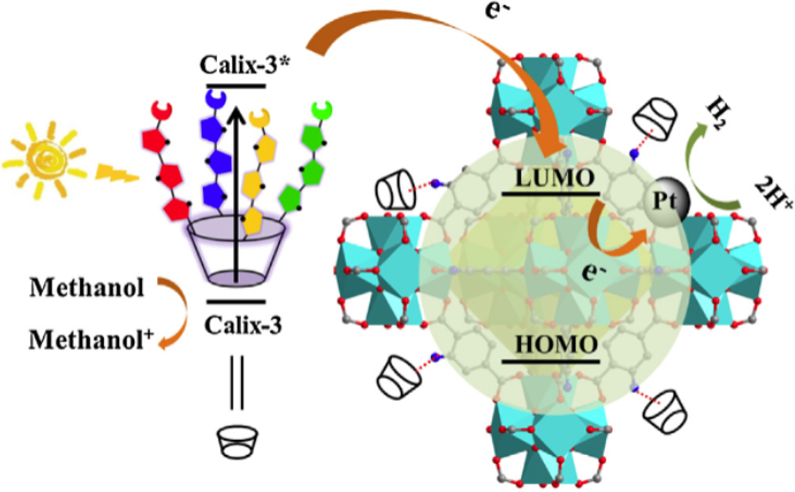
Proposed mechanism of photocatalytic H_2_ production over Calix-3 sensitized Pt@UiO-66-NH_2_ under visible light irradiation. Reproduced with permission from ref (382). Copyright 2017 Elsevier.

Another research direction has used dyes such eosin B,^286^ erythrosine B,^286^ or cone-calix[4]arene dye (Calix-3),^382^ among others, to sensitize MOFs and increase the photoresponse of the resulting photocatalytic system for H_2_ generation in the presence of sacrificial agents and visible light irradiation.^286,381^ The role of dyes appears to be the harvesting of visible photons and upon excitation transferring electrons to the MOF that will be the site for H_2_ evolution. The dye radical cation is generated in the process. The role of the electron donor agent is to restore the radical cation of the dye to the initial ground state (Figure 26). Regardless, the excellent photocatalytic activities achieved in some cases (up to 41.4 mmol g^–1^ h^–1^ using erythrosine_B/UiO-66(Zr)-NH_2_/rGO),^286^ long-term studies under the reaction conditions are required to firmly establish the turnover of these dyes and the potential use of these systems under more realistic conditions.

Table 3 summarizes some of the studies reporting high efficiency on photocatalytic H_2_ generation in the presence of sacrificial electron donor carried out using UiO-66(Zr) as active component, indicating some relevant conditions and H_2_ production.

**Table 3:** Photocatalytic H_2_ Generation Using UiO-66 Materials under Visible Light Irradiation

photocatalyst	co-catalyst	reaction conditions	H_2_ production	general remarks	ref
UiO-66(Zr)-NH_2_	Pt NPs	catalyst (10 mg), solution (18 mL CH_3_CN, 0.2 mL H_2_O), TEOA as sacrificial (2 mL), irradiation source (300 W Xe lamp, λ > 380 nm)	∼0.275 mmol g h^–1^	catalyst and H_2_ production are stable during 4 consecutive photocatalytic cycles for 10 h total time	(319)
UiO-66(Zr/Ti)-NH_2_	Pt NPs	photocatalyst (50 mg), solvent (H_2_O, H_2_O/TEOA (5/1), solution volume 60 mL), irradiation source (300 W Xe lamp, λ > 420 nm)	0.35 mmol g^–1^ h^–1^	Ti acts as electron mediator during the photocatalytic event	(259)
UiO-66(Zr)-(SCH_3_)_2_	Pt NPs	photocatalyst (50 mg), ascorbic acid as sacrificial (0.2 M optimized amount), λ > 400 nm,3 h	∼1.29 mmol g^–1^ h^–1^	photocatalyst and H_2_ generation are stable upon 3 consecutive reuses for 9 h total time	(379)
C-dots@UiO-66(Zr)-NH_2_-g-C_3_N_4_	Pt NPs (0.8 wt %)	photocatalyst (10 mg), aqueous solution (100 mL), sodium ascorbate (5.05 M), pH 6.5, irradiation source (300 W Xe lamp, λ > 420 nm),5 °C	2.93 mmol g^–1^ h^–1^	photocatalyst and H_2_ generation are stable upon 4 consecutive reuses for 20 h total time	(380)
UiO-66(Zr)-NH_2_/rGO(50 wt %)/erythrosine B	Pt NPs (5 wt %)	photocatalyst (5 mg), aqueous solution (30 mL; 3 mL TEOA sacrificial per 1 L H_2_O), dye (50 mg), irradiation source (300 W Xe lamp, λ > 420 nm)	41.4 mmol g^–1^ h^–1^	UiO-66(Zr)-NH_2_ activity without rGO of 21.2 mmol g^–1^ h^–1^ AQE at 420, 450, 500, and 550 nm of 8.8, 5.8, 5.3 and 3.3%, respectively	(286)
UiO-66-NH_2_/GO	MoS_2_(5 wt %)	photocatalyst (30 mg), aqueous solution (100 mL, 10 vol% TEOA, pH 7), MoS_2_ (5 wt %), eosin Y (0.4 mM), irradiation source (300 W Xe lamp, λ > 420 nm)	2.07 mmol g^–1^ h^–1^	photocatalytic activity decreases more than 60% after the first run attributed to dye degradation. AQE at 430, 460, 520, and 550 nm were 40.5, 14.3, 18.1, and 3.59%, respectively.	(383)
UiO-66(50 wt %)/CdS	MoS_2_(1.5 wt %)	photocatalyst (20 mg), aqueous solution (80 mL; 10% lactic acid as sacrificial, irradiation source (300 W Xe lamp, λ > 420 nm)	32.5 mmol g^–1^ h^–1^	photocatalytic activity slightly decreases (ca. 12%) and Cd^2+^ leaching occurs (ca. 2.1%) upon four consecutive uses for 16 h total time	(361)
UiO-66-NH_2_/2D-COF (TpPa-1: 2,4,6- *p*-phenylenediamine)	Pt NPs (3 wt %)	photocatalyst (10 mg), aqueous buffer solution (50 mL, 0.1 M PBS, pH 7, 100 mg sodium ascorbate as sacrificial), 4 °C, (300 W Xe lamp, λ > 420 nm)	23.41 mmol g^–1^ h^–1^	photocatalyst and H_2_ generation are stable during 20 consecutive uses for 20 days total time	(384)

### 6.2 MIL-125(Ti) Based Materials

MIL-125(Ti) structure is composed by octameric titanium oxoclusters coordinated to terephthalate ligands, having an ideal formula [Ti_8_O_8_(OH)_4_(C_6_H_4_C_2_O_4_)_6_].^249^ MIL-125(Ti) has also been one of the favorite MOFs as photocatalyst for hydrogen generation.^90^ Among the various studies reported, MIL-125(Ti)-NH_2_ loaded with Ni_2_P as cocatalyst and using TEA as sacrificial agent exhibited a notable H_2_ production (1.23 mmol g^–1^ h^–1^) and MOF stability, as revealed by XRD after one use.^385^ Alternative electron donor compounds to TEA such as TEOA (0.21 mmol g^–1^ h^–1^), ethanol (0.04 mmol g^–1^ h^–1^), and methanol (0.027 mmol g^–1^ h^–1^) render lower H_2_ production, while no activity was observed using ascorbic acid. This is certainly an interesting observation that is against the general understanding of the role of the electron donors to quench highly oxidizing holes. Accordingly, similar H_2_ production rates should be expected provided that the oxidation potential is met. Attempts to use other cocatalysts such as NiO, Co_3_O_4_, CoP, Fe_2_O_3_, and CuO in the presence of TEA as sacrificial electron donor resulted in lower activities with the values of 1.08, 0.828, 0.828, 0.435, and 0.129 mmol g^–1^ h^–1^, respectively. Clearly, these data, although highly relevant for photocatalyst optimization, are very empirical and require a deeper understanding to be rationalized.

In a related study, the use of 50%-MIL-125-(SCH_3_)_2_/MIL-125(Ti) loaded Pt NPs and TEA as sacrificial agent resulted in photocatalyst decomposition. Attempts in the use of MeOH, EDTA, or aniline as sacrificial agents resulted in no H_2_ production, while the use of vitamin C (66.7 μmol g^–1^ h^–1^) or TEOA (124.3 μmol g^–1^ h^–1^) resulted in low H_2_ generation.

Interestingly, MIL-125(Ti) heterojunctions with g-C_3_N_4_, graphenes,^386^ TiO_2_, or covalent organic frameworks (COFs) exhibit photocatalytic H_2_ generation activity under visible light irradiation in the presence of sacrificial agents. It is proposed that MIL-125(Ti) composites establish an intimate contact with carbon-based materials through π–π interactions, a prerequisite for the design of efficient heterojunctions with improved photoinduced electron transfer. Regardless of the advances in the field, there is still room to improve the efficiencies as well as the stability of the composites.

**Figure 27:**
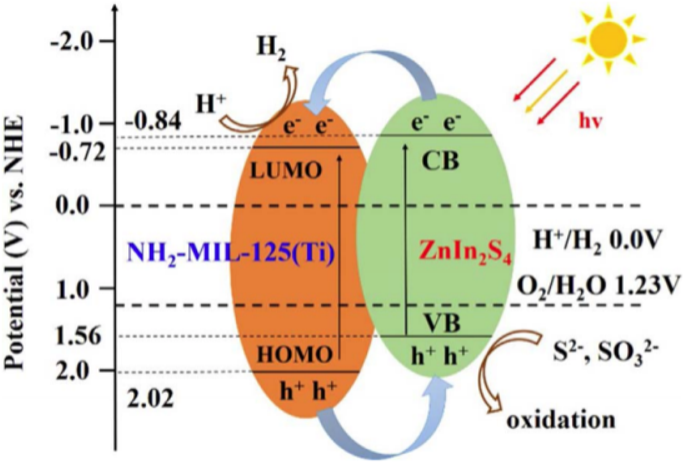
Energy level diagrams showing the band alignment as type II heterojunction in the case of ZnIn_2_S_4_/MIL-125(Ti)-NH_2_. Reproduced with permission from ref (212). Copyright 2018 Elsevier.

Besides carbon materials, other types of semiconductors have also been combined with MIL-125(Ti) to enhance the photocatalytic H_2_ generation efficiency. As an alternative to the use of Cd-based sulphide as hosts and to overcome the toxicity of the Cd^2+^ ions, ZnIn_2_S_4_ within MIL-125(Ti)-NH_2_ resulted in an active and stable heterostructure for H_2_ production under visible light irradiation.^212^ The synergistic photocatalytic activity of the optimized ZnIn_2_S_4_(40%)/MIL-125(Ti)-NH_2_ has been attributed to the appropriate band alignment (Figure 27), and intimate contact of the two components favoring the photoinduced charge carrier separation and migration.

Table 4 summarizes some of the most relevant results for H_2_ production using MIL-125(Ti) MOFs as photocatalysts under visible light irradiation.

**Table 4:** Photocatalytic H_2_ Generation Using MIL-125(Ti) MOFs under Visible Light Irradiation

photocatalyst	cocatalyst (wt%)	reaction conditions	production (mmol g^–1^ h^–1^)	general remarks	ref
MIL-125(Ti)-NH_2_	Ni_2_P (9.2 wt %)	photocatalyst (17 mg), solution (17 mL; 13.4 mL CH_3_CN, 0.8 mL H_2_O and 2.8 mL TEA as sacrificial agent), irradiation source (300 W Xe lamp, λ > 420 nm)	1.23	structural integrity of the photocatalyst after use was maintained as shown by XRD	(385)
50%-MIL-125-(SCH_3_)_2_/MIL-125(Ti)	Pt NPs (1.5 wt %)	photocatalyst (50 mg), aqueous solution (80 mL; 0.01 M TEOA as sacrificial agent), irradiation source (300 W Xe lamp, λ > 420 nm)	3.81	quantum yield at 420 nm of 8.9%; The catalyst decomposed due to the presence of TEA	(387)
MIL-125(Ti)-NH_2_/g-C_3_N_4_(0.75 wt %)	Ni_15.8_P_2.1_	photocatalyst (10 mg), aqueous solution (1% TEOA), eosin (0.5 mM) as photosensitizer, irradiation source (300 W Xe lamp, λ > 420 nm)	8.7	photocatalytic activity decreases more than 60% after the fourth cycle with a total recycling time of 20 h	(388)
MIL-125(Ti)-NH_2_@TiO_2_		photocatalyst (5 mg), solution (5 mL CH_3_CN, 0.2 mL H_2_O, 1 mL TEOA as sacrificial agent), irradiation source (300 W Xe lamp, λ > 380 nm)	∼0.5	MIL-125(Ti)-NH_2_@TiO_2_ is stable for three uses, while MIL-125(Ti)-NH_2_ loses half activity after one use	(389)
MIL-125(Ti)-NH_2_-COF	Pt NPs (3 wt %)	photocatalyst (20 mg), solution (80 mL; H_2_O/TEOA72:8), irradiation source (300 W Xe lamp, λ > 420 nm),6 °C	0.36	quantum yield at 420 nm is 0.87%. The photocatalyst and H_2_ generation are stable upon 4 consecutive reuses for 16 h total time	(390)
Zn_0.5_Cd_0.5_S(40%)/MIL-125-NH_2_(Ti)		photocatalyst (20 mg), aqueous solution with sacrificial agent (10.9 g or 45 mmol Na_2_S·9H_2_O and 100 mmol Na_2_SO_3_), irradiation source (300 W Xe lamp, λ > 400 nm)	92.5	AQE at 420 nm is 30.8%. The photocatalyst and H_2_ generation are stable for 4 consecutive runs with 16 h total reaction time	(391)
ZnIn_2_S_4_(40%)@NH_2_-MIL-125(Ti)		photocatalyst (50 mg), aqueous solution (100 mL; 0.35 Na_2_S and 0.25 M Na_2_SO_3_), irradiation source (300 W Xe lamp, λ > 420 nm), room temperature	2.2	AQE at 420 nm is 4.3%; stable catalyst and H_2_ production during five consecutive uses with a total of 20 h	(212)

### 6.3 MIL-101 Materials

MIL-101 solids have also been employed as photocatalysts for the HER in the presence of sacrificial agents.^248,392^ In one of these reports, particularly high photocatalytic activity, has been reported for H_2_ generation using MIL-101(Cr) solid modified with Cd-based cocatalysts. A remarkable activity (25 mmol g^–1^ h^–1^) has been reported using the Au_core_@CdS_shell_/MIL-101(Cr)(60%) photocatalyst under visible light irradiation. As commented before, regardless of the excellent activities obtained when using Cd-based cocatalysts combined with MOFs, their toxicity hampers real applications. Table 5 summarizes other reports on the visible light photocatalytic H_2_ generation using MIL-101(Cr) MOFs.

**Table 5:** Photocatalytic H_2_ Generation Using MIL-101(Cr) Materials under Visible Light Irradiation

photocatalyst	cocatalyst	reaction conditions	production	general remarks	ref
Au_core_@CdS_shell_/MIL-101(Cr) (60%)		photocatalyst (10 mg), aqueous solution (100 mL; 20 mmol of Na_2_S and Na_2_SO_3_ as sacrificial agents), irradiation source (300 W Xe lamp, λ > 420 nm)	25 mmol g^–1^ h^–1^	AQY at 420 nm is 8.8%, Good reusability and catalyst stability during 4 consecutive runs with a total time of 8 h	(393)
Ti_6_O_4_(OiPr)_10_(O_3_P-Phen)_2_(L)_2_(L = *R*-1,1′-bi-2-naphthol)/MIL-101(Cr)	CdS (6.5 wt %)	photocatalyst (15 mg), aqueous solution (100 mL; 20 mmol of Na_2_S and Na_2_SO_3_ as sacrificial agents), irradiation source (300 W Xe lamp, λ > 420 nm)	∼94.4 mmol g^–1^_CdS h^–1^	photocatalytic activity decreases about 20% after 10 uses for 20 h total reaction time	(394)
CdS(10 wt %)/MIL-101(Cr)	Pt NPs (0.5 wt %)	photocatalyst (20 mg), aqueous solution (10% lactic acid), irradiation source (300 W Xe lamp, λ > 420 nm)	∼7.5 mmol g^–1^ h^–1^	MIL-101(Cr) without CdS does not show photocatalytic activity	(395)

### 6.4 Porphyrin-Based MOFs

Porphyrins are well-known organic macrocycles whose photophysical and photochemical properties have been amply studied. Several MOFs contain porphyrin units as linkers. Table 6 summarizes the performance of some of the most active porphyrin-based MOFs as visible light photocatalysts for H_2_ generation in the presence of sacrificial agents. The reader is referred to a recent review on a historical overview of the development of porphyrin-based MOFs focused on their design, structures, and providing a general view of their applications.^144,279^ As it can be seen in Table 6, these porphyrin-based photocatalysts are in general efficient for the production of H_2_ when combined with Pt NPs as cocatalysts. Especially high activity has been found using a Pd-PCN-222(Hf) combined with Pt NPs reaching a remarkable H_2_ production as high as 23 mmol g^–1^ h^–1^. Considering the flexibility in the preparation of MOFs containing porphyrins, it is expected that this field will grow considerably in the next years. Particular efforts should be performed to harvest the entire solar spectrum, with the aim to further increase the solar energy-to-hydrogen AQYs.^396^ Provided that photochemical stability is firmly demonstrated, development of scalable and cost-effective porphyrin MOFs would be an important step forward for their potential industrial application.

**Table 6:** Photocatalytic H_2_ Generation Using Porphyrin-Based MOFs under Visible Light Irradiation

photocatalyst	cocatalyst	reaction conditions	H_2_ production (mmol g^–1^ h^–1^)	general remarks	ref
porphyrin-Ti-MOF	Pt NPs (3 wt %)	photocatalyst (10 mg), aqueous solution (270 mL; 10 mmol ascorbic acid as sacrificial agent), irradiation source (300 W Xe lamp, λ > 420 nm)	8.52	AQY ∼ 0.26% at 380–420 nm	(397)
Cu^2+^-porphyrin MOF	Pt single atom (12 wt %)	photocatalyst (5 mg), aqueous solution (0.1 M ascorbic acid as sacrificial agent), irradiation source (300 W Xe lamp, λ > 420 nm),5 °C	11	good reusability and stability after 4 consecutive cycles with a total reaction time of 20 h	(398)
Pd-PCN-222(Hf)(porphyrin-Pd/Hf)	Pt NPs (0.92 wt %)	photocatalyst (5 mg), solution (10 mL CH_3_CN, 0.25 mL H_2_O and 2.5 mL TEOA), irradiation source (300 W Xe lamp, λ > 420 nm)	23	good reusability and stability after 3 consecutive cycles with a total reaction time of 9 h	(281)

### 6.5 Other MOFs as Photocatalysts for H_2_ Generation

Besides the above commented well-established MOF structures, there is a series^399^ of studies using other MOFs as heterogeneous photocatalysts for H_2_ generation. Some of them are compiled in Table 7. These reports clearly show the continuous growth of this area by utilizing the synthetic flexibility and unique properties offered by MOFs to design efficient photocatalysts. At this point, it is interesting to comment that even though rigid aromatic polycarboxylates are the most common ligands for those MOFs employed so far as photocatalysts, the field does not necessarily need to be constrained to these ligands. The use of other types of ligands such as hydroxycarboxylate^400^ or hydroxamate^401^ and even bonding atoms different from oxygen, such as nitrogen and phosphorus, having interesting light absorption properties can expand significantly the field, moving away from conventional aromatic polycarboxylates.

**Table 7:** Photocatalytic H_2_ Generation under Visible Light Irradiation Using Other MOFs as Photocatalysts

photocatalyst	cocatalyst	reaction conditions	H_2_ production	general remarks	ref
Ti-MOF having TiO chains and 4,4′,4′′,4′′′-(pyrene-1,3,6,8-tetrayl) tetrabenzoic acid	Pt NPs (3.38 wt %)	photocatalyst (40 mg), solution (16.38 mL CH_3_CN, 0.35 mL H_2_O), TEA (3.27 mL) as sacrificial reagent, irradiation source (300 W Xe lamp, λ > 380 nm)	∼1.5 mmol g^–1^ h^–1^	AQE at 440 nm of 0.43%. stable catalyst and H_2_ production in four consecutive uses with a total time of 16 h	(402)

Zn_2_(BODIPY)(BPDC)_2_]·H_2_O(CCNU-1)	Pt NPs (0.13 wt %)	photocatalyst (50 mg), aqueous solution (50 mL; 0.1 M ascorbic acid as sacrificial agent, irradiation source (300 W Xe lamp, λ > 380 nm)	4.68 mmol	AQY 9.06% at 420 nm. The catalyst is stable upon reuse with slight decrease of activity after 3 cycles with a total time of 9 h	(403)
MOF-253-Pt	Pt (0.53 mM)	photocatalyst in CH_3_CN/H_2_O (1:1), 15% TEOA (v/v), pH 8.5, 100 mL solution, 300 W Xe lamp with a 420 nm cutoff filter	3 μmol (30 h)	highest quantum efficiency of 1.63% was observed at 440 nm	(278)

ZnCdS-ZIF-67	Zn_0.5_Cd_0.5_S (200 mg)	photocatalyst (10 mg), 30 mL of 10 vol% lactic acid (sacrificial agent), 5 W LED lamp	23264 μmol g^–1^ h^–1^	AQE was 6.95% at 420 nm. catalyst used three cycles with no decay while some loss is observed in the 4th cycle	(404)
					
Au_25_@ZIF-8@TiO_2_-ReP	Au and ReP	photocatalyst (5 mg), 1 mL TEOA as electron donor, 10 μmol [Ru(bpy)_3_]Cl_2_·6H_2_O as photosensitizer, solvent (5 mL, CH_3_CN:H_2_O; 4:1), irradiated with 300 W Xe lamp (420 nm cutoff filter) with the light intensity of 100 mW·cm^–2^	14.9 μmol (2.2 mmol h^–1^g^–1^)	catalyst was used three times with no decay	(405)

eosin Y-Ni@MOF-5	Ni NPs	Ni@MOF-5 (50 mg), 70 mg eosin Y in 100 mL TEOA-H_2_O (10%, v/v, pH 11), irradiation with 300 W xenon lamp at 420 nm cutoff filter	30 mmol g^–1^(Ni) h^–1^	AQE of 16.7% at 430 nm. catalyst was stable four cycles	(287)

MIL-167(Ti)		photocatalyst (30 mg), CH_3_CN (23.5 mL), TEA (4.7 mL), H_2_O (0.5 mL), 40 °C, UV light	257 μmol g^–1^ h^–1^	reaction rate is 2.6 higher for MIL-167 compared to MIL-125-NH_2_	(400)
MUV-11(Ti)	Pt(IV) (1 wt %)	photocatalyst (1 mg/mL), H_2_O:MeOH solution (4:1 v/v%), solar simulator (253 W), 5 h, room temperature	1.7 μmol·g^–1^	mechanism based on ligand-to-metal charge transfer generates active Ti^3+^ species	(401)

## 7 Photocatalytic OER under Visible Light Irradiation

OWS comprises the concerted occurrence of HER and OER. While photocatalytic HER in the presence of sacrificial electron donors has been widely studied, the thermodynamically and kinetically more demanding OER has been considerably less researched. Even though holes in the HOCO of several robust MOFs should have sufficient oxidation potential to promote OER (see Figure 8), experimental evidence of photocatalytic OER is still very limited.

Photocatalytic water oxidation to O_2_ is a challenging and demanding reaction of high interest. not only in OWS but also for other solar-driven production of sustainable fuels, particularly photocatalytic CO_2_ reduction and N_2_ fixation.^26,406^ The number of reports describing the photocatalytic OER under visible light irradiation promoted by MOFs is still very scarce, and clearly the process requires much more research (Table 8). In a series of studies, OER under visible irradiation in the presence of MOFs has been possible due to the use of homogeneous photosensitizer such as Ru(bpy)_3_^2+^. In other cases, the Ru complex as visible light antenna has been attached to the MOF structure, a fact that can favor the photocatalytic charge separation as well as the stability of the Ru complex, catalyst recovery, and reuse.

**Table 8:** Photocatalytic O_2_ Generation Using MOF Materials under Visible Light Irradiation

photocatalyst	cocatalyst	reaction conditions	production	general remarks	ref
MIL-101(Cr)	Co_3_O_4_(3.9 wt %)	photocatalyst (12.5 mg), photosensitizer ([Ru(bpy)_3_]Cl_2_, 0.05 mmol), aqueous solution (50 mL; 10 mM sodium borate), sacrificial agent (0.375 mmol of Na_2_S_2_O_8_), pH 9, irradiation source (300 W lamp λ > 420 nm),70 min	∼14 mmol g^–1^	9-fold higher activity than unsupported Co_3_O_4_	(327)
MIL-101(Fe)-NH_2_		photocatalyst (1 mg), photosensitizer ([Ru(bpy)_3_]^2+^, 0.02 mM), sacrificial agent (Na_2_S_2_O_8_, 0.08 mM), sodium borate buffer (10 mL, initial pH 10), irradiation source (λ ≥ 420 nm, 52.8 mW cm^–2^), 10 min	36.5 mmol g^–1^	photocatalytic activity decreases more than 60% after the third use after 30 min	(408)
ZIF-67@Co-MOF-74		photocatalyst (1 mg), borate buffer solution (80 mM, initial pH 9, 10 mL), photosensitizer ([Ru(bpy)_3_]^2+^, 1 mM), sacrificial agent (Na_2_S_2_O_8_, 80 mM), irradiation source (Xe lamp 300 W, λ > 420 nm),36 min	122 mmol g^–1^	AQY 11.3%, good stability during 5 consecutive cycles with total time of 200 min	(409)
[Ru(tpy)(dcbpy)(OH_2_)]^2+^ doped UiO-67^a^		photocatalyst (15 mg), aqueous solution (HNO_3_, pH 0.5, 5 mL) + cerium ammonium nitrate solution (0.15 mmol), 1 h	42 μmol g^–1^	1.2 wt % initial Ru content leaches after use	(410)

Co_4_@MIL-100 (Fe)		photocatalyst (12 mg), sodium borate buffer (10 mM), pH 8.0, Na_2_S_2_O_8_ (0.250 mmol), [Ru(bpy)_3_]Cl_2_ (0.05 mmol), 70 min	7.5 mmol g^–1^	reused with no loss of activity	(411)
Co_4_: Co_4_(PW_9_O_34_)_2_(H_2_O)_2_]^10–^					

Bi-MOF		photocatalyst (30 mg), water (30 mL), sacrificial agent (AgNO_3_, 30 mg), 5 °C, 300 W Xe arc lamp	25.1 mL g^–1^ h^–1^		(412)
[Bi(BTC)(DMF)]·DMF(CH_3_OH)_2_		photocatalyst (50 mg) suspended in 50 mL aqueous solution with 100 mg AgNO_3_ as the electron acceptor agent, UV/vis light irradiation.	17.6 mL g^–1^ h^–1^	catalyst was not water stable after photocatalysis	(413)
Bi-mna^b^		photocatalyst (50 mg), water (50 mL), sacrificial agent (AgNO_3_, 100 mg), 5 °C, irradiation source (Xe lamp with λ > 440 nm),5 h	20 mL g^–1^		(414)
CoPi-MIL-53(Al)	CoPi	photocatalyst (20 mg), 20 mL water, 20 mg AgNO_3_, Xe lamp (300 W)	∼130 mL g^–1^ h^–1^	activity decreased after 7 h (14000 μL)	(415)
Cd-TBAPy^c^	CoPi (0.4 wt %)	catalyst (50 mg), aqueous solution (100 mL, 1 mM AgNO_3_), irradiation source (300 W xenon lamp, λ ≥ 420 nm)	1.63 mmol g^–1^ h^–1^	AQY 5.6% at 420 nm	(407)
Hg-based MOF ([Hg(Bpbp)(SCN)_2_]n (CQNU-1^d^		catalyst (25 mg), CH_3_CN (45 mL), H_2_O (1 mL), sacrificial agent (AgNO_3_, 1 mL), irradiation source (300 Xe lamp and AM1.5G filter), 3 h	136 μmol g^–1^ h^–1^	catalyst stable for 3 consecutive cycles with total time of 9 h	(416)
UiO-66-NH_2_ MoS_2_		photocatalyst (20 mg), H_2_O (20 mL), visible light irradiation (300 W Xe arc lamp and cut off filter >420 nm), AgNO_3_ (0.05 M), 50 min	∼12.5 mmol g^–1^	the photoactivity maintains during four consecutive cycles	(417)

a tpy, 2,2′:6′,2′′-terpyridine; dcbpy, 5,5′-dicarboxy-2,2′-bipyridine.

b mna, 2-mercaptonicotinic acid.

c TBAPy, 1,3,6,8-tetrakis(p-benzoic acid)pyrene.

d Bpbp = 4,4′-bis(4-pyridyl)biphenyl).

In a couple of studies, Cd- and Hg-based MOFs showed photoactivity for the independent generation of O_2_ or H_2_ in the presence of the corresponding sacrificial agents. The Cd-based MOF constituted by 1,3,6,8-tetrakis(*p*-benzoic acid)pyrene as organic ligand exhibited good photocatalytic activity for the O_2_ evolution reaction.^407^ Regardless, the good visible light absorption capacity and photoactivity for the OER further efforts should be made for the development of analogous systems based on environmentally tolerable transition metals. Bi MOFs have shown photocatalytic OER activity, although the stability of these MOFs has not been convincingly supported. In electrocatalysis, it is known that Bi- as well as Cu-MOFs undergo deep structural reconstruction, yielding the real active electrocatalyst. Similarly, in photocatalysis, it could be that the activity attributed to the Bi-MOF is due to Bi_2_O_3_ and other MOF-derived composites. Considering that more efforts should be given to study of OER by MOFs, and the fact that several Bi-MOFs have been proposed as adequate materials for OER, the issue of stability is not minor and certainly requires much deeper study than that devoted up to now. In this regard, MIL-53(Al) structure having Co phosphate as cocatalyst appears to be the most efficient OER photocatalyst so far with a O_2_ productivity of 2600 μL h^–1^ that is among the highest value reported so far.

Table 8 summarizes other reports on the use of MOFs as visible light photocatalysts for OER in the presence of appropriate sacrificial electron acceptors.

It would be important to perform a systematic evaluation of the structurally most robust MOFs as photocatalysts for OER that is currently missing, except a report on the photocatalytic activity of UiO-66-NH_2_ incorporating MoS_2_ NPs. Parameters like influence of ligand substituents and multimetallic MOFs, particle size, facet exposure, density of structural defects, influence of cocatalysts, and type of sacrificial electron acceptor should be consistently screened for MOFs such as UiO-66, MIL-125(Ti), and MIL-100, among many others.

## 8 Photocatalytic OWS to H_2_ and O_2_

Independent photocatalytic HER and OER in the presence of sacrificial agents are steps toward the photocatalytic OWS. The reports on the use of MOFs as photocatalysts for OWS under UV–vis, visible, or artificial and natural sunlight irradiation are more recent.^237,418^ The reader is also directed to some recent related reviews on OWS.^119,237,359,392,419^ Under these conditions, only pure water is used, and HER and OER are occurring concertedly. Considering the vast H_2_ amounts that would be needed and its cost, any commercial use of photocatalytic OWS should avoid the use of purposely added sacrificial agents and should generate H_2_ from fresh or even raw waters, including also salted waters. Table 9 lists the series of MOFs that have employed for this purpose. As previously commented for the photocatalytic H_2_ evolution reaction using sacrificial agents, UiO-66(Zr) and MIL-125(Ti) are the most widely employed MOFs for the photocatalytic OWS, although it appears that availability rather than design or suitability is the main reason for this preference.

**Table 9:** List of MOFs that Have Been Employed as Photocatalysts for OWS

photocatalyst	cocatalyst	reaction conditions	production	general remarks	ref
UiO-66(Zr/Ce/Ti)		photocatalyst (20 mg), H_2_O (20 mL), visible light irradiation (λ > 450 nm Hg–Xe lamp 150 W)	H_2_ (∼210 μmol g^–1^) and O2 (70 μmol g^–1^ h^–1^)	photocatalyst maintained its catalytic activity and crystallinity after reuse	(265)
UiO-66(Zr)-NH_2_	Pt and MnOx	photocatalyst (10 mg), H_2_O (100 mL), 5 °C, irradiation source (Xe lamp λ > 400 nm)	H_2_ (19.6 μmol g^–1^ h^–1^) and O_2_ (10.1 μmol g^–1^ h^–1^)	HO^•^ radicals detected during the photocatalytic reaction	(420)
MIL-125(Ti)	CoPi and Pt NPs	photocatalyst (30 mg), H_2_O (30 mL), Xe lamp (300 W)	H_2_ (42.33 μL h^–1^) and O_2_ (21.33 12 L h^–1^)	cocatalysts reduce overpotentials of half-reactions and improve the photoinduced charge separation	(421)
MIL-125(Ti)-NH_2_	Pt and RuO_*x*_ NPs	photocatalyst (20 mg/20 mL H_2_O), natural sunlight irradiation (100 mW × cm^–2^), ambient temperature 30 °C, 10 h	H_2_ (27 μmol g^–1^) and O_2_ (14 μmol g^–1^)	photocatalyst (20 mg/20 mL H_2_O), natural sunlight irradiation (100 mW × cm^–2^), ambient temperature 30 °C, 10 h	(422)
MIL-125(Ti)-NH_2_ plasma treated		photocatalyst (20 mg), H_2_O (20 mL), simulated sunlight irradiation (1 sun), 35 °C, 22 h	H_2_ (∼83 12 mol g^–1^) and O_2_ (29 (19.6 12 mol g^–1^ h^–1^)	catalyst is stable for at least three consecutive cycles	(357)
Al-2-amino BDC	Ni^2+^	photocatalyst (30 mg), H_2_O (30 mL), Xe lamp (300 W), 2.5 h	H_2_(66.7 μmol·g^–1^) and O_2_(33.3 μmol·g^–1^)	Ni^2+^ is essential to promote HER	(51)
2D-Ni-phosphonate MOF		photocatalyst (5 mg), H_2_O (20 mL), simulated sunlight irradiation (150 W Xe, AM 1.5G filter), 22 h	H_2_(46 μmol·g^–1^) and O_2_(18 μmol·g^–1^)	catalyst can be reused three times with slight decrease of activity	(423)
Ti-squarate		photocatalyst (2.5 mg), H_2_O (20 mL), 35 °C, simulated sunlight irradiation (Xe–Hg lamp 150 W through an AM 1.5G filter) 22 h	H_2_(672 μmol·g^–1^) and O_2_(260 μmol·g^–1^)	photocatalyst is stable and reusable for at least 10 days	(424)
liposome-MOF	Pt-porphyrin and Ir-bipyridine	photocatalyst containing [Ru(2,2′-bipyridine)_3_]^2+^-based photosensitizers (10 mL), two redox relays (tetrachlorobenzoquinone/tetrachlorobenzohyd rosemiquinone, and the Fe^3+^/Fe^2+^, H_2_O (20 mL), LED light, irradiation,72 h	H_2_ (836 μmol g^–1^) and O_2_ (∼418 μmol g^–1^)	photocatalyst system operates under a *Z*-scheme mechanism	(425)

**Figure 28:**
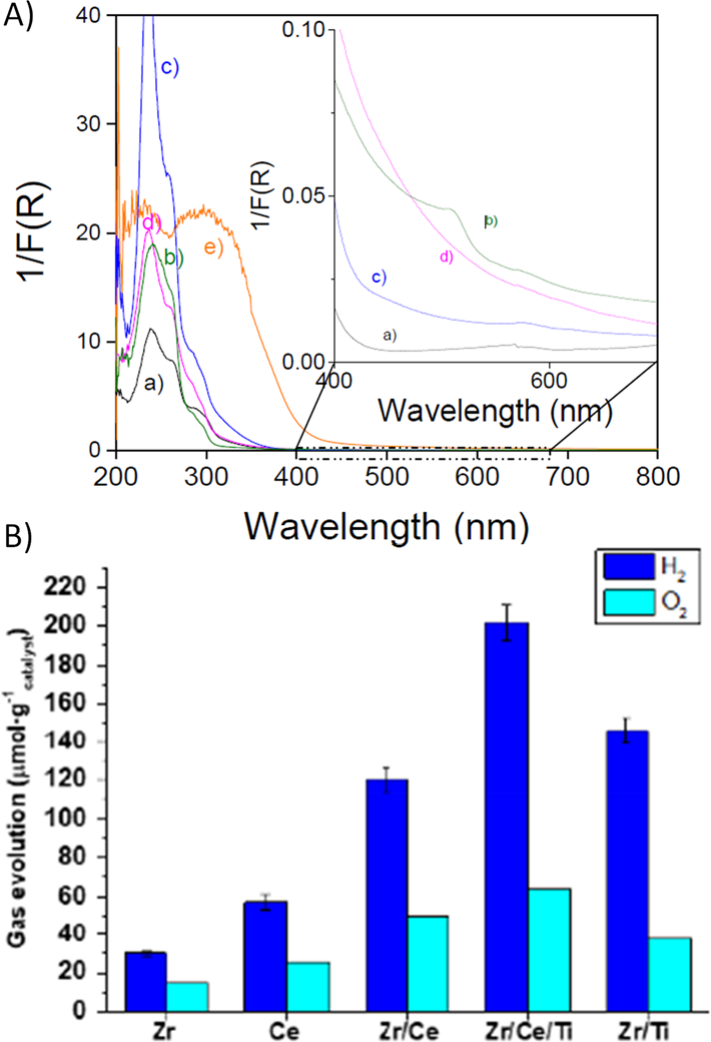
(A) Diffuse reflectance UV–vis spectra of the series of multimetallic UiO-66: (a) UiO-66(Zr), (b) UiO-66 (Zr/Ti), (c) UiO-66(Zr/Ce), (d) UiO-66(Zr/Ce/Ti), and (e) UiO-66(Ce). The inset shows an expansion of the onset of the absorption in the visible region. (B) Photocatalytic gas evolution using different UiO-66 as photocatalysts upon irradiation with visible light (λ > 450 nm). Reaction conditions: light source UV–vis xenon lamp (150 mW cm^–2^) equipped or not with a filter (λ > 450 nm), photocatalyst 20 mg, H_2_O (20 mL), photoreactor volume (51 mL), and reaction temperature (35 °C). Adapted with permission from ref (265). Copyright 2020 Elsevier.

Thus, although UiO-66(Zr) exhibits photocatalytic activity derived from ligand excitation and ligand-to-metal charge-transfer (LMCT) process, its efficiency is far from optimal.^257^ Theoretical studies supported by experimental data have shown a poor overlap and energy alignment between the Zr_6_(OH)_4_O_4_^12+^ nodes and the ligands.^257,258^ This overlap and correct HOCO–LUCO distribution can be improved if other metal ions such as Ti^4+^ or Ce^4+^ is present in the node.^257^ In this context, very recently, our group has reported the preparation of a trimetallic UiO-66(Zr/Ce/Ti) solid and used it as stable and reusable photocatalyst for OWS under visible light irradiation (Figure 28).^265^ For comparison, the photoactivity of a series of mono- and bimetallic UiO-66 (M: Zr, Zr/Ti, Zr/Ce, Zr/Ce/Ti, Ce) analogues was also evaluated. XRD pattern of the UiO-66 series confirms that all of the solids are isostructural to the parent UiO-66(Zr). It was assumed that the bi- or trimetallic MOFs were constituted by metal nodes of Zr^4+^/Ti^4+^ and/or Ce^4+^/Ti^4+^, while the simultaneous presence of Zr^4+^/Ce^4+^ in the same metal node was considered less likely based on XP spectroscopy. The estimated band gap values determined from diffuse reflectance UV–vis spectroscopy for UiO-66(Zr), UiO-66(Zr/Ce), UiO-66(Zr/Ti), UiO-66(Zr/Ce/Ti), and UiO-66(Ce) revealed remarkable differences with values of 3.31, 3.25, 3.10, 3.05, and 2.60 eV, respectively. These measurements confirm the narrower HOCO/LUCO gap for UiO-66(Ce) with respect to conventional UiO-66(Zr) as correctly predicted by prior theoretical calculations proposing UiO-66(Ce) as an ideal visible-light photocatalyst.^257^ The UiO-66(Zr/Ce) having independent monometallic Zr^4+^ and Ce^3+^/Ce^4+^ nodes according to XPS measurements showed a narrower band gap than UiO-66(Zr). Interestingly, the presence of Ti^4+^ ions within the Zr^4+^ and/or Ce^3+^/Ce^4+^ metal nodes resulted in a significant decrease of the band gap with respect to the conventional UiO-66(Zr) accompanied by a red-shift absorption maximum with an absorption onset in the visible region up to about 600 nm.

In the series of multimetallic UiO-66, the UiO-66(Zr/Ce/Ti) sample exhibited enhanced photoactivity for OWS under visible light irradiation (λ > 450 nm; 220 μmol g^–1^ after 22 h) compared with mono- or bimetallic analogues (Figure 28).^265^ Analogous experiment under UV–vis light using UiO-66(Zr/Ce/Ti) in the absence and in the presence of methanol as sacrificial electron donor resulted in a H_2_ production of 225 and 390 μmol g^–1^ after 22 h, respectively. This rather moderate increase of H_2_ production in the presence of methanol was interpreted by proposing that the trimetallic nodes should have operative a specific reaction mechanism catalyzing the H_2_O oxidation to O_2_, suggesting some resemblance with the natural photosynthetic center II.

To get more insights on the superior photoactivity of the UiO-66(Zr/Ce/Ti) for OWS, several spectroscopic studies were carried out.^265^ Thus, XPS together with the estimated band gap from diffuse reflectance UV–vis spectroscopy confirmed that the photocatalyst has an appropriate band alignment to promote the two semireactions involved in the OWS. Transient absorption spectroscopy (TAS) studies revealed that the higher efficiency of the UiO-66(Zr/Ce/Ti) appears to be related with its high efficiency of photoinduced charge separation with formation of electrons and holes in the microsecond time scale. Besides, TAS was employed to confirm the quenching of the triplet excited state of terephthalate by the [Zr_6_O_4_(OH)_4_]^12+^ cluster as well as by the presence of Ti^4+^ or Ce^4+^ salts. The occurrence of charge separation on the UiO-66(Zr/Ce/Ti) was visually evidenced by observing that methyl viologen (MV^2+^) or tetramethylphenylenediamine (TMDPA) as probe molecules of electrons and holes, respectively, became colored upon UV–vis irradiation in the presence of the MOF suspension. Furthermore, the characteristic photoluminescence of the UiO-66 is practically quenched in UiO-66(Zr/Ce/Ti) in comparison with monometallic UiO-66(Zr), a fact that was ascribed to the inhibition of charge recombination in UiO-66(Zr/Ce/Ti) that is assumed to be responsible for the emission in UiO-66(Zr). Overall, this work exemplifies the possibility of tuning the electronic properties of MOFs through metal node engineering and the use of the resulting node-modified MOFs as visible responsive materials with enhanced photocatalytic activity for OWS.

**Figure 29:**
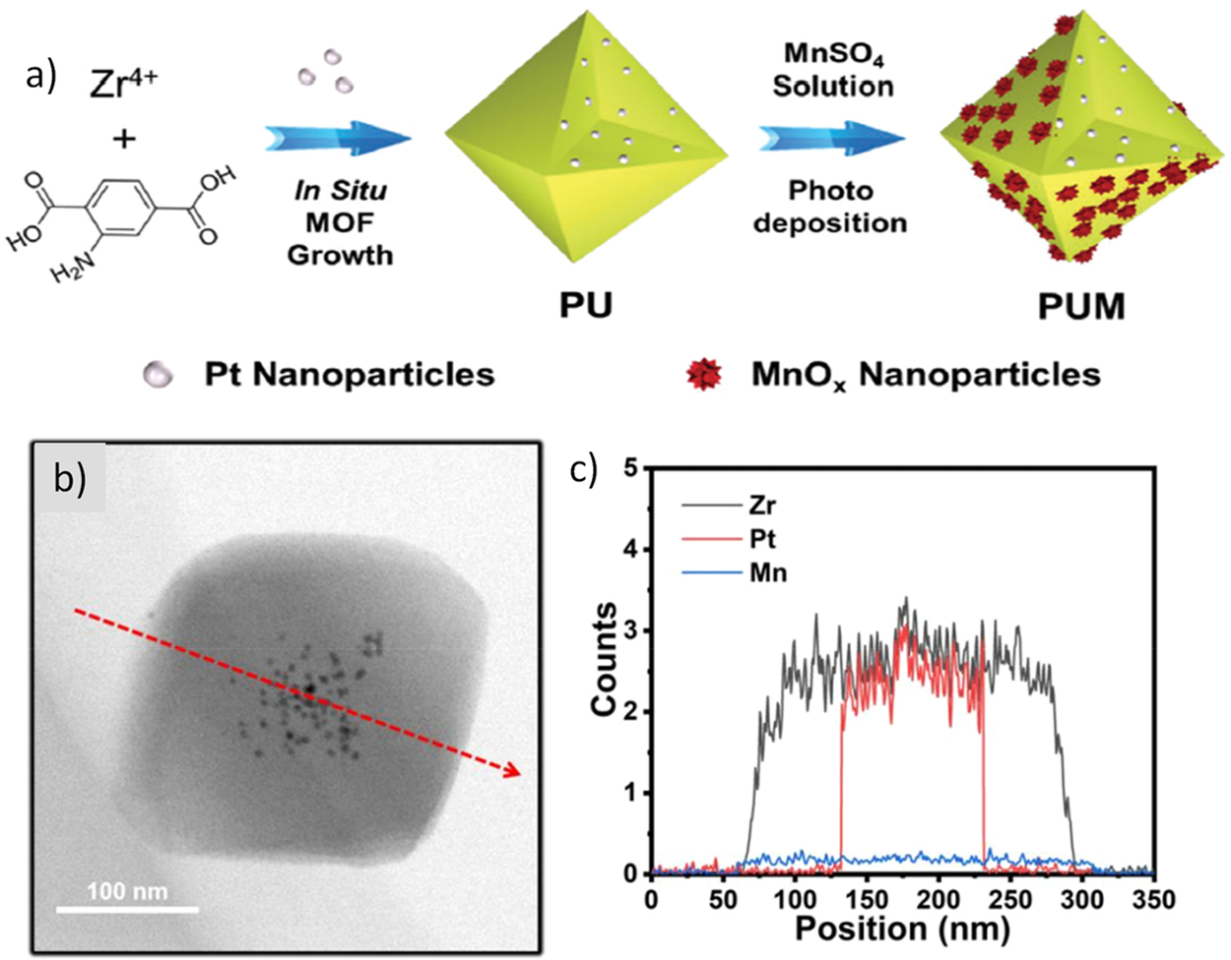
Synthesis of PUM (a). TEM image (b) and EDS elemental mapping (c) across the arrow shown in image (b) of a PUM crystal. P refers to Pt, U refers to UiO-66, and M stands for MnO_*x*_. Reproduced with permission from ref (420). Copyright 2020 Wiley.

Continuing with UiO-66(Zr)-NH_2_ as photocatalyst, the use of spatially separated Pt (3.2 wt %) and MnO_*x*_ (0.1 wt %) NPs as reduction and oxidation cocatalysts, respectively, has resulted in an adequate strategy to enhance OWS efficiency.^420^Figure 29 shows the preparation of UiO-66 with spatially separated Pt NPs (inside) and MnO_*x*_ (external). Thus, photoinduced charge separation leads to the vectorial flow of electrons toward the inner part of the MOF where Pt NPs are located, while holes migrate in the opposite direction to the outer surface of MOF particle where MnO_*x*_ are present. The improvement of photoinduced charge separation of the photocatalyst was assessed by photocurrent measurements, electrochemical impedance measurements, and spectroscopic techniques, including steady-state and time-resolved photoluminescence (PL) and ultrafast TAS. The OWS under visible light irradiation results in the formation of the expected quasistoichiometric amounts of H_2_ (19.6 μmol g^–1^ h^–1^) and O_2_ (10.1 μmol g^–1^ h^–1^). Based on ESR measurements, it was proposed that the holes loaded at the MnO_*x*_ NPs oxidize H_2_O to HO^·^ radicals, which should further evolve to O_2_. Regardless of the room for improvement of photocatalytic activity, this study illustrates the potential of cocatalysts spatially separated internally vs externally or even in different facets to increase the efficiency of MOF-based photocatalysts for the solar-driven OWS. It would be of interest to determine if the observed charge carrier flow (electrons to the interior, holes to the external surface) is due to the specific location of the cocatalysts or due to the structure of the MOF. It would have been convenient in this regard to obtain data from the reverse cocatalyst location (Pt NPs external and MnO_*x*_ internal)

The potential use of the benchmark MIL-125(Ti)-NH_2_ as photocatalyst for the OWS has been reported in recent studies.^357,421,422^ The energy of the HOCO and LUCO electronic states of MIL-125(Ti) meet the thermodynamic requisites for the OWS under UV–vis irradiation (Figure 8), In addition, Ti^4+^ as d^0^ transition metal ion and Ti-O as LMCT state are among the preferred species in photocatalysis. As commented before, MIL-125(Ti) has been employed for the photocatalytic hydrogen evolution in the presence of sacrificial electron donors and UV–vis irradiation. Later, the MIL-125(Ti) solid has been modified with Pt NPs and cobalt phosphate (CoPi) as cocatalysts for the concerted H_2_ and O_2_ evolution from water. Characterization data indicate that the onset absorption of the pristine MIL-125(Ti) (λ = 351 nm) is red-shifted up to about 450 nm in the presence of Pt NPs, a fact that has been associated with a surface plasmonic effect of the metal NPs. This effect is certainly surprising, considering that Pt NPs do not generally exhibit plasmon band in the visible region. The linear sweep voltammograms of the MIL-125(Ti) solid with and without cocatalysts showed that the presence of Pt or CoPi results in a diminution of the overpotentials required for H_2_ and O_2_ evolution, respectively. Similarly, the Nyquist plot of the photocatalysts reveals that the presence of cocatalysts decreases the charge-transfer resistance with respect to pristine MIL-125(Ti). In addition, photoluminescence spectroscopy of the MIL-125(Ti) materials indicates that cocatalysts quench the emission intensity compared to the possible MIL-125(Ti). The less intense emission can be attributed to the more efficient charge separation and lesser charge recombination in the presence of cocatalysts. In summary, this work provides solid evidence supporting that the presence of cocatalysts frequently employed for H_2_ and O_2_ evolution decreases HER and OER overpotentials and favors the photoinduced charge separation, rendering an active photocatalyst for OWS under UV–vis light irradiation. It should be noted, however, that a detailed study of the OER process and its optimization is still missing.

A step forward on the use of MIL-125(Ti) photocatalysts for the solar-driven OWS has been reported by using amino functionalized MIL-125(Ti) containing Pt, CoO_*x*_, or RuO_*x*_ NPs as cocatalysts.^422^ The introduction of the −NH_2_ group on the terephthalate ligand extends light absorption of the material to the visible region. Pristine MIL-125(Ti)-NH_2_ as photocatalyst affords H_2_ and O_2_ productions of about 49 and 23 μmol/g_photocatalyst^–1^, respectively, at 35 °C and under UV–vis irradiation for 22 h. Among the different cocatalysts that were evaluated, the simultaneous loading of Pt and RuO_*x*_ NPs within the MIL-125(Ti)-NH_2_ cavities renders the most active material of the series, promoting H_2_ and O_2_ evolution under UV–vis irradiation with values of 218 and 85 μmol/g photocatalyst^–1^, respectively, after 22 h. Importantly, the Pt/RuO_*x*_-MIL-125(Ti)-NH_2_ photocatalyst was active for the OWS under natural sunlight irradiation reaching productions for H_2_ and O_2_ of 27 and 14 μmol g^–1^ after 10 h during a spring day in Valencia (Spain).^422^ The use of H_2_^18^O during the photocatalytic experiment confirmed the evolution of ^18^O_2_, analyzed by GC-MS, from water. This was one of the first examples showing that the concept of cocatalysts can also apply to a MOF as a strategy to enhance the OWS to quasistoichiometric H_2_ and O_2_ evolution, being possible to use real sunlight irradiation. The efficiency is, however, rather modest, and further optimization is still needed, perhaps by controlling the location of the cocatalyst and optimization of their loading.

**Figure 30:**
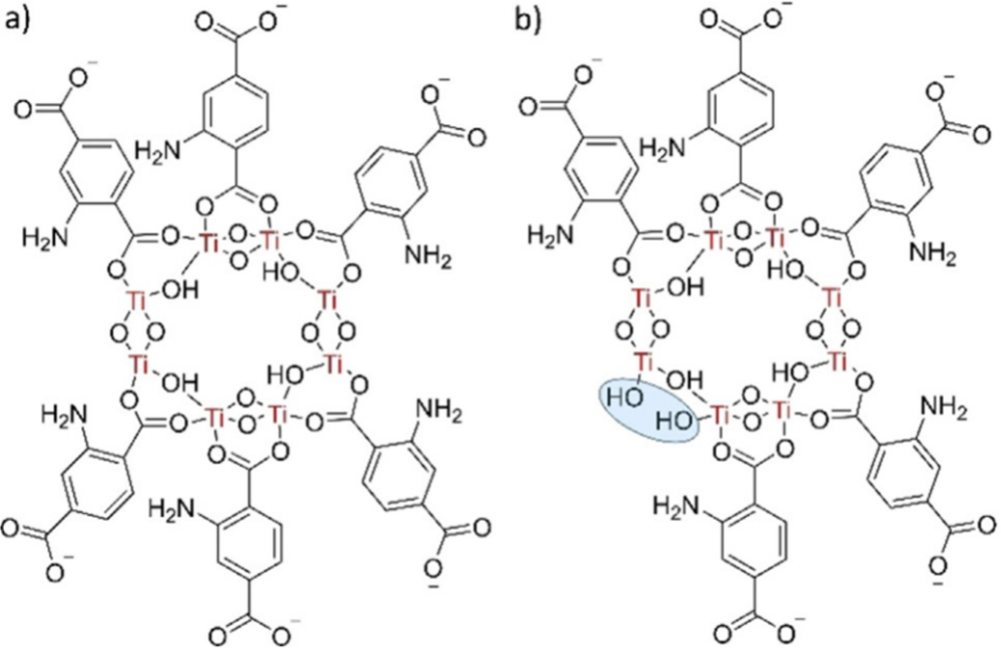
Simplified illustration of ideal (a) and oxygen plasma treated (b) MIL-125(Ti)-NH_2_ materials. Reproduced with permission from ref (357). Copyright 2020 Wiley.

Besides cocatalysts, generation of defects is frequently a valid methodology to boost the photocatalytic activity in metal oxide semiconductors in general, and this strategy has also been applied to MOFs. Very recently, it has been reported that oxygen plasma treatment of MIL-125(Ti)-NH_2_, causing mainly decarboxylation and generating structural defects, results in a 2-fold enhancement of activity respect to the pristine MOF for the OWS under both UV–vis and simulated sunlight irradiation.^357^ In addition plasma-treated MOF was reusable with no decay in photocatalytic activity. Characterization data by XPS, FT-IR spectroscopy, isothermal N_2_ and Ar adsorption and XRD indicate that the oxygen plasma treatment induces partial decarboxylation of the 2-aminoterephthalate ligands in MIL-125(Ti)-NH_2_ with concomitant formation of new Ti–OH bonds, accompanied by some decrease of porosity and particle size while maintaining the crystal structure (Figure 30). Moreover, thermogravimetry and combustion elemental analysis was employed to estimate the experimental formula of pristine and oxygen plasma-treated MIL-125(Ti)-NH_2_ samples that was compared with the ideal formula (Ti_8_O_8_)(OH)_4_(C_6_H_3_C_2_O_4_NH_2_)_6_. More specifically, the 5 and 10 min plasma-treated MIL-125(Ti)-NH_2_ samples showed a decrease in the organic carbon content together with an increase of the titanium percentage. Less intuitive is the observation on an opposite behavior for the MOF sample over treatment for 15 min. This work proposes a simple procedure for the enhancement of MOF activity via defect engineering by plasma or other related defect-generating treatment methodologies.

**Figure 31:**
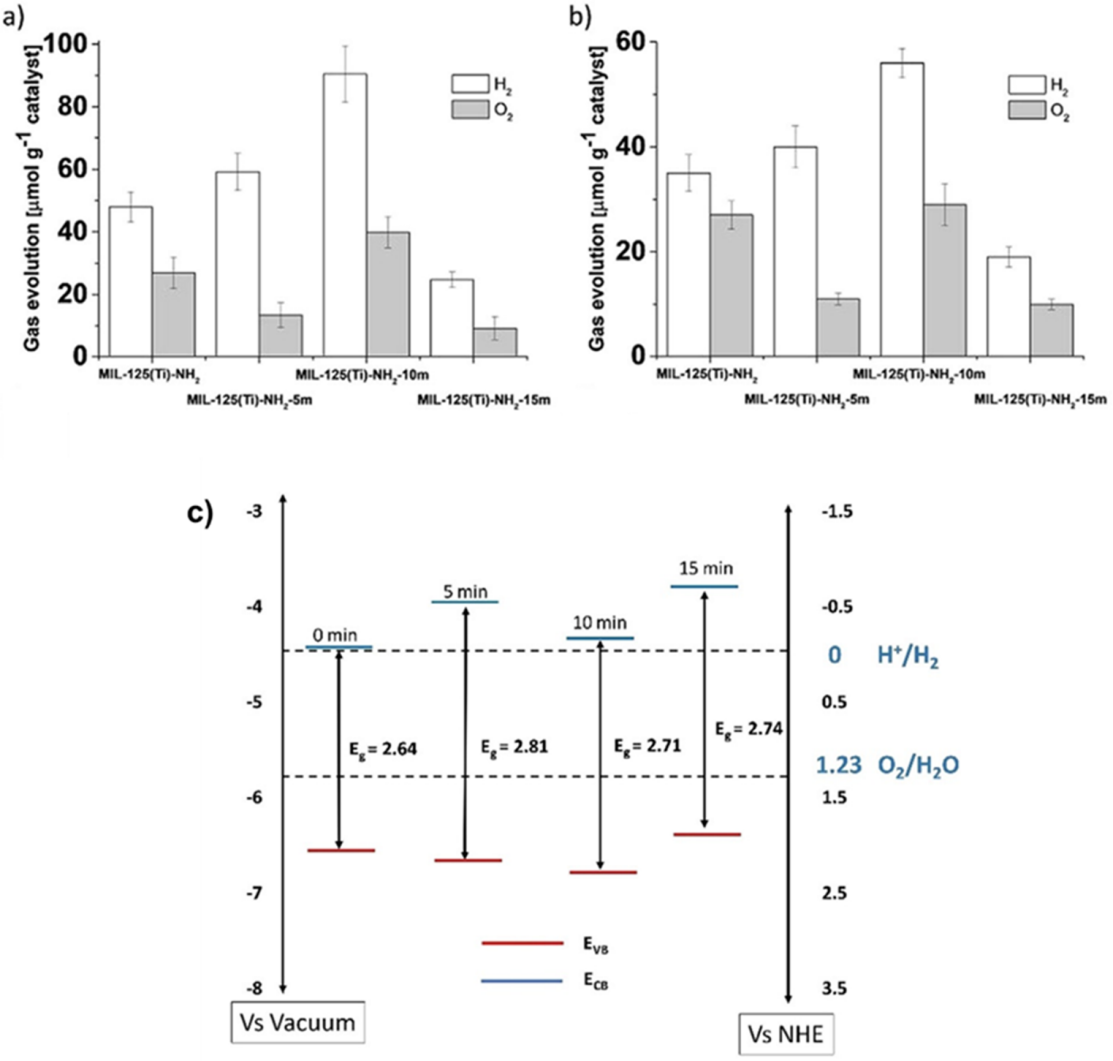
OWS using MIL-125(Ti)-NH_2_ materials as photocatalysts under UV/vis (a), visible (λ > 450) (b), and energy levels of the MIL-125(Ti)-NH_2_ samples without (0 min) or with oxygen plasma treatment (5, 10, and 15 min) under vacuum of NHE as reference (c). Adapted with permission from ref (357). Copyright 2020 Wiley.

In this way, an oxygen-plasma treatment of MIL-125(Ti)-NH_2_ for 10 min was found to result in the optimal photocatalyst for OWS under both UV–vis light and simulated sunlight irradiation (Figure 31).^357^ Diffuse reflectance UV–vis spectroscopy and valence band potential estimated by XPS showed that the most active sample exhibits slightly higher band gap than pristine MIL-125(Ti)-NH_2_ with somewhat more positive and negative HOCO and LUCO energy values that should favor from the thermodynamic point of view the water oxidation and reduction, respectively. Comparison with the performance of the other oxygen plasma treated MIL-125(Ti)-NH_2_ samples for less or longer times seems to point to the importance of a more positive HOCO energy level in MIL-125(Ti)-NH_2_ such as in the sample treated for the optimal time to favor the thermodynamically more demanding water oxidation half-reaction. The use of isotopically labeled H_2_^18^O confirmed that the generated oxygen evolves from water, if ^18^O/^16^O isotopic exchange does not occur in the time frame of photocatalytic experiment. Moreover, the smaller average particle size of oxygen plasma-treated MIL-125(Ti)-NH_2_ sample with respect to the pristine MOF was claimed also to be beneficial for the photocatalytic process. Interestingly, the MIL-125(Ti)-NH_2_ 10 min sample was also the most active for the photocatalytic hydrogen generation from water in the presence of methanol as sacrificial electron donor agent. This enhancement was attributed again to the better alignment of HOCO position for water reduction. Photocurrent measurements showed that the optimized oxygen plasma-treated MIL-125(Ti)-NH_2_ sample exhibits a lower 0.1 V onset potential compared to the pristine MIL-125(Ti)-NH_2_ sample, together with a higher photocurrent of the former when methanol is present during the photocurrent measurements.

**Figure 32:**
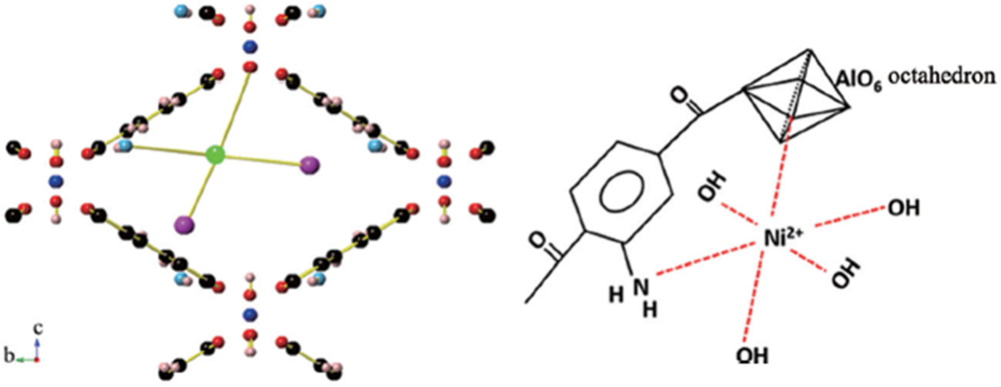
Proposed Ni coordination in MIL-53(Al)-NH_2_ MOF Ni(II) complex was found to be necessary to promote HER: blue, Al; green, Ni; red, O; light-blue, N, black, C; purple, OH. Reproduced with permission from ref (51). Copyright 2017 Wiley.

Molecular metal complexes are, besides metal or metal oxide NPs, also well-known cocatalysts.^141,142,145,271,275,426,427^ In one study, Ni^2+^ ions coordinated to the NH_2_ group of the 2-aminoterephthalate organic ligand of the MIL-53(Al)-NH_2_ solid resulted in an efficient photocatalyst for the OWS.^51^ Characterization by XANES, EXAFS, and FT-IR spectroscopies confirmed the presence of octahedrally coordinated Ni^2+^ to one nitrogen of the 2-aminoterephthalate, one oxygen of the AlO_6_, and four HO groups (Figure 32). As previously commented, MIL-53(Al)-NH_2_ was known to be active for H_2_O oxidation to O_2_. The use of Ni(II)-MIL-53(Al)-NH_2_ as photocatalyst in the presence of Ag^+^ as sacrificial electron acceptor resulted in a solid more active (155 μmol h^–1^) than pristine MIL-53(Al)-NH_2_ (16.5 μmol h^–1^) for the O_2_ evolution reaction. Upon addition of Ni^2+^ ions coordinated to NH_2_, the resulting Ni(II)/MIL-53(Al)-NH_2_ was employed for OWS. Control experiments revealed that the Ni(II)-MIL-53(Al)-NH_2_ is able to generate H_2_ (36 μmol h^–1^) in the presence of methanol as sacrificial electron donor, while the MIL-53(Al)-NH_2_ was inactive for the same purpose. The experimental evidence suggests that role of Ni(II) is to act as cocatalysts to facilitate the H_2_ evolution. The Ni(II)-MIL-53(Al)-NH_2_ can be used as photocatalysts for the stoichiometric 2:1 production of H_2_ and O_2_ under UV–vis irradiation.

Electrochemical measurements reveal that the presence of Ni^2+^ coordinated to MIL-53(Al)-NH_2_ increases the photocurrent under UV–vis irradiation with respect to the pristine MOF, indicating a more efficient charge separation.^51^ Besides, it was shown that irradiation of Ni(II)-MIL-53(Al)-NH_2_ with UV–vis results in the conversion of Ni^2+^ to Ni^+^, a fact further confirmed by the blue color observed during the reaction and ESR measurements. Thus, it was proposed that Ni^+^ center is the responsible of the observed HER. The presence of Ni^2+^ in the MIL-53(Al)-NH_2_ also causes a photoluminescence intensity diminution respect to the parent MIL-53(Al)-NH_2_ solid, attributed to the more efficient electron–hole pair separation when Ni^2+^ are present in the solid. Besides photocurrent measurements, electrochemical spectroscopy impedance also confirms that the presence of Ni^2+^ in the MIL-53(Al)-NH_2_ favors the photoinduced generation of electrons and holes. Overall, this study has shown that besides metal NPs and considering that 2-aminoterephthalate is a ligand present in many different MOFs, metal ions coordinated to amino groups of the linker may act as cocatalyst in the OWS under UV–vis irradiation.

**Figure 33:**
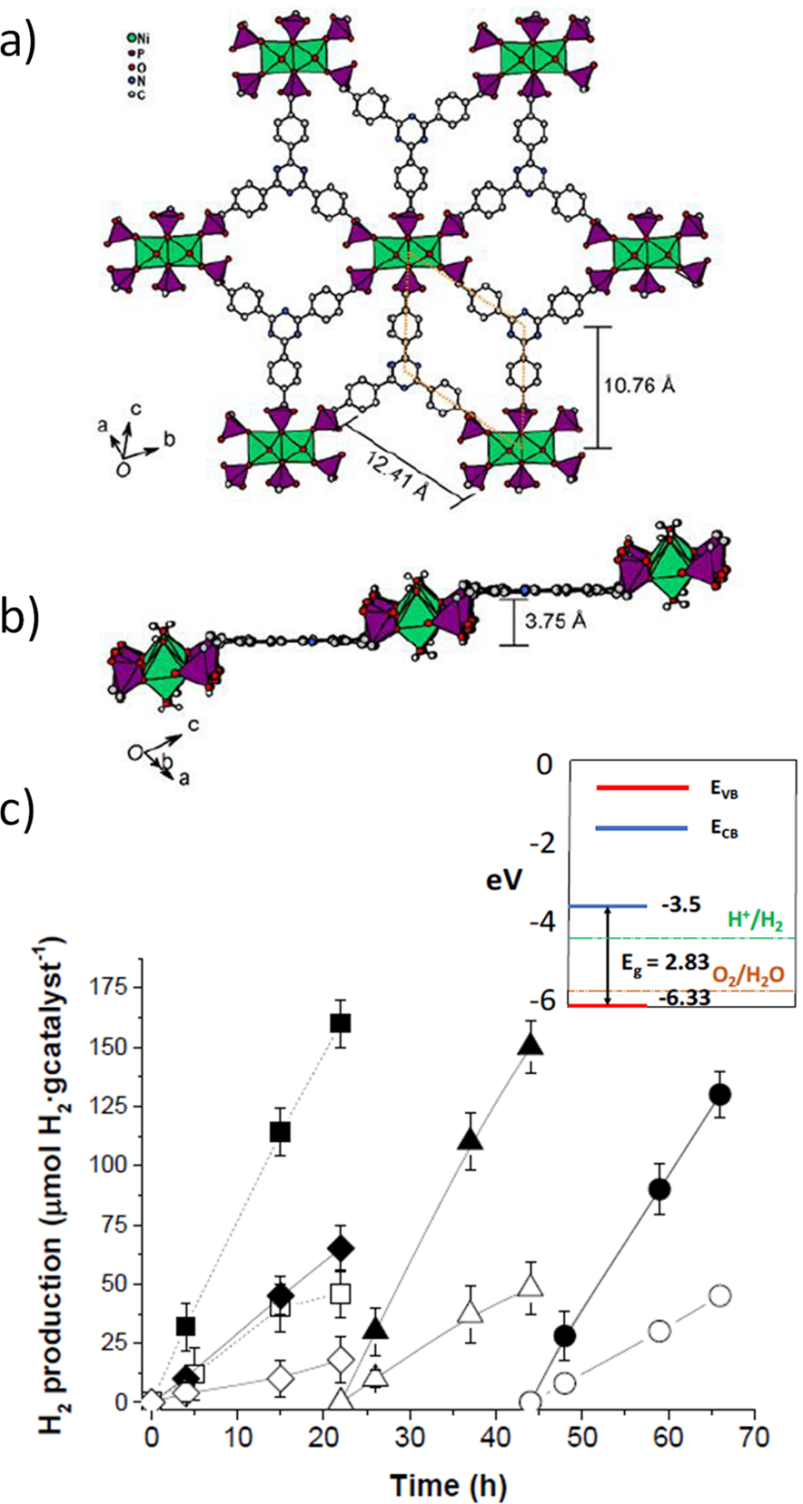
Frontal view (a) and cross sectional (b) views of the metal organic layer of 2D-Ni-phosphonate MOF. Ni, P, C, N, O, and H are represented in green, purple, gray, blue, red, and white, respectively. (c) H_2_ evolution photocatalytic data upon irradiation. The inset shows the band potential of the 2D Ni-phosphonate MOF.^423^ Reproduced with permission from ref (423). Copyright 2020 Springer Nature.

Most of the MOFs used as photocatalysts for the OWS are carboxylate MOFs. A rare exception are those MOFs in which the groups coordinating to the metal nodes are phosphonates.^428^ Metal–phosphonate coordination bonds are generally stronger than metal–carboxylate bonds and, therefore, phosphonate MOFs may enjoy in principle higher stability.^428,429^ In this context, a microporous 2D Ni(II)-phosphonate MOF has been reported as a photocatalyst for OWS.^423^ The 2D crystalline phosphonate-MOF is constituted by the redox active Ni^2+^ sites present in the bioctahedral bimetallic nickel [Ni_2_O_6_(H_2_O)_4_] nodes with exchangeable coordination positions and the tritopic phosphonate ligand 4,6-tris[4-(phosphonomethyl)phenyl]-1,3,5-triazine (Figure 33). Worth noting is that the morphology of the phosphonate MOF particles is like platelets of a few nm thickness. defining a 2D MOF. Diffuse reflectance UV–vis shows that the phosphonate MOF absorbs in the visible region with an estimated band gap of 2.83 eV. The band gap together with valence band energy determined by XPS confirms an appropriate band alignment for both H_2_ and O_2_ evolution (Figure 33). Photocurrent measurements upon positive polarization of an electrode having an active electrode showed that upon illumination using a Xe lamp of the Ni-phosphonate MOF exhibits a photocurrent in the absence as well as in the presence of methanol as electron donor. More interestingly, the Ni-phosphonate MOF exhibits a H_2_ production under both UV–vis or simulated solar light irradiation in the absence of sacrificial electron donors of 160 and 46 μmol g^–1^ after 22 h, respectively (Figure 33). Thus, the photocatalytic activity of the 2D Ni-phosphonate MOF compares favorably with the use of Pt and RuO_*x*_ NPs supported on MIL-125(Ti)-NH_2_ solid.^422^ The presence of methanol as sacrificial electron donor in the photocatalytic system upon UV–vis or simulated sunlight irradiation affords 2200 and 1700 μmol g^–1^, respectively, of H_2_ at 20 °C for 22 h. This high increase of H_2_ production in the presence of methanol indirectly indicates that the water oxidation to O_2_ is the limiting step during OWS. The estimated STH efficiencies for HER in the presence or absence of methanol under UV–vis irradiation were still very low, with the values of 0.001 and 0.0001%, respectively. This means that probably in the other MOFs, the unexpected STH efficiency values are also much too low. It should be mentioned that a clear way to increase this efficiency should be the incorporation of cocatalysts for H_2_ and O_2_ evolution that should also enhance the photoinduced charge separation. Overall, this study opens new avenues for the development of new cost-effective and visible light responsive MOFs based on unconventional phosphonate ligands for the solar-driven OWS.

**Figure 34:**
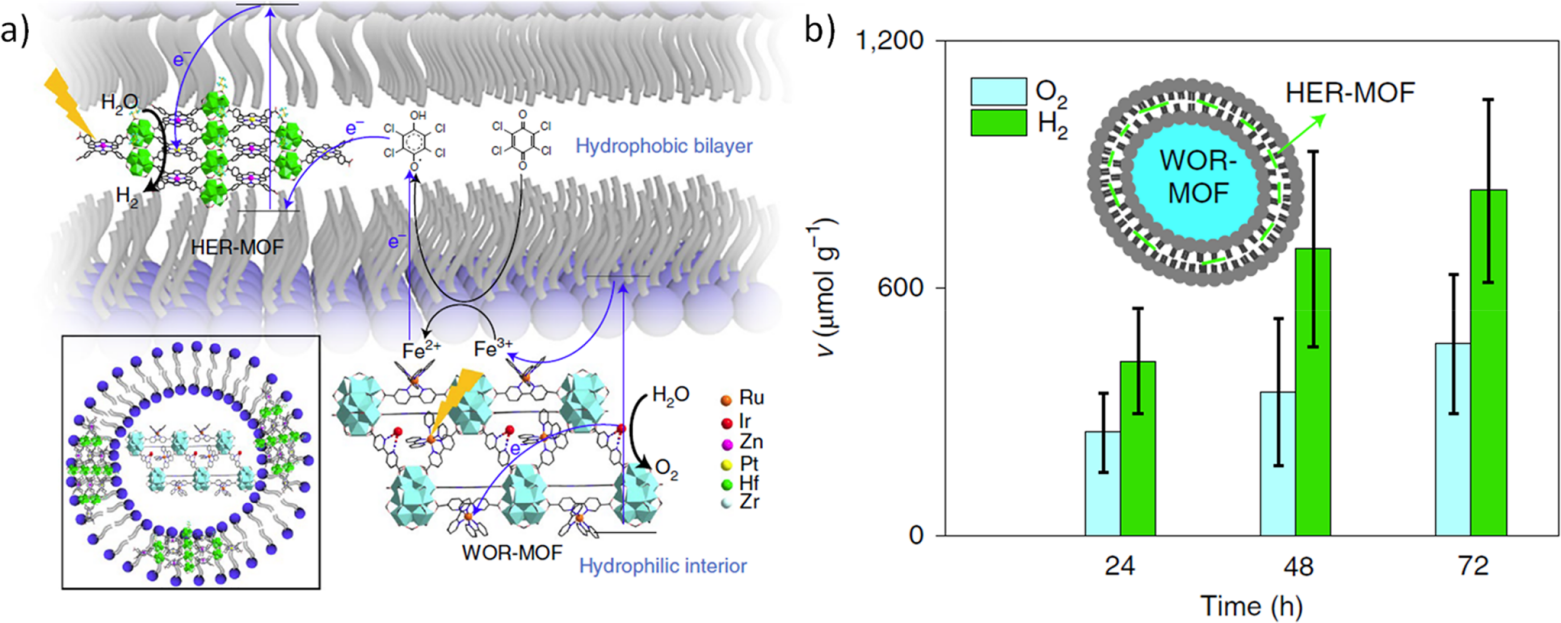
(a) Structure of the liposome-MOF (LP-MOF) arrangement for photocatalytic OWS and the “*Z*-scheme” electron-transfer chain proposed for the LP-MOF system. The bilayer in the illustration represents a section of the liposome. The HER-MOF for hydrogen evolution is incorporated between the hydrophobic chains, and the WOR-MOF for water oxidation is in the aqueous phase. Both MOFs are represented by polyhedron–ball–stick models; The redox shuttles TCBQ/TCBQH (tetrachlorobenzoquinone/tetrachlorobenzohydrosemiquinone) and Fe^3+^/Fe^2+^ connect the HER and WOR sides, functioning in the lipid phase and the aqueous phase, respectively. Electron-transfer chains (shown by blue arrows) and photons (represented by yellow lightning shapes) form the “*Z*-scheme”. Inset: schematic representation of the liposome with the two MOFs embedded within the hydrophobic bilayer (HER-MOF) and the hydrophilic interior (WOR-MOF). (b) Photocatalytic OWS by the LP-HER-WOR-MOF under visible-light irradiation (light source: 400 nm LED + 450 nm LED) showing H_2_ and O_2_ produced in a roughly 2:1 ratio.^425^ Reproduced with permission from ref (425). Copyright 2021 Springer Nature.

An interesting study has reported the possibility to develop a MOF embedded in a liposome to facilitate the photocatalytic OWS under visible light irradiation (Figure 34).^425^ In this well-organized system, the HER and OER processes were separately incorporated in the hydrophobic and hydrophilic part of the MOF, respectively. Specifically, the HER site was built by hydrophobic Hf_6_(μ_3_-O)4(μ_3_-OH)_4_ clusters functionalized with pentafluoropropionate groups and porphyrin [(TCPP)Zn]/[(TCPP)Pt; where TCPP = *meso*-tetra(4-carboxyphenyl)porphyrin] linkers where (TCPP)Pt acts as HER cocatalyst. The OER site is composed by Zr_12_(μ_3_-O)_8_(μ_3_-OH)_8_(μ_2_-OH)_6_ clusters together with [Ru(2,2′-bipyridine)_3_]^2+^-based photosensitizers and Ir-bipyridine catalytic centers. This photocatalytic system can achieve an AQY of 1.5 ± 1% at 436 nm. Unfortunately, reaction times longer than 36 h resulted in photocatalyst degradation, a fact attributed to the possible generation of reactive oxygen species during the reaction.

**Figure 35:**
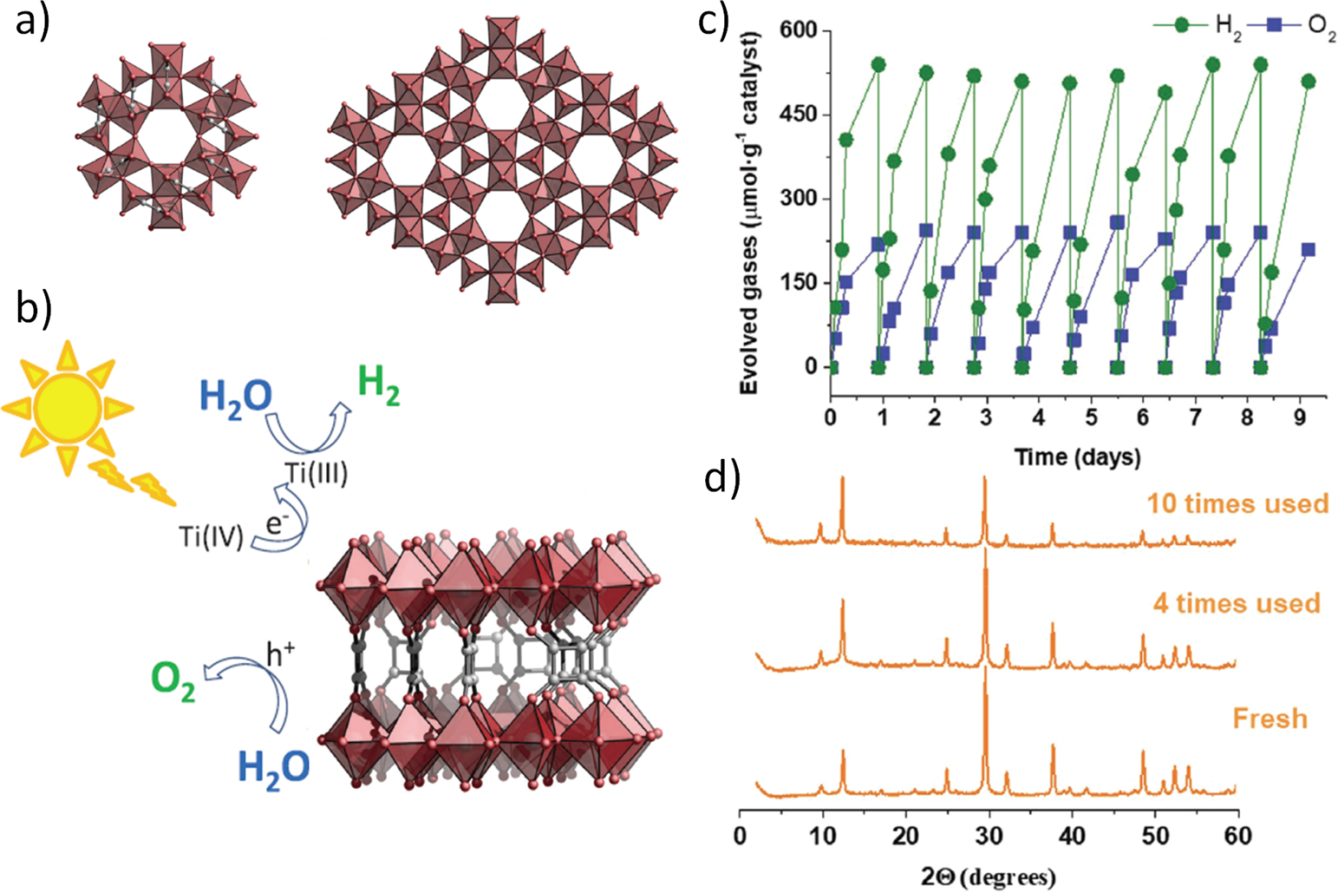
(a) View along [001] of the (a) IEF-11 structure, (b) Ti–O layer SBU, and squarate linkers connecting the different Ti–O layers, (c) Photocatalytic OWS for 10 consecutive cycles using IEF-11 under simulated sunlight irradiation and (d) PXRD patterns of the fresh, four- and ten-times used IEF-11 sample. Reaction conditions: photocatalyst (5 mg), H_2_O (20 mL), simulated sunlight irradiation (Hg–Xe lamp 150 W through an AM 1.5G filter), 35 °C.^424^ Reproduced with permission from ref (424). Copyright 2021 Wiley.

Recently, a novel porous Ti^4+^ squarate MOF (IEF-11) with chemical formula Ti_2_O_3_(C_4_O_4_) (C_4_O_4_ corresponding to squarate ligand) (Figure 35) has shown outstanding photocatalytic activity and stability for the OWS under simulated sunlight irradiation in the absence of any cocatalyst or sacrificial agent.^424^ The IEF-11 solid exhibits 2D layers of interconnected TiO_6_ and TiO_5_ polyhedra hold by perpendicular squarate pillars. The nanosized crystals (85 ± 30 nm) precluded single crystal XRD and the structure of IEF-11 was determined by electron diffraction of the nanocrystals. The excellent catalytic activity attributed to the good band alignment for the OWS that allows IEF-11 to operate under a LMCT mechanism from squarate ligands to the 2D Ti^4+^ layer as revealed by ESR measurements. The activity and stability of IEF-11 solid was remarkable for 10 days in the absence of cocatalyst under simulated sunlight irradiation and ranks IEF-11 as one of the most active MOF photocatalyst for OWS so far reported.

## 9 Efficiency and Stability of MOFs for OWS

### 9.1 Efficiency

One important parameter that should be taken into consideration when comparing the activity of different materials as photocatalysts is the photocatalyst weight dispersed in the liquid medium. Typically for suspended photocatalysts, there is an optimal weight of photocatalyst per liquid volume that results in the highest specific rate per photocatalyst mass. The use of excessive catalyst amount is detrimental due to the decrease in transparency of the liquid phase and the occurrence of scattering and poor transparency that limits light penetration in the liquid phase. On the other hand, low photocatalyst weights in the suspension do not guarantee complete light absorption. The operation of these two opposite effects determines the existence of an optimal photocatalyst concentration. Ideally, all of the measurements should be performed under optimal conditions. Besides, this general trend determines that the photocatalytic activity does not grow linearly with the photocatalyst weight in most of the mass scale.

Because the common practice in the field is to repeat OWS values for HER and OER as mmol g^–1^ h^–1^, it follows that this production rate is only valid for a certain catalyst amount, making difficult to compare efficiency of different photocatalysts obtained under not optimal conditions.

### 9.2 Water Stability

Because photocatalytic OWS requires as a prerequisite that the material is stable in this liquid medium, and there are several examples, as for instance MOF-5, MOF structures that are notoriously affected by water, the issue of water stability requires a specific comment. MOF lattice is formed by Coulombic bonds and metal–ligand coordination interaction. Both types of forces are strongly affected by water that on one hand can solvate ions, producing the isolations of anions and cations, and on the other hand, water can act as ligand competing with the organic linker in the framework. The issue of water stability is, therefore, particularly relevant when the lattice energy of MOF is low, a case more relevant for dipositive metal ions such as Zn^2+^, Cu^2+^, and Co^2+^ that tend to be in general unstable in aqueous solution. Much higher lattice energy and water stability is expected when the nodes have multiple positive charges as is the case of Fe^3+^-μ-O present in MIL-100 and MIL-101 among others that bears six positive charges or Zr_6_O_4_(OH)_4_ that bears 12 positive charges and is present in UiO-66 and other Zr-MOFs. In these examples, the water stability is relatively very large.^106,430−432^

Besides neutral water, the presence of acids/bases modifying the pH of the aqueous medium determines the presence of higher concentrations of H^+^, HO^–^, and the corresponding counterions that also affect considerably to MOF stability in water. In general, extreme pH values can destroy virtually any MOF. Fortunately, these extreme conditions are not normal for OWS that are ideally carried out in pure water, in quasineutral pH values.

Temperature can also affect MOF stability in water. Increasing the temperature above 50 °C can damage considerably MOF structure. There, however, are examples of MOFs that remain unaltered indefinitely in boiling water, without undergoing metal leaching or noticeable changes in the structure. Chemical reagents in high concentrations, particularly those that can form complexes such as amines, sulfides, and cyanide, to name a few, can also destroy the MOF structure. But, again, these examples are not expected to be present in photocatalytic OWS studies, although MOF stability in amines used as sacrificial electron donors has been an issue not sufficiently addressed in most studies. The reader is referred to existing literature on MOF stability in water and the existence of ultrastable MOFs in water for a more complete coverage of this important issue.

### 9.3 Photocatalyst Stability

Frequently, one of the main criteria to support MOF photostability is the coincidence of the XRD after the photocatalytic reaction with that of the fresh material. However, several examples have shown that XRD pattern exclusively is not sufficient, thus, to confirm MOF stability, the combination of several characterization and analytical techniques should be employed. In addition, very long irradiation times are necessary to convince of MOF photostability. Otherwise, MOF stability would be overestimated. For example, our group has shown that carboxylate-based MOFs such as MIL-101, etc., suffer partial CO_2_ decarboxylation upon irradiation with UV–vis at 20 °C, and this is not reflected by variations in the powder XRD patterns.^433^ For irradiation times in the order of months, up to one-third of the carboxylate groups of a MOF can be decomposed.

In other cases, MOFs can be unstable due to progressive ligand leaching. For example, when MIL-125(Ti)-NH_2_ was in contact with water. it can release the 2-aminoterephthalate organic ligand.^434^ It was believed that MIL-125-NH_2_ is gradually decomposed upon exposing in pure H_2_O, and completely decomposed after 4 h. Further, the photocatalytic activity of MIL-125-NH_2_ was decayed in the second cycle due to its instability under the optimized experimental conditions.^434,435^

In any case, considering any possible commercial implementation of MOFs, it is important to assess the long-term photostability under solar light and outdoor conditions, determining the main reasons for photocatalytic activity decay and devising doable reactivation methods.

## 10 Requirements for Large Scale OWS

Considering the current state of the art with technology readiness levels 2 or 3, the next step should be to prove the feasibility of MOFs as photocatalyst for solar OWS in the laboratory at larger scale in a demo plant.^16,37,58,68−70,436,437^ Scaling up MOFs as photocatalysts for solar-driven OWS should provide samples meeting some requirements. First, the MOF-based photocatalyst should be as efficient as possible and stable under the reaction conditions. STH efficiencies ranging from 4% to 5% measured under laboratory conditions should be reached in solar photoreactors as an intermediate step to meet a STH of 10% is probably the threshold required for any commercial application. These STH efficiencies should be achieved under continuous flow operation. A convenient method to deposit a thin layer of micrometric thickness of the MOF photocatalyst in the illuminated part of the photoreactor such as spray coating and thermal sintering should be developed. It can be expected that efficiency of these supported photocatalysts could decrease somewhat in comparison to stirred suspension.

Photocatalyst stability is another important requisite for technology feasibility. Initially, it can be considered that a MOF-based photocatalyst should be stable at least for 1000 h under ambient conditions, with a STH efficiency no lower than 5%. Studies on long-term photostability of MOFs are, therefore, necessary, because most current data refer to stability data of less than 24 h irradiation. In this context, it has been reported that some MOFs based on terephthalate ligand suffer partial decarboxylation upon irradiation with UV–vis light for several weeks, while other related MOFs, particularly imidazolates, appear much more photostable.^433^ Thus, similar studies even at longer times under reaction conditions are required to determine the potential use of MOFs as photocatalysts.

## 11 Conclusions and Outlook

In the context of the current decarbonization of energy, there is an urgent need to develop innovative, efficient technologies for green hydrogen generation from water. While water electrolysis is a mature technology with energy conversion efficiency over 60%, the high capital cost of electrolyzers and the high electricity cost is a major obstacle for massive implementation. In comparison, photocatalysis converting directly solar energy into hydrogen can be a very competitive and affordable technology for hydrogen production, provided that an efficient photocatalyst is developed. After more than 50 years of intensive research, a considerable number of materials and dyes have been screened as photocatalysts with only a limited progress. In this context, in a very short time, MOFs have reached an efficiency comparable to that obtained with inorganic semiconductors. Based on the performance of metallic complexes, the quantum efficiency for HER in the presence of sacrificial electron donors upon visible light irradiation can be close a 50%, much above the requirement for commercialization. Typical homogeneous systems are based on Ru-tripyridyl combined with methyl viologen as electron acceptor or even colloidal Pt NPs in suspension. Using tertiary amines as electron donors very high efficiency with AQE values over 20% can be achieved. In comparison, inorganic semiconductors are much less efficient, there being a compromise between a stability and toxicity with photocatalytic efficiency. Thus, CdS are among the most active photocatalyst for visible light HER with AQE values close to 20%. However, the use of Cd is not tolerable and stability for OWS is low. Metal nitrides such as tantalum nitride have also been reported as one of the most efficient photocatalyst, but Ta is a critical raw material.^58^ Considering all these factors, it seems that Al-doped SrTiO_3_ having adequate cocatalyst (RhCrO_*x*_, MoO_*y*_) with an efficiency about 0.6% of STH efficiency is the most convenient photocatalyst that can be considered as a benchmark to compare the performance of MOFs.^58^ It appears from the current state of the art, that MOFs have not yet reached the efficiency of the Domen benchmark (Rh-Cr_2_O_3_) strontium titanate photocatalyst. However, MOFs have the advantage that still several approaches to further increase photocatalytic efficiency can still be exploited to go beyond this value.

Although the field is still far from meeting the targets for commercialization, the progress has been very rapid and the number of tools and strategies for further improvement are very large, making promising, timely, and worthwhile to research the use of MOFs as solar photocatalysts for OWS. It has been shown that (i) the selection of MOF family and structure, (ii) the presence of substituents in the linker, (iii) the tuning of the composition of the metal nodes, particularly multimetallic nodes, (iv) the generation of an adequate density of defects, (v) the presence, location, and size of two cocatalysts, (vi) preferential facet growth and even 2D morphology, and (vii) particle size can increase the photocatalytic activity for OWS, and more efforts should be done to screen the various possibilities still unexplored. Similarly, heterojunction of MOFs with semiconductors of appropriate band alignment should render materials with increased charge separation efficiency and, therefore, with higher efficiency.

Overall, the MOFs offer the possibility to prepare materials by design and flexibility in the synthesis as well as postsynthetic modification that have shown their capability in tuning the optoelectronic and photochemical properties of these materials and, in particular, their impact on the photocatalytic efficiency for OWS. It is expected that this field will continue to flourish in the near future, hopefully achieving the goal of producing hydrogen from water directly from solar energy. When moving up in technology readiness level scale to values above 4, additional considerations besides photocatalytic efficiency such as cost-effective synthesis and simple preparation process will come into consideration.
